# Novel cinnamic acid-based *N*-benzyl pyridinium analogs: potent dual cholinesterase inhibitors with neuroprotective properties for Alzheimer's disease

**DOI:** 10.1039/d5ra06941f

**Published:** 2026-02-16

**Authors:** Maryam Esmkhani, Mohammad Mahdavi, Shahrzad Javanshir, Aida Iraji

**Affiliations:** a Pharmaceutical and Heterocyclic Compounds Research Laboratory, Department of Chemistry, Iran University of Science and Technology Tehran 16846-13114 Iran; b Endocrinology and Metabolism Research Center, Endocrinology and Metabolism Clinical Sciences Institute, Tehran University of Medical Sciences Tehran Iran; c Stem Cells Technology Research Center, Shiraz University of Medical Sciences Shiraz Iran; d Pharmaceutical Sciences Research Center, Shiraz University of Medical Sciences Shiraz Iran

## Abstract

This study reports the design and synthesis of a novel series of cinnamic acid-based analogs bearing an *N*-benzyl pyridinium moiety against Alzheimer's disease (AD), aiming at dual inhibition of acetylcholinesterase (AChE) and butyrylcholinesterase (BChE), alongside neuroprotective effects. A total of 15 derivatives were synthesized, among which compound 7b exhibited the most potent dual inhibition (AChE IC_50_ = 0.89 µM; BChE IC_50_ = 0.11 µM), and significant neuroprotection against H_2_O_2_-induced oxidative stress in SH-SY5Y cells, with no cytotoxicity under the tested concentration. Structure–activity relationship (SAR) analysis revealed that small electron-withdrawing substituents (*e.g. ortho*-fluoro, methyl) enhanced inhibitory activity, whereas *meta* and *para* substitutions generally reduced potency. Enzyme kinetics also determined compound 7b to be a competitive inhibitor of AChE (*K*_i_ = 0.49 µM). Furthermore, molecular docking and molecular dynamics simulations identified stable binding interactions in the active sites of AChE and BChE. All these findings support the potential of these compounds as effective multi-target-directed ligands (MTDLs) for AD, displaying coordinated inhibition of cholinesterase, neuroprotection, and low toxicity.

## Abstract 2

This study reports the design and synthesis of cinnamic acid-based analogs bearing an *N*-benzyl pyridinium moiety against Alzheimer's disease.

## Graphical abstract

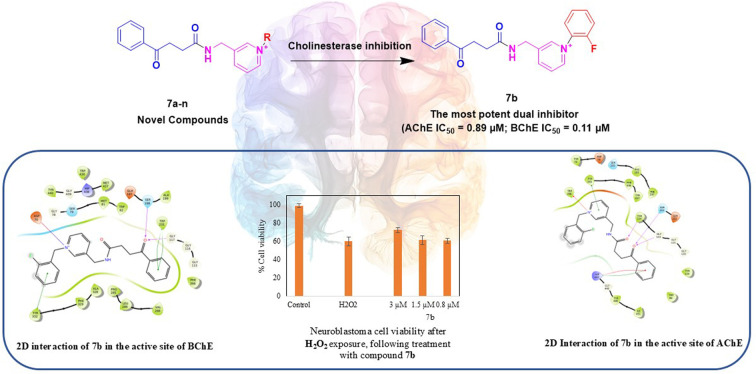

## 1. Introduction

Alzheimer's disease (AD) is a chronic, progressive brain disorder and the most prevalent cause of cognitive decline, responsible for about 60–80% of all cases of dementia. It is mostly found in people over 65 years of age, with the exception of the early presentation in some cases.^1^ It is associated with loss of memory, problems thinking, confusion, and changed behavior. Gradually, it leads to complete dependency and eventually death. At a biological level, AD has been linked to the generation of amyloid-beta (Aβ) plaques in synapses, tau protein tangles in neurons, inflammation, oxidative stress, and overall loss of nerve cells.^2–4^

Despite a history of research during these years, no definitive treatment for AD has been found.^5^ Current medications are intended to slow the rate of cognitive decline, emphasizing the importance of early diagnosis and timely treatment. It is approved that the cholinergic system is excessively damaged in AD.^6^ Acetylcholine (ACh), a neurotransmitter essential for memory formation and learning, is rapidly degraded by two enzymes acetylcholinesterase (AChE) and butyrylcholinesterase (BChE).^7^ Whereas AChE is more active in the early phases of AD, BChE becomes significant with disease progression. Thus, dual inhibition of AChE and BChE has been a promising approach to enhance cholinergic transmission and overcome cognitive manifestations. Some FDA-approved medications, such as donepezil, rivastigmine, and galantamine, are cholinesterase inhibitors that offer symptomatic relief with modest cognitive and functional improvement.^8^

In addition to cholinesterase inhibition, the neuroprotective medications aim to hinder or decelerate the loss of neurons by addressing oxidative stress, mitochondrial dysfunction, neuroinflammation, and excitotoxicity processes presently recognized as playing a major role in AD pathogenesis.^9,10^ Antioxidant and neuroprotective drugs are studied for their potential to halt or reverse neurodegenerative activity. The combination of cholinesterase inhibition with neuroprotection may provide a synergistic effect and not only symptomatic control but also modification of the disease.^11^ Hence, highly efficacious AChE and BChE inhibitors with minimal cytotoxicity and neuroprotection effects as categorized as the ideal therapeutic agents. In this context, various structural scaffolds have been explored, including iminochromene-2*H*-carboxamide [11], triazine [12], carbazole-benzylpiperidine [13], and *N*-cyclohexylimidazo[1,2-*a*]pyridine [14]. As a result, a novel class of cinnamic acid-derived analogues attached to benzyl pyridinium derivatives was designed to combine cognitive improvement, neuroprotection, and low toxicity in a single entity. Fifteen derivatives were synthesized and screened against AChE and BChE. Molecular docking, molecular dynamics simulations, kinetic analysis, cytotoxicity tests, and neuroprotection assays of the active analogues were performed and validate their therapeutic value towards AD.

## 2. Results and discussion

### 2.1 Designing

**Fig. 1:**
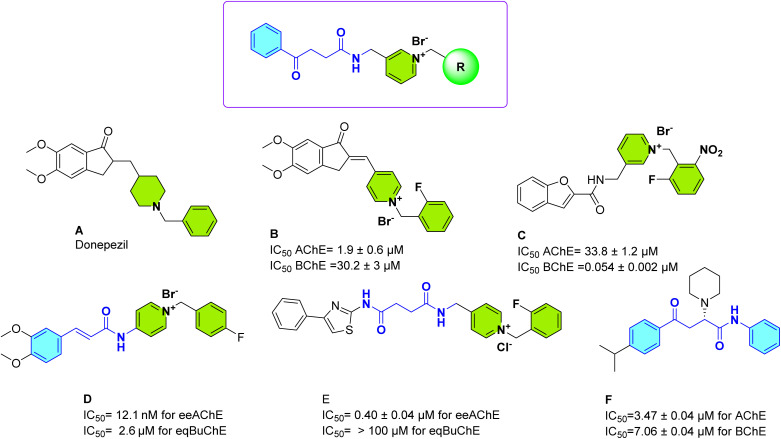
Designing strategy.

Donepezil (A, Fig. 1) is known as an FDA-approved drug for the management of AD. Considering this drug, different derivatives were designed and synthesized. A series of donepezil-based hybrids incorporating *N*-benzyl pyridinium groups (B, Fig. 1) was developed to enhance binding to both the catalytic active site (CAS) and peripheral anionic site (PAS) of AChE. These compounds demonstrated significant AChE inhibition, with structure–activity relationship (SAR) analysis highlighting that electron-donating groups, such as methoxy at the *para*-position of the benzyl ring, enhanced activity, likely due to strengthened π–π interactions with the PAS. A three-carbon spacer between the core and pyridinium ring was optimal for dual-site binding.^12^ Additionally, 2-benzofuran carboxamide derivatives (C, Fig. 1) with *N*-benzyl pyridinium moieties showed potent BChE inhibition (IC_50_ = 0.054–2.7 µM), with compound C being the most potent (IC_50_ = 0.054 µM), over 100 times stronger than donepezil, with excelled in AChE inhibition (IC_50_ = 2.1 µM). SAR indicated that 3-pyridinium derivatives outperformed 4-pyridinium ones, and *ortho*- or *meta*-substituted benzyl groups were more effective than *para*-substituted ones.^13^

Notably, cinnamic acid-based analogs with *N*-benzyl pyridinium groups, particularly compound D, exhibited exceptional AChE potency (IC_50_ = 12.1 nM for electric eel AChE; 8.6 nM for human AChE) with high selectivity over BChE (selectivity index ∼215). Structure activity relationships (SAR) showed that *meta* and *para* substituents fluorine or methoxy on the benzyl group improved potency, while bulky or electron-withdrawing groups reduced activity. Kinetic and docking studies confirmed D as a mixed-type inhibitor, engaging both CAS and PAS *via* π–π stacking, π–cation interactions, and hydrogen bonds.^14^ Thiazole-pyridinium hybrids (E, Fig. 1) demonstrated dual action against AChE and Aβ aggregation, with E showing strong AChE inhibition (IC_50_ = 0.40 µM) and superior Aβ inhibition compared to donepezil (20.4% and 42.7% *vs.* 14.7% at 10 µM). According to SAR, the *ortho* and *meta* substituted compounds demonstrated stronger inhibition against AChE than the *para* substituted derivatives. Compound E exhibited significant neuroprotection against H_2_O_2_-induced oxidative stress in PC12 cells, comparable to donepezil, and favorable blood–brain barrier permeability.^15^ For 4-aryl-4-oxo-*N*-phenyl-2-aminylbutyramides (F), SAR revealed that alkyl substituents near a hydrogen-bond acceptor, like a carbonyl enhanced AChE potency with heterocyclic amines (like piperidinyl) modulating activity and selectivity.^16^

In conclusion, combining cinnamic acid scaffolds with *N*-benzyl pyridinium groups emerged as an ideal pharmacophore for potent and selective AChE and BChE inhibition to target AD.

### 2.2 Synthesis

**Scheme 1:**
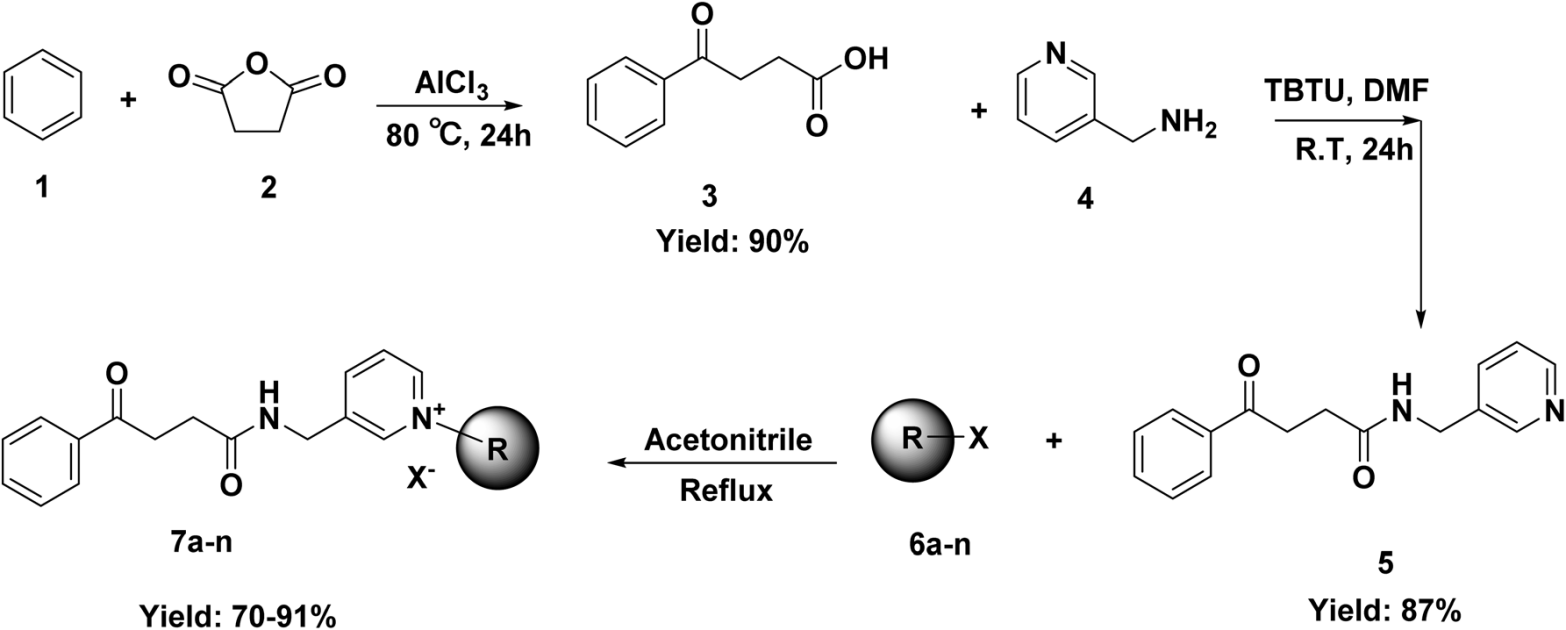
Synthesis of 7a–n.

The synthesis of 1-benzyl-3-[(4-oxo-4-phenylbutanamido)methyl]pyridine derivatives was carried out in three main steps shown in Scheme 1. Initially, 4-oxo-4-phenylbutanoic acid (3) was synthesized by reacting succinic anhydride (2) with anhydrous aluminum chloride in dry benzene (1) under reflux conditions for 24 hours. In the second step, the synthesized acid was activated using TBTU in the presence of triethylamine in DMF and then reacted with 3-picolylamine (4) at room temperature to form the corresponding amide, 4-oxo-4-phenyl-*N*-(pyridin-3-ylmethyl)butanamide (5). In the final step, this amide was dissolved in acetonitrile and reacted with different benzyl halide derivatives (6) under reflux. In each step the reaction completion was monitored *via* thin layer chromatography (TLC). These steps led to the successful synthesis of a series of substituted pyridine-based derivatives with potential biological significance.

### 2.3 Structure–activity relationship analysis of AChE inhibitors

The parent compound 7a (benzyl, AChE IC_50_ = 3.77 ± 0.55 µM) acts as our reference, showing a moderate level of AChE potency. Among fluoro substitutions analog, 7b (2-fluorobenzyl, AChE IC_50_ = 0.89 ± 0.12 µM) shows a notable improvement in potency compared to 7a, suggesting that the small, electronegative fluorine enhances binding through favorable electronic interactions with the residues in the AChE active site (Table 1).

**Table 1:** Inhibitory activity of 7a–n against AChE and BChE^a^

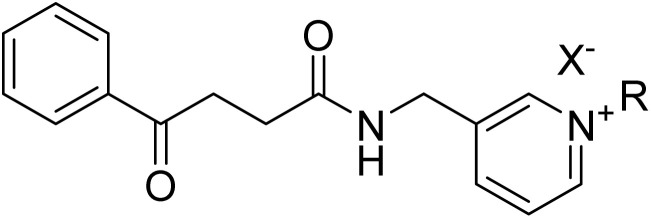					
Compound	R	X	IC_50_ (µM) against AChE	IC_50_ (µM) against BChE	Selectivity profile
7a	Benzyl	Br	3.77 ± 0.55	1.02 ± 0.29	3.69
7b	2-Fluorobenzyl	Br	0.89 ± 0.12	0.11 ± 0.08	8.09
7c	3-Fluorobenzyl	Cl	19.73 ± 0.96	0.49 ± 0.16	40.26
7d	4-Fluorobenzyl	Br	4.89 ± 1.41	11.09 ± 0.18	0.44
7e	2-Chlorobenzyl	Cl	4.04 ± 0.05	1.02 ± 0.00	3.69
7f	3-Chlorobenzyl	Cl	16.79 ± 0.27	0.98 ± 0.13	17.32
7g	4-Chlorobenzyl	Cl	24.55 ± 0.79	0.68 ± 0.26	36.10
7h	4-Bromobenzyl	Br	17.09 ± 2.77	2.66 ± 0.22	6.42
7i	4-Triflurobenzyl	Br	7.83 ± 2.75	6.61 ± 0.21	1.18
7j	4-Nitrobenzyl	Br	>50	25.22 ± 3.28	>1.98
7k	4-Methoxybenzyl	Br	20.43 ± 1.33	1.09 ± 0.03	18.73
7l	4-Methylbenzyl	Cl	13.21 ± 2.23	15.31 ± 0.24	0.86
7m	2-Methylbenzyl	Cl	15.17 ± 1.48	0.50 ± 0.16	30.34
7n	Methyl	I	0.90 ± 0.11	1.73 ± 0.31	0.51
Donepezil^b^	—		0.079 ± 0.05	10.6 ± 2.1	0.007

a Mean ± SE.

b Positive control.

Compound 7c (3-fluorobenzyl, AChE IC_50_ = 19.73 ± 0.96 µM) reduces potency considerably, which implies *meta*-fluoro disrupts AChE binding. Compound 7d (4-fluorobenzyl, AChE IC_50_ = 4.89 ± 1.41 µM) reduces potency modestly compared to 7a, which implies that *para*-fluoro is more tolerated than *meta*-fluoro but less favored than *ortho*-fluoro. Among chloro substitutions, 7e (2-chloro benzyl, AChE IC_50_ = 4.04 ± 0.05 µM) is marginally less potent than 7a, which suggests minimal steric hindrance by the chlorine atom. Compound 7f (3-chlorobenzyl, AChE IC_50_ = 16.79 ± 0.27 µM) is significantly less potent, suggesting unfavorable position at the *meta* position. Compound 7g (4-chlorobenzyl, AChE IC_50_ = 24.55 ± 0.79 µM) reduces the potency further, which suggests that *para*-chloro is not well tolerated for AChE binding, which may be due to steric or electronic mismatch. Compound 7h (4-bromobenzyl, AChE IC_50_ = 17.09 ± 2.77 µM) loses potency significantly, likely due to steric hindrance from the larger bromine atom. Compound 7i (4-trifluoromethylbenzyl, AChE IC_50_ = 7.83 ± 2.75 µM) is of moderate potency, with the electron-withdrawing hydrophobic trifluoromethyl group partially compensating for steric effects. Compound 7j (4-nitrobenzyl, AChE IC_50_ > 50 µM) is inactive due to the nitro group's strong electron-withdrawing nature and steric bulk. Compound 7k (4-methoxybenzyl, AChE IC_50_ = 20.43 ± 1.33 µM) reduces potency significantly, possibly because of the electron-donating nature or steric bulk of the methoxy group. Compound 7l (4-methylbenzyl, AChE IC_50_ = 13.21 ± 2.23 µM) reduces potency, indicating that *para*-methyl is less sterically disruptive than other *para* substitutions. Compound 7m (2-methylbenzyl, AChE IC_50_ = 15.17 ± 1.48 µM) is less active than 7a, revealing that *ortho*-methyl is responsible for steric hindrance.

On the other hand, compound 7n (methyl, AChE IC_50_ = 0.90 ± 0.11 µM) stands out with the highest AChE potency, hinting that the smaller, aliphatic methyl group boosts binding affinity, likely because it reduces steric hindrance or fits better in the AChE active site.

SAR analysis indicates that small substituents, such as the methyl group in 7n or *ortho*-fluoro in 7b are optimal for AChE inhibition, with 7n being the most potent. *Meta* and *para* substitution, particularly with larger or strongly electron-modifying groups such as chloro (7f, 7g), bromo (7h), nitro (7j), or methoxy (7k), reduce potency significantly. *Ortho* substitution is generally better tolerated, with fluoro being optimal. The trifluoromethyl group in 7i is moderately active but less than the unsubstituted benzyl. Optimization of AChE inhibition in the future should be aimed at small, electron-withdrawing ortho substituents or minimal alkyl groups for optimal potency.

The selectivity profile of the series for BChE over AChE is expressed as the selectivity index (SI = IC_50_ AChE/IC_50_ BChE). Most derivatives exhibited preferential inhibition of BChE, with SI values ranging from 3.69 to 40.26. Compound 7c (SI = 40.26), 7g (SI = 36.10), and 7m (SI = 30.34) displayed the highest BChE selectivity, while 7b showed excellent dual potency (IC_50_ AChE = 0.89 µM, IC_50_ BChE = 0.11 µM) with good BChE selectivity (SI = 8.09). In contrast, compounds 7d, 7l, and 7n showed slight preference for AChE (SI < 1).

### 2.4 Structure–activity relationship analysis of BChE inhibitors

Parent compound 7a (benzyl, BChE IC_50_ = 1.02 ± 0.29 µM, AChE IC_50_ = 3.77 ± 0.55 µM) is the baseline with good BChE activity and moderate AChE inhibition with a selectivity ratio (BChE IC_50_/AChE IC_50_) of 3.7, showing selectivity for BChE.

For comparison, 7n (methyl, BChE IC_50_ = 1.73 ± 0.31 µM, AChE IC_50_ = 0.90 ± 0.11 µM) has less BChE activity but more AChE activity, with the selectivity ratio of approximately 0.52 in favor of AChE. This suggests the benzyl skeleton is more favorable towards BChE inhibition, due to hydrophobic or π–π interactions in the BChE active site. Halogen substitutions provide hints about electronic and steric effects. Among the fluoro replacements, 7b (2-fluorobenzyl, BChE IC_50_ = 0.11 ± 0.08 µM, AChE IC_50_ = 0.89 ± 0.12 µM) is most potent at BChE with a selectivity ratio of approximately 8.09, meaning the small electronegative fluorine at the *ortho* position enhances binding *via* favorable interactions. Compound 7c (3-fluorobenzyl, BChE IC_50_ = 0.49 ± 0.16 µM, AChE IC_50_ = 19.73 ± 0.96 µM) increases the potency of BChE over 7a with a selectivity ratio of approximately 40.3, presenting very high selectivity towards BChE, but is less potent compared to 7b.

The compound 7d, characterized by 4-fluorobenzyl, has a BChE IC_50_ = 11.09 ± 0.18 µM, AChE IC_50_ = 4.89 ± 1.41 µM) significantly reducing BChE potency, with a selectivity ratio of approximately 0.44, favoring AChE, suggesting that *para*-fluoro disrupts BChE binding, possibly due to unfavorable electronic or steric effects. Regarding chloro substitutions, the compound identified as 7e (2-chlorobenzyl, BChE IC_50_ = 1.02 ± 0.00 µM, AChE IC_50_ = 4.04 ± 0.05 µM) maintains BChE potency equivalent to 7a, with a selectivity ratio of approximately 4.0.

Compound 7f (3-chlorobenzyl, BChE IC_50_ = 0.98 ± 0.13 µM, AChE IC_50_ = 16.79 ± 0.27 µM) slightly improves BChE potency, with a selectivity ratio of approximately 17.1, suggesting favorable electronic or steric interactions at the *meta* position. The compound 7g (4-chlorobenzyl, BChE IC_50_ = 0.68 ± 0.26 µM; AChE IC_50_ = 24.55 ± 0.79 µM), greatly increases the potency towards BChE. The selectivity ratio is about 36.1, which indicates that chlorine is well tolerated at the *para* position, possibly because of favorable electronic or hydrophobic interactions. Compound 7h (4-bromobenzyl, BChE IC_50_ = 2.66 ± 0.22 µM, AChE IC_50_ = 17.09 ± 2.77 µM) decreases BChE potency compared to 7a, with a selectivity ratio of approximately 6.4, likely due to the larger bromine causing steric hindrance. Compound 7i (4-trifluoromethylbenzyl, BChE IC_50_ = 6.61 ± 0.21 µM, AChE IC_50_ = 7.83 ± 2.75 µM) reduces BChE potency compared to 7a, with a selectivity ratio of approximately 1.2, but is better than other *para* substitutions like nitro or methyl, suggesting that the electron-withdrawing and hydrophobic trifluoromethyl group partially supports binding, though steric effects limit efficacy. Compound 7j shows a negligible enzyme inhibitory effect (selectivity greater than 2.0), likely due to the strong electron withdrawing group and steric bulk of the nitro group, which disrupted some important interactions (4-nitrobenzyl, BChE IC_50_ 25.22 ± 3.28 µM, AChE IC_50_ > 50 µM). On the other hand, compound 7k produced an inhibition pattern in some degree similar to that of 7a or nearly equal, which showed good potency for the inhibition of BChE, with an IC_50_ of 1.09 ± 0.03 µM, and poor inhibition for AChE, with an IC_50_ of 20.43 ± 1.33 µM, providing a selectivity ratio of approximately 18.7, thereby suggesting that the *para*-position of the electron-donating methoxy group is beneficial. Compound 7l (4-methylbenzyl, BChE IC_50_ = 15.31 ± 0.24 µM, AChE IC_50_ = 13.21 ± 2.23 µM) significantly reduces BChE potency, with a selectivity ratio of approximately 0.86, indicating that the *para*-methyl group is poorly tolerated, possibly due to steric effects. Compound 7m (2-methylbenzyl, BChE IC_50_ = 0.50 ± 0.16 µM, AChE IC_50_ = 15.17 ± 1.48 µM) improves BChE potency compared to 7a, with a selectivity ratio of approximately 30.3, suggesting that the *ortho*-methyl group enhances hydrophobic or van der Waals interactions without significant steric hindrance.

SAR pointed at small electron-withdrawing substituents mostly, on the *ortho* position, like fluoro in 7b, and small hydrophobic groups, like methyl in 7m were best for BChE inhibition, often with high selectivity against AChE. Fluoro in 7c and chloro in 7f at the *meta* position increase BChE potency and selectivity as well. *Para* substitution is mostly disfavored, except chloro in 7g and methoxy in 7k, which still show good potency and selectivity. On the other hand, bulky or strong electron-modifying groups at the *para* position, such as nitro in 7j and bromo in 7h, reduce the BChE activity. The benzyl scaffold was generally found to be preferred for BChE, whereas methyl in 7n prefers AchE, which could be due to the bulkier active site of BChE.

### 2.5 Kinetic study

**Fig. 2:**
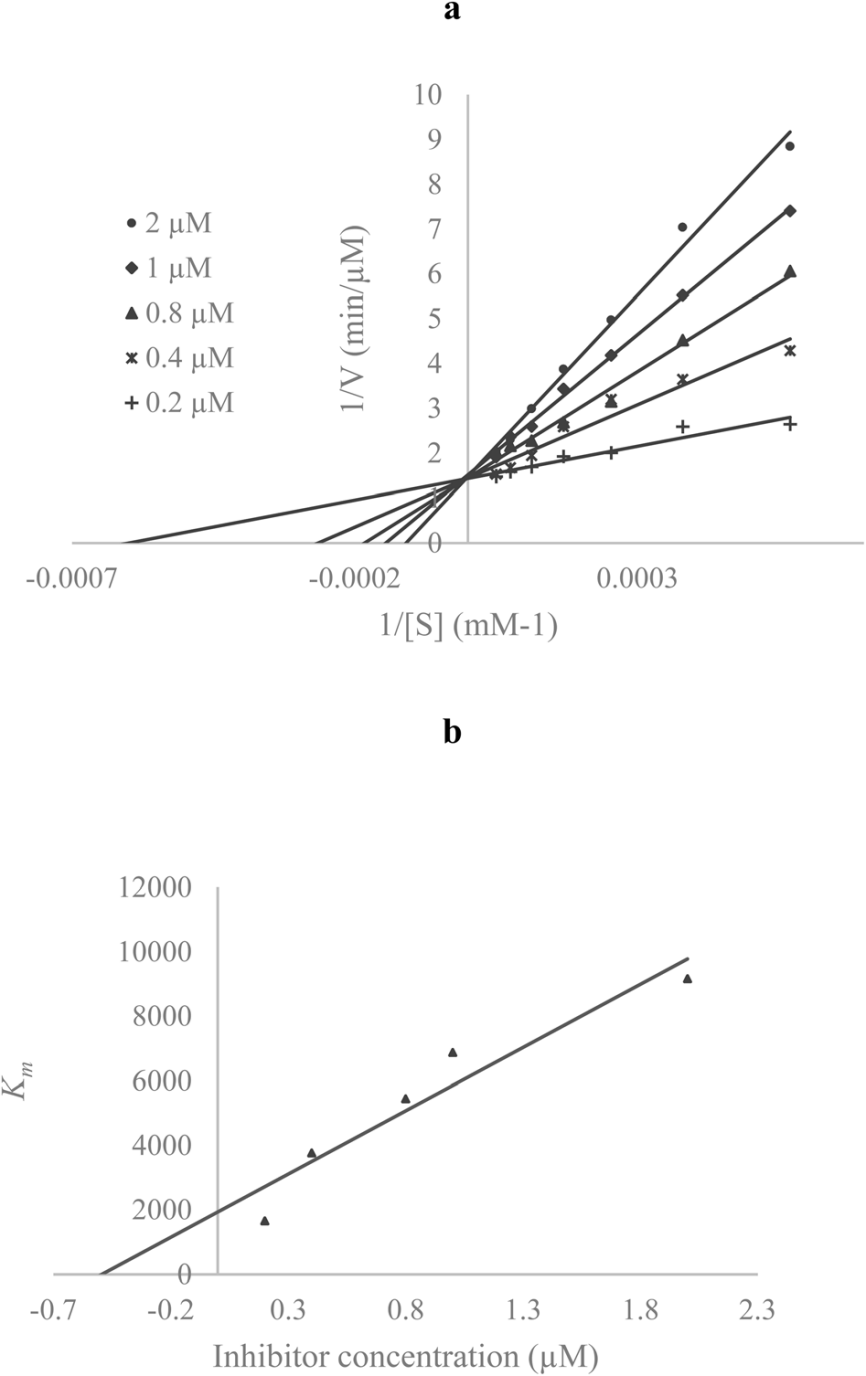
(a) The Lineweaver–Burk plot of the 7b against AChE, (b) the secondary plot between *K*_m_ and various concentrations of 7b.

To determine the mechanism of inhibition, a kinetic study of 7b as a potent inhibitor was done against AChE. In the reciprocal Lineweaver–Burk plot (Fig. 2a), the *K*_m_ gradually increased and *V*_max_ remained unchanged with increasing inhibitor concentration, indicating a competitive inhibition. Furthermore, the plot of the *K*_m_*versus* different concentrations of the inhibitor gave an estimate of the inhibition constant, *K*_i_ of 0.49 µM (Fig. 2b).

### 2.6 Cell cytotoxicity and neuroprotection

**Fig. 3:**
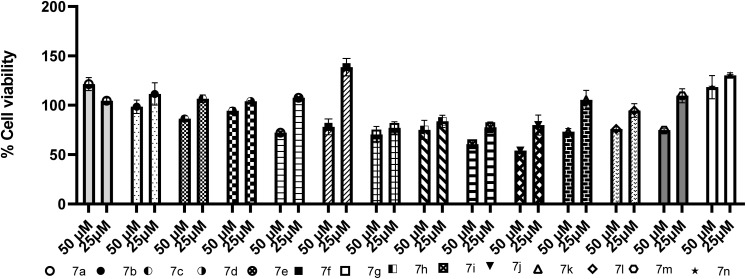
Cytotoxicity study of 7a–n against the SH-SY5Y.

Cell viability of all derivatives was assessed on the SH-SY5Y neuroblastoma cell line 72 h after treatment, and the results are summarized in Table 2 and Fig. 3.

**Table 2:** CC_50_ and selectivity indices of compounds 7a–n

Compound	CC_50_ (µM)	SI *vs.* AChE (CC_50_/IC_50_ AChE)	SI *vs.* BChE (CC_50_/IC_50_ BChE)
7a	121.48 ± 6.52	29.6	118.6
7b	111.46 ± 11.22	97.1	1013
7c	86.42 ± 2.91	4.3	176.4
7d	94.42 ± 3.31	19.3	8.5
7e	72.23 ± 1.68	17.8	70.8
7f	78.11 ± 7.96	4.6	79.7
7g	70.35 ± 8.18	2.86	103.4
7h	75.08 ± 9.59	4.4	28.2
7i	60.66 ± 3.57	7.74	9.2
7j	54.02 ± 3.51	—	2.1
7k	73.26 ± 3.11	3.6	67.2
7l	75.98 ± 2.14	5.7	4.96
7m	74.94 ± 1.32	4.94	149.9
7n	118.35 ± 11.81	131	86.4

From the data, most compounds show low cytotoxicity at both concentrations. At 50 µM, compounds 7j (54.0%) and 7i (60.7%) exhibited the strongest reduction in cell viability, while 7g, 7e, 7f, 7k, 7m, 7h, and 7l reduced viability to approximately 70–76%, indicating moderate effects. Compounds 7c, 7d, and others remained above 86% viability, and remarkably, 7n (118.3%) and 7a (121.5%) slightly increased viability compared to control, which may reflect mild proliferative stimulation. At the lower 10 µM concentration, nearly all compounds displayed viability above 77–138%, with several (7n, 7f, 7m, 7a, 7e, 7k, 7c) exceeding 100%, again suggesting good tolerability and possible metabolic activation at lower doses.

To assess the therapeutic window, selectivity indices (SI) and CC_50_ were calculated (Table 2). The lead compound 7b demonstrated exceptionally high selectivity, particularly against BChE (SI = 1013), along with a strong SI of 97.1 against AChE, underscoring its favorable profile as a potent dual inhibitor with minimal neurotoxicity. Among the series of several derivatives, including 7a, 7c, and 7m achieve SI > 100 for at least one enzyme, considering these analogs as ideal lead candidates.

**Fig. 4:**
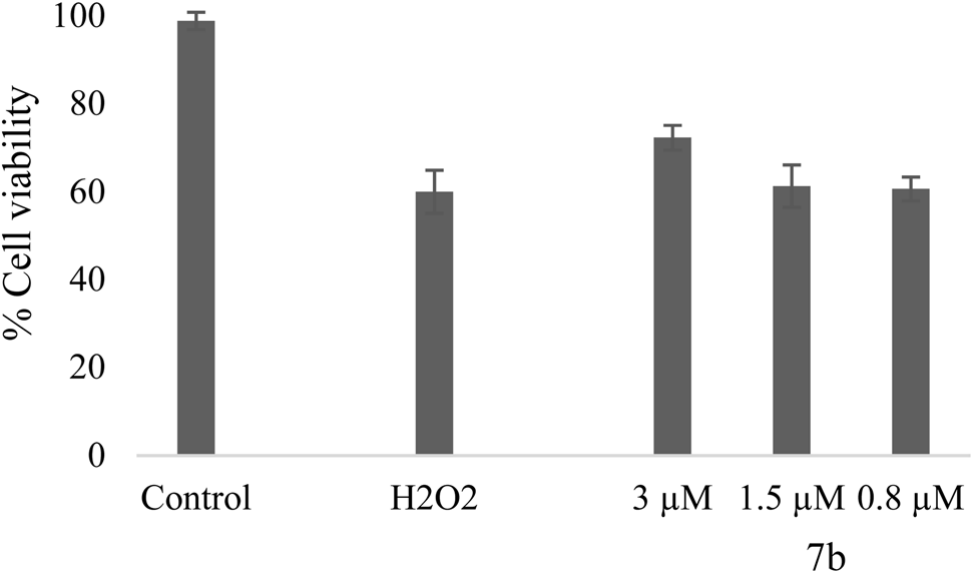
Neuroblastoma cell viability after H_2_O_2_ exposure, following treatment with compound 7b.

The ability of compound 7b to protect against neurotoxicity was tested in SH-SY5Y cells under oxidative stress induced by hydrogen peroxide (H_2_O_2_). H_2_O_2_ exposure caused cell viability to drop to about 59% compared to controls. Treatment with compound 7b at 3 µM produced a pronounced neuroprotective effect, resulting in a significant increase in viability to 72% (Fig. 4).

### 2.7 Molecular docking

**Fig. 5:**
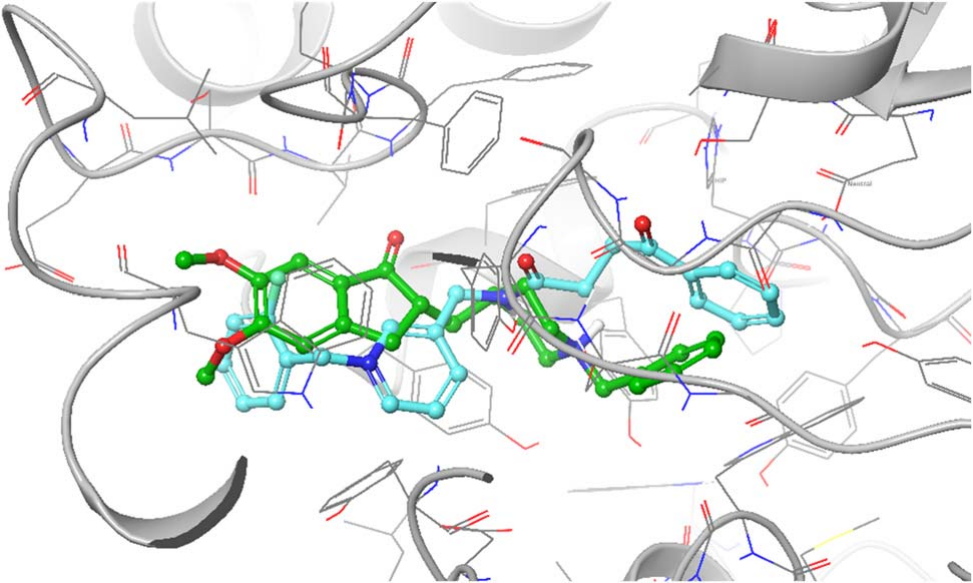
Superimposed binding poses of compound 7b (blue) and donepezil (green) within the active site of AChE.

Docking studies were conducted to evaluate the interaction of compounds with AChE. The suitability of the docking protocol was assessed by redocking the native ligand into the AChE active site, yielding root-mean-square deviation (RMSD) values below 2 Å, thereby validating the docking process. The superimposed structure of donepezil and compound 7b is exhibited in Fig. 5. It is shown that 7b has a binding pose similar to that of donepezil within the active site. Compound 7b recorded a binding energy of −9.43 kcal mol^−1^, indicating high binding affinity. The cinnamic acid moiety of 7b exhibited π–π stacking and π–cation interactions with Hip447 (protonated form of His residue), a key residue of the catalytic triad that plays a role in inhibiting AChE. Furthermore, the carbonyl group formed hydrogen bonds with Ser203, a catalytic triad residue, and with Glh122 (protonated Glu). The carbonyl of the amide linker also donated a hydrogen bond with Tyr124 in the PAS. Furthermore, the terminal 2-fluorobenzyl pyridinium was involved in π–π stacking interactions with Tyr341 and π–cation interactions with Trp286, both of which are located in the PAS region and are important in ligand recognition and inhibition.

To elucidate the influence of substituent position on binding behavior, additional docking studies were performed for analogs 7c–g. The 7c (3-fluorobenzyl) exhibited a lower binding energy (−8.608 kcal mol^−1^) and showed π–π stacking with Tyr337 and Trp86, as well as hydrogen bonding to Phe295, suggesting weaker stabilization within the active site than 7b. In contrast, the *para* substituted analog 7d displayed a slightly improved binding energy (−9.559 kcal mol^−1^), forming π–π stacking with Trp86 and Tyr341, as well as hydrogen bonds with Phe295.

Among the chloro-substituted derivatives, 7e bearing *ortho*-chloro substitution showed the strongest binding energy (−10.559 kcal mol^−1^), likely due to enhanced hydrophobic interactions and ideal pose within the binding pocket. In comparison, *meta* (7f) and *para* chloro (7g) analogs displayed slightly reduced binding energies, indicating that *ortho* substitution better stabilization within the AChE active site (Table 3).

**Table 3:** Molecular docking results of donepezil compared with selected compounds against AChE and BChE

Compound	AChE		BCHE	
	Binding energy (kcal mol^−1^)	Type of interaction and residues	Binding energy (kcal mol^−1^)	Type of interaction and residues
Donepezil	−9.342	π–cation with Trp86	−7.590	H-bond with Ser198
		π–cation with Tyr337		π–π stacking with Trp82
		π–cation with Phe338		π–cation with Tyr332
		π–cation with Tyr341		
		Salt bridge with Asp74		
		H-bound with Phe295		
		π–π stacking Trp286		
7b	−9.43	π–π stacking Hip447	−9.04	Two π–π stacking with Trp231
		π–cation with Hip447		H-bond with Ser198 H-bond with Gly117
		H-bond with Ser203		π–cation with Asp70
		H-bond with Glh122		π–π stacking with Tyr332
		H-bond with Tyr124		
		π–cation with Trp286		
7c	−8.608	π–π stacking with Tyr337	−8.641	π–π stacking with Trp332
		π–π stacking with Trp86		π–cation with Phe329
		π–cation with Trp86		π–π stacking with Trp430
		H-bond with Phe295		H-bonding with His438
		π–π stacking with Trp286		
7d	−9.559	π–π stacking with Trp86	−7.919	π–π stacking with Trp82
		π–π stacking with Tyr341 a π–cation with Asp74		π–cation with Asp70
		Two H-bonds with Phe295		H-bond with Ser198

**Fig. 6:**
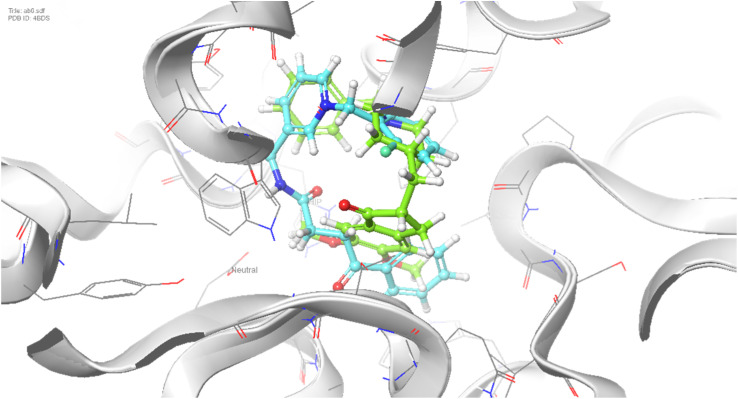
Superimposed binding poses of compound 7b (blue) and donepezil (green) within the active site of BChE.

Molecular docking was performed against BChE, and the binding poses of compound 7b and donepezil are shown in Fig. 6. The aromatic ring of the cinnamic moiety in compound 7b formed two π–π stacking interactions with Trp231. Additionally, the carbonyl group of the cinnamic acid formed two hydrogen bonds, one with Ser198 (a key residue of the catalytic triad) and the other with Gly117 (located in the oxyanion hole); both are crucial for catalytic function. The pyridinium moiety underwent a π–cation interaction with Asp70, while the terminal 2-fluorobenzyl group performed a π–π stacking interaction with Tyr332 instead, which relates to PAS of BChE.These findings highlight the dual binding potential of compound 7b, showing effective interactions with both the CAS and the PAS in both cholinesterase enzymes.

Compound 7c (3-fluorobenzyl) exhibited π–π stacking with Trp332, a pyridinium π–cation interaction with Phe329, additional π–π stacking with Trp430, and hydrogen bonding with His438. In 7d (4-fluorobenzyl), π–π stacking occurred with Trp82, the pyridinium π–cation interacted with Asp70, and a hydrogen bond was formed with Ser198. The chloro-substituted compounds 7e–g exhibited approximately similar binding energy, suggesting that the substituent position does not significantly affect overall binding affinity against BChE.

### 2.8 Molecular dynamics simulations

**Fig. 7:**
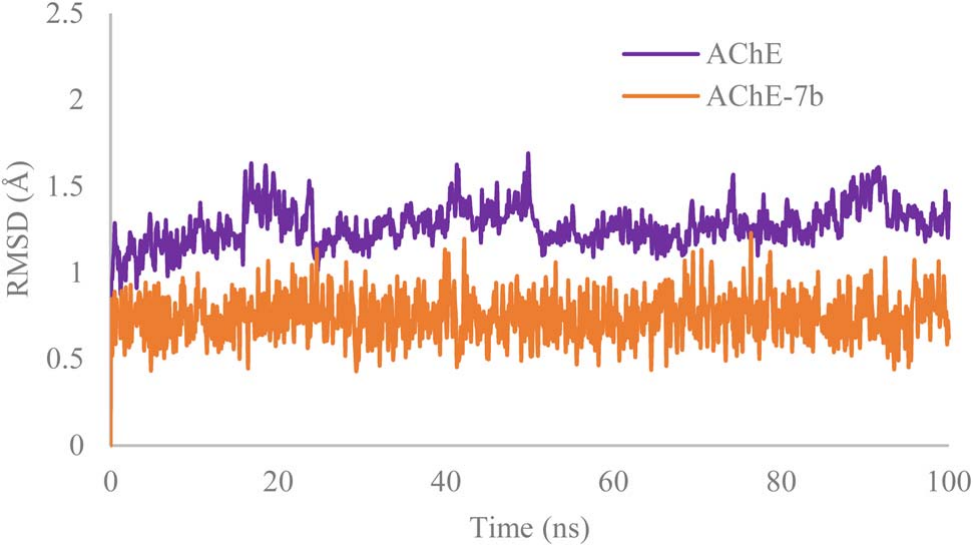
RMSD plot of AChE backbone (Apo, velvet color) and compound 7b (orange) throughout the 100 ns of the simulation time.

Molecular dynamics simulations were performed to evaluate the stability of the AChE–7b complex, compared with apo AChE form. The RMSD fluctuations of AChE were natural, with an average of 1.2 Å. On the contrary, the RMSD of the AChE–7b complex was much less compared to the unbound state, averaging only 0.6 Å. This highlights the stability of AChE–7b complex, thus making compound 7b a prospective strong AChE inhibitor (Fig. 7).

**Fig. 8:**
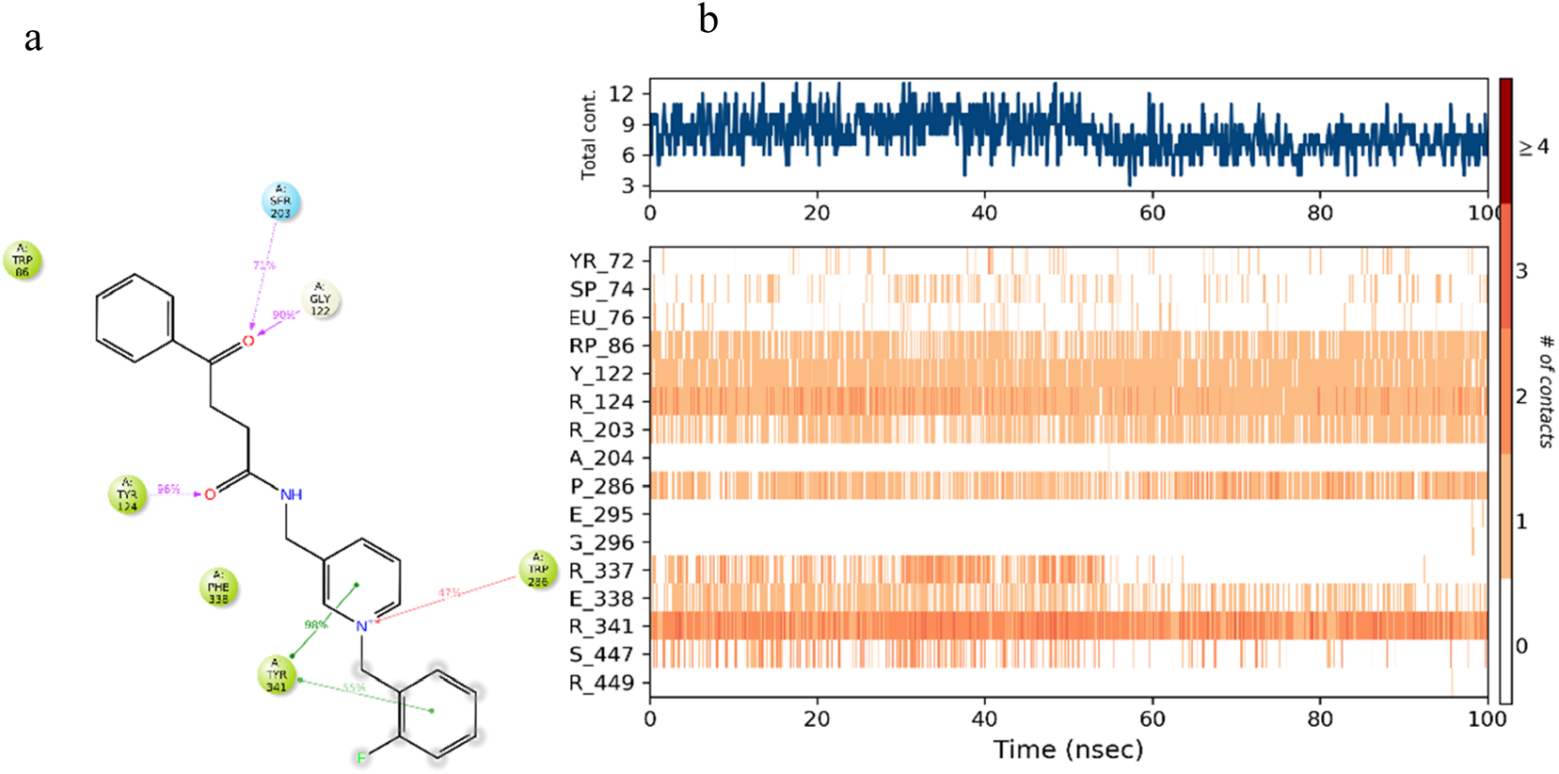
(a) 2D interaction diagram and (b) timeline interaction of compound 7b-AChE complex, which is responsible for over 30% of MD simulation time.

The interactions of compound 7b within AChE active site were further explored by MD simulation; the 4-oxo-4-phenylbutanamido moiety formed three hydrogen bonds with Gly122, Tyr124, and Ser203 (Fig. 8a). A persistent hydrogen bond was evidenced to Tyr124 and was present for about 96% of the simulation time. The pyridinium linker stabilized the complex through π–cation interaction with Trp286 and through π–π stacking with Tyr341, both residues being located at the PAS. RMSF analysis of AChE in complex with 7b showed most residues below 2 Å RMSF, confirming protein structural stability over simulation. These interactions were preserved across MD snapshots, providing further evidence for the stability of the AChE–7b complex (Fig. 8b).

**Fig. 9:**
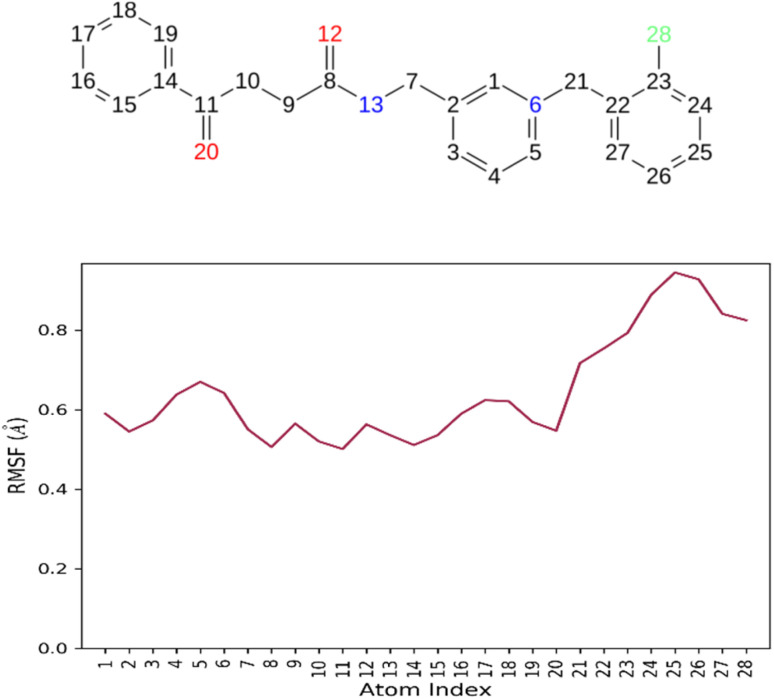
RMSF of compound 7b in the AChE active site.

The RMSF analysis of compound 7b is carried on inside the AChE active site. The RMSD less than 2 Å indicates proper fitting in the active site. All atoms of 7b recorded RMSF values of less than 1 Å, showing that the ligand is properly anchored in the active site. Such stable binding ensured that, throughout the simulation, both CAS and PAS sites on the enzyme were occupied, making it a potential candidate for an efficient inhibitor (Fig. 9).

### 2.9 *In silico* drug-likeness, ADME, and toxicity studies

**Fig. 10:**
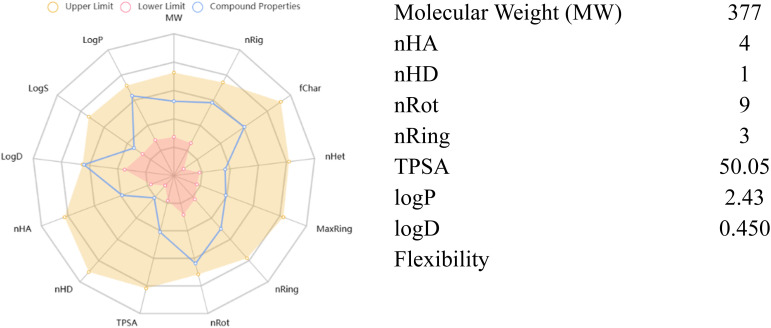
Physicochemical properties of 7b.

**Fig. 11:**
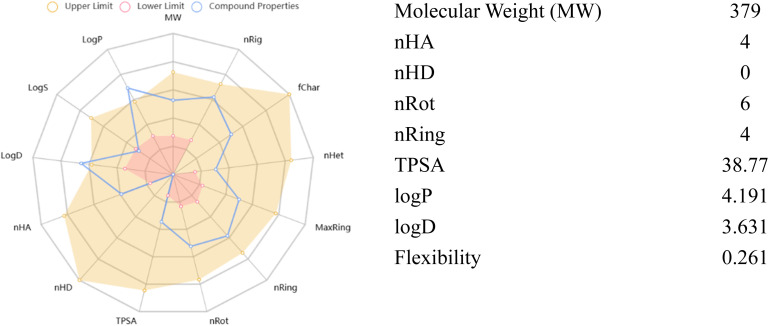
Physicochemical properties of donepezil.

*In silico* ADMET profiling of the most potent compound 7b (Fig. 10 and Table 4) revealed a highly favorable profile relative to the reference drug, donepezil (Fig. 11 and Table 5). Both compounds fulfil Lipinski's rule with no violations, but 7b exhibits lower lipophilicity (log *P* 2.43 *vs.* 4.19), higher TPSA (50.05 *vs.* 38.77 Å^2^), and full compliance with the Pfizer 3/75 rule compared with donepezil. Compound 7b shows good intestinal absorption, comparable Caco-2 permeability, markedly lower plasma protein binding (34.51% *vs.* 87.74%), similar volume of distribution, reduced CYP substrate liability, slightly lower clearance, longer half-life, and no predicted mutagenicity, carcinogenicity, or skin sensitization overall indicating superior drug-like properties, lower drug–drug interaction risk, and potentially improved brain availability compared to donepezil, thereby supporting 7b as a promising and safe anti-AD.

**Table 4:** ADME-T properties of 7b

	Parameters	Value		Parameters	Value
Medicinal chemistry	Lipinski rule	Accepted	Distribution	PPB	34.51
	Pfizer rule	Accepted		VD (volume distribution)	1.685
	SAscore	Accepted	Metabolism	CYP1A2 inhibitor	—
Absorption	Caco-2 permeability	−5.112		CYP1A2 substrate	—
	HIA (human intestinal absorption)	+		CYP2C9 inhibitor	—
Toxicity	Carcinogenicity	—		CYP2C9 substrate	—
	AMES toxicity	—		CYP2D6 inhibitor	+
	Skin sensitization	—		CYP2D6 substrate	—
	LC50FM	3.323	Excretion	CL (clearance rate)	9.206
	LC50DM	4.724		T1/2	0.827

**Table 5:** ADMET properties of donepezil

	Parameters	Value		Parameters	Value
Medicinal chemistry	Lipinski rule	Accepted	Distribution	PPB	87.74
	Pfizer rule	Rejected		VD (volume distribution)	1.589
	SAscore	Accepted	Metabolism	CYP1A2 inhibitor	—
Absorption	Caco-2 permeability	−4.793		CYP1A2 substrate	+
	HIA (human intestinal absorption)	—		CYP2C9 inhibitor	—
Toxicity	Carcinogenicity	—		CYP2C9 substrate	—
	AMES toxicity	—		CYP2D6 inhibitor	+
	Skin sensitization	—		CYP2D6 substrate	+
	LC50FM	5.338	Excretion	CL (clearance rate)	10.635
	LC50DM	6.367		T1/2	0.164

## 3. Conclusion

The findings of the current research demonstrate effective design and synthesis of a novel series of cinnamic acid analogues conjugated with *N*-benzyl pyridinium, which are potential dual inhibitors of AChE and BChE and exhibit excellent neuroprotective activity. Compound 7b demonstrated promising inhibitory potential with IC_50_ = 0.89 µM against AChE and IC_50_ = 0.11 µM against BChE. SAR research demonstrated that electron-withdrawing small substituents, like *ortho*-fluoro groups, substantially elevate potency and selectivity, while bulk or electron-donating *meta*- and *para*-substitutions reduce potency. Kinetic assays confirmed 7b as a competitive AChE inhibitor (*K*_i_ = 0.49 µM), and molecular docking and molecular dynamics simulations supported its stable binding to both CAS and PAS of AChE and BChE *via* π–π stacking, π–cation interactions, and hydrogen bonding. Furthermore, this study highlights 7b low cytotoxicity to SH-SY5Y neuroblastoma cells at 10 µM and a potent neuroprotective effect against oxidative stress induced by H_2_O_2_. Overall, the current study showed cinnamic acid-derived *N*-benzyl pyridinium analogues as promising MTDLs of AD with optimal cognitive enhancement, neuroprotection, and safety.

## 4. Methods and materials

All the solvents and reagents were purchased from Merck Chemical Company and used without further purification. The melting points were determined using an Electrothermal 9100 instrument. The ^1^HNMR and ^13^CNMR spectra were recorded with a Bruker 500 using TMS as an internal standard. Chemical shifts were reported at room temperature in ppm scale with DMSO-*d*_6_ as the solvents due to the poor solubility of the salts in non-polar solvents. SH-SY5Y cells were obtained from the Pasteur Institute of Iran (https://en.pasteur.ac.ir/).

### 4.1 Chemistry

#### 4.1.1 Synthesis of 4-oxo-4-phenylbutanoic acid

Succinic anhydride (1.1 g, 11 mmol) was dissolved in 100 mL of anhydrous benzene under vigorous stirring. Subsequently, anhydrous aluminum chloride (2.66 g, 20 mmol) was carefully added to the reaction mixture. The resulting suspension was refluxed and the progress of the reaction was monitored by thin-layer chromatography (TLC). After 24 hours, the solvent was evaporated and the reaction was quenched by the slow addition of 100 mL of distilled water. The pH of the mixture was basified to pH 9–10 using 0.1 M aqueous sodium hydroxide. The clear filtrate was then acidified to pH 1–2 with dilute HCl to precipitate the free 4-oxo-4-phenylbutanoic acid, which was collected by filtration, washed with cold water, and dried at 60 °C for subsequent use.

#### 4.1.2 Synthesis of 4-oxo-4-phenyl-*N*-(pyridin-3-ylmethyl)butanamide

A solution of the previously synthesized 4-oxo-4-phenylbutanoic acid (0.356 g, 2 mmol) was prepared in 4 mL of dimethylformamide (DMF), followed by the addition of triethylamine as a base. The reaction mixture was stirred for 20 minutes at room temperature, then TBTU (0.642 g, 2 mmol) was added as the coupling reagent. After 30 minutes, 3 mmol of 3-picolylamine (0.324 g) was added to the mixture, and stirring was continued at ambient temperature. The progress of the reaction was monitored by thin-layer chromatography (TLC). After completion, the reaction was cooled, and water was added slowly. A solid precipitate, which was collected by filtration and oven-dried. The crude material was recrystallized from ethyl acetate/methanol (1 : 1) to give the pure compound 5 as an off-white solid (yield: 0.233 g, 87%).

#### 4.1.3 Synthesis of 1-benzyl-3-[(4-oxo-4-phenylbutanamido)methyl]pyridine derivatives

For the synthesis of these derivatives, 1 mmol of the previously synthesized amide (0.268 g) was dissolved in 10 mL of acetonitrile, followed by the addition of 2 mmol of benzyl halide derivatives. The reaction mixture was refluxed under stirring. Upon confirmation of reaction completion by thin-layer chromatography, the solvent was evaporated to induce precipitation. The resulting salt was collected by filtration and washed with ethyl acetate to enhance the purity of the final product. The obtained salts are freely soluble in DMSO, DMF and water, moderately soluble in acetonitrile and methanol, and poorly soluble in ethyl acetate. All compounds were obtained as quaternary *N*-benzylpyridinium halide salts (X = Cl, Br) and were characterized by NMR spectroscopy and melting point determination.

##### 4.1.3.1 1-Benzyl-3-((4-oxo-4-phenylbutanamido)methyl)pyridin-1-ium bromide (7a)

Yield: 85%. Cream powder. ^1^H NMR (400 MHz, DMSO) *δ* 9.20 (d, *J* = 6.4 Hz, 2H, (H-2,6 pyridinium)), 8.85 (t, *J* = 5.9 Hz, 1H, (*N*–H amide)), 8.55–8.48 (m, 1H), 8.18 (dd, *J* = 8.1, 6.0 Hz, 1H), 8.02–7.95 (m, 2H), 7.65 (td, *J* = 7.2, 1.3 Hz, 1H), 7.61–7.49 (m, 4H), 7.42 (dd, *J* = 5.1, 1.9 Hz, 3H), 5.92 (s, 2H, CH_2_–N^+^ (benzyl)), 4.50 (d, *J* = 5.8 Hz, 2H, (–CH_2_–CONH)), 3.33 (t, *J* = 6.4 Hz, 2H, (CH_2_–CO–Ph)), 2.62 (t, *J* = 6.4 Hz, 2H, (CH_2_–CH_2_–CO)) ppm. ^13^C NMR (101 MHz, DMSO) *δ*: 29.56, 33.84, 39.36, 39.56, 39.77, 39.81, 39.98, 40.19, 40.40, 40.61, 63.76, 128.35, 128.52, 129.21, 129.66, 129.83, 133.73, 134.79, 136.90, 141.71, 143.37, 143.65, 144.66, 172.76 (CONH), 199.68 (C=O) ppm.

##### 4.1.3.2 1-(2-Fluorobenzyl)-3-((4-oxo-4-phenylbutanamido)methyl)pyridin-1-ium bromide (7b)

Yield: 70%, dark yellow solid. ^1^H NMR (500 MHz, DMSO) *δ* 9.08 (s, 1H, (H-2 pyridinium)), 9.03 (d, *J* = 6.1 Hz, 1H, (H-6 pyridinium)), 8.93 (t, *J* = 5.9 Hz, 1H, (N–H amide)), 8.56 (d, *J* = 8.0 Hz, 1H), 8.17 (dd, *J* = 8.1, 6.0 Hz, 1H), 7.97–7.92 (m, 2H), 7.65–7.60 (m, 1H), 7.59–7.44 (m, 6H), 7.41 (td, *J* = 7.3, 1.4 Hz, 1H), 6.04 (s, 2H, CH_2_–N^+^ (benzyl)), 4.49 (d, *J* = 5.8 Hz, 2H, (–CH_2_–CONH)), 3.29 (t, *J* = 6.5 Hz, 2H, (CH_2_–CO–Ph)), 2.59 (t, *J* = 6.5 Hz, 2H, (CH_2_–CH_2_–CO)) ppm, *δ*^13^CNMR (126 MHz, DMSO): 29.16, 33.39, 39.11, 39.27, 39.36, 39.44, 39.57, 39.61, 39.77, 39.94, 40.11, 61.23, 125.75, 127.80, 127.89, 128.07, 128.65, 130.01, 131.34, 131.37, 133.13, 133.21, 136.47, 141.11, 143.41, 143.52, 144.69, 172.20 (CONH), 199.04 (C=O) ppm, HRMS (ESI) (*m*/*z*): found for C_23_H_22_FN_2_O_2_^+^ [M + H]^+^ 377.0838.

##### 4.1.3.3 1-(3-Fluorobenzyl)-3-((4-oxo-4-phenylbutanamido)methyl)pyridin-1-ium chloride (7c)

Cream solid; yield: 73%, ^1^H NMR (500 MHz, DMSO) *δ* 9.13 (d, *J* = 6.8 Hz, 2H, (H-2,6 pyridinium)), 8.73 (t, *J* = 5.9 Hz, 1H, (N–H amide)), 8.50 (d, *J* = 8.1 Hz, 1H), 8.17 (dd, *J* = 8.1, 5.8 Hz, 1H), 7.99–7.93 (m, 2H), 7.63 (t, *J* = 7.3 Hz, 1H), 7.55–7.41 (m, 4H), 7.38 (d, *J* = 7.6 Hz, 1H), 7.25 (td, *J* = 8.6, 2.7 Hz, 1H), 5.89 (s, 2H, CH_2_–N^+^ (benzyl)), 4.49 (d, *J* = 5.9 Hz, 2H, (–CH_2_–CONH)), 3.31 (t, *J* = 6.5 Hz, 2H, (CH_2_–CO–Ph)), 2.59 (t, *J* = 6.4 Hz, 2H, (CH_2_–CH_2_–CO)) ppm, *δ*^13^C NMR (101 MHz, DMSO): 29.52, 33.80, 39.34, 39.54, 39.65, 39.75, 39.96, 40.17, 40.38, 63.05, 116.88, 125.51, 128.34, 128.57, 129.20, 131.77, 131.77, 133.75, 139.47, 141.78, 143.49, 144.78, 172.77 (CONH), 190.12 (C=O) ppm.

##### 4.1.3.4 1-(4-Fluorobenzyl)-3-((4-oxo-4-phenylbutanamido)methyl)pyridin-1-ium bromide (7d)

Cream solid; yield: 76%, ^1^H NMR (400 MHz, DMSO) *δ* 9.24–9.17 (m, 2H, (H-2,6 pyridinium)), 8.80 (t, *J* = 5.9 Hz, 1H, (N–H amide)), 8.56–8.49 (m, 2H), 8.27 (dd, *J* = 8.1, 2.1 Hz, 1H), 8.20 (dd, *J* = 8.1, 6.0 Hz, 1H), 8.02 (dd, *J* = 7.8, 1.3 Hz, 1H), 7.98–7.94 (m, 2H), 7.73 (t, *J* = 8.0 Hz, 1H), 7.67–7.62 (m, 1H), 7.53 (t, *J* = 7.7 Hz, 2H), 6.04 (s, 2H CH_2_–N^+^ (benzyl)), 4.49 (d, *J* = 5.8 Hz, 2H, (–CH_2_–CONH)), 3.32 (t, *J* = 6.4 Hz, 2H, (CH_2_–CO–Ph)), 2.60 (t, *J* = 6.4 Hz, 2H, (CH_2_–CH_2_–CO)) ppm, *δ*^13^CNMR(126 MHz, DMSO): 28.95, 29.21, 33.40, 39.09, 39.26, 39.34, 39.43, 39.59, 39.76, 39.93, 40.09, 61.83, 115.90, 116.07, 127.79, 127.95, 128.64, 130.42, 130.44, 131.39, 131.46, 133.12, 136.47, 141.11, 142.95, 143.01, 144.23, 161.53, 163.49, 172.22 (CONH), 199.12 (C=O).

##### 4.1.3.5 1-(2-Chlorobenzyl)-3-((4-oxo-4-phenylbutanamido)methyl)pyridin-1-ium chloride (7e)

Yield: 91%. Cream-pink powder. ^1^H NMR (400 MHz, DMSO) *δ* 9.09 (s, 1H, (H-2 pyridinium)), 9.05 (d, *J* = 6.1 Hz, 1H, (H-6 pyridinium)), 8.98 (t, *J* = 5.9 Hz, 1H, (N–H amide)), 8.58 (d, *J* = 8.0 Hz, 1H), 8.19 (dd, *J* = 8.0, 6.0 Hz, 1H), 8.00–7.93 (m, 2H), 7.64 (dd, *J* = 8.3, 6.5 Hz, 1H), 7.59–7.42 (m, 6H), 6.05 (s, 2H, CH_2_–N^+^ (benzyl)), 4.51 (d, *J* = 5.8 Hz, 2H, (–CH_2_–CONH)), 3.31 (t, *J* = 6.4 Hz, 2H, (CH_2_–CO–Ph)), 2.60 (t, *J* = 6.4 Hz, 2H, (CH_2_–CH_2_–CO)) ppm. ^13^C NMR (101 MHz, DMSO) *δ*: 29.56, 33.85, 61.72, 128.33, 128.42, 128.60, 129.19, 130.52, 131.74, 131.86, 131.95, 133.65, 133.72, 136.87, 141.61, 143.86, 144.09, 145.15, 172.75 (CONH), 199.58 (C=O) ppm.

##### 4.1.3.6 1-(3-Chlorobenzyl)-3-((4-oxo-4-phenylbutanamido)methyl)pyridin-1-ium chloride (7f)

Yield: 78%. Cream powder. ^1^H NMR (500 MHz, DMSO) *δ* 9.27–9.21 (m, 2H, (H-2,6 pyridinium)), 8.82 (t, *J* = 19.1, 5.9 Hz, 1H, (N–H amide)), 8.50 (dd, *J* = 14.9, 8.1 Hz, 1H), 8.20–8.16 (m, 1H), 8.16–8.09 (m, 1H), 7.98–7.92 (m, 2H), 7.86 (q, *J* = 2.0 Hz, 1H), 7.62–7.56 (m, 3H), 7.49 (t, *J* = 7.7 Hz, 2H), 7.35 (t, *J* = 7.9 Hz, 1H), 5.94 (s, 2H, CH_2_–N^+^ (benzyl)), 4.48 (d, *J* = 5.7 Hz, 2H, (–CH_2_–CONH)), 3.31 (t, *J* = 6.5 Hz, 2H, (CH_2_–CO–Ph)), 2.60 (t, *J* = 6.5 Hz, 2H, (CH_2_–CH_2_–CO)) ppm. ^13^C NMR (126 MHz, DMSO): 29.18, 33.44, 39.08, 39.24, 39.37, 39.41, 39.58, 39.75, 39.91, 40.08, 62.19, 122.21, 127.86, 127.97, 128.05, 128.08, 128.70, 131.29, 131.65, 131.68, 132.26, 133.21, 136.41, 136.59, 141.18, 143.00, 143.05, 143.07, 143.18, 144.28, 144.35, 172.27 (CONH), 199.16 (C=O) ppm.

##### 4.1.3.7 1-(4-Chlorobenzyl)-3-((4-oxo-4-phenylbutanamido)methyl)pyridin-1-ium chloride (7g)

Yield: 89%. Pink powder. ^1^H NMR (400 MHz, DMSO) *δ* 9.21 (t, *J* = 3.5 Hz, 2H, (H-2,6 pyridinium)), 8.95 (t, *J* = 5.9 Hz, 1H, (N–H amide)), 8.52 (d, *J* = 8.3 Hz, 1H), 8.18 (dd, *J* = 8.0, 6.1 Hz, 1H), 8.01–7.93 (m, 2H), 7.67–7.60 (m, 3H), 7.55–7.46 (m, 4H), 5.92 (s, 2H, CH_2_–N^+^ (benzyl)), 4.49 (d, *J* = 5.8 Hz, 2H, (–CH_2_–CONH)), 3.32 (t, *J* = 6.4 Hz, 2H, (CH_2_–CO–Ph)), 2.62 (t, *J* = 6.4 Hz, 2H, (CH_2_–CH_2_–CO)) ppm. ^13^C NMR (101 MHz, DMSO) *δ*: 29.56, 33.83, 39.36, 39.57, 39.78, 39.83, 39.98, 40.19, 40.40, 40.61, 62.83, 128.34, 128.53, 129.19, 129.63, 131.30, 133.66, 133.72, 134.65, 136.90, 141.71, 143.47, 143.68, 144.74, 172.75 (CONH), 199.66 (C=O) ppm, HRMS (ESI) (*m*/*z*): found for C_23_H_22_ClN_2_O_2_^+^ [M + H]^+^ 393.0533.

##### 4.1.3.8 1-(4-Bromoobenzyl)-3-((4-oxo-4-phenylbutanamido)methyl)pyridin-1-ium bromide (7h)

Beige solid; yield: 75%, ^1^H NMR (500 MHz, DMSO) *δ* 9.24–9.18 (m, 2H, (H-2,6 pyridinium)), 8.94 (t, *J* = 5.9 Hz, 1H, (N–H amide)), 8.54–8.49 (m, 1H), 8.16 (dd, *J* = 8.1, 6.0 Hz, 1H), 7.98–7.92 (m, 2H), 7.65–7.58 (m, 3H), 7.53–7.41 (m, 4H), 5.93 (s, 2H, CH_2_–N^+^ (benzyl)), 4.48 (d, *J* = 5.8 Hz, 2H, (–CH_2_–CONH)), 3.30 (t, *J* = 6.5 Hz, 2H, (CH_2_–CO–Ph)), 2.61 (t, *J* = 6.4 Hz, 2H, (CH_2_–CH_2_–CO)) ppm, *δ*^13^CNMR(101 MHz, DMSO): 29.50, 33.77, 39.34, 39.54, 39.75, 39.96, 40.17, 40.38, 40.59, 44.80, 62.70, 124.47, 124.50, 124.60, 128.31, 128.67, 129.18, 129.51, 130.37, 130.54, 130.59, 133.73, 136.87, 140.19, 141.68, 141.83, 143.76, 144.05, 145.02, 148.32, 172.79 (CONH), 199.66 (C=O) ppm.

##### 4.1.3.9 3-((4-Oxo-4-phenylbutanamido)methyl)-1-(4-(trifluoromethyl)benzyl)pyridin-1-ium bromide (7i)

Beige solid; yield: 76%, ^1^H NMR (500 MHz, DMSO) *δ* 9.23–9.18 (m, 2H, (H-2,6 pyridinium)), 8.85 (t, *J* = 5.9 Hz, 1H, (N–H amide)), 8.51 (d, *J* = 8.1 Hz, 1H), 8.17 (dd, *J* = 8.1, 6.0 Hz, 1H), 8.03 (s, 1H), 7.98–7.93 (m, 2H), 7.85 (d, *J* = 7.8 Hz, 1H), 7.77 (d, *J* = 7.8 Hz, 1H), 7.64 (dt, *J* = 12.6, 7.6 Hz, 2H), 7.51 (t, *J* = 7.6 Hz, 2H), 5.99 (s, 2H, CH_2_–N^+^ (benzyl)), 4.48 (d, *J* = 5.8 Hz, 2H, (–CH_2_–CONH)), 3.31 (t, *J* = 6.5 Hz, 2H, (CH_2_–CO–Ph)), 2.59 (t, *J* = 6.4 Hz, 2H, (CH_2_–CH_2_–CO)) ppm. ^13^C NMR (101 MHz, DMSO) *δ* 29.5, 33.77, 39.34, 39.44, 39.54, 39.75, 39.96, 40.17, 40.38, 40.59, 45.9, 62.7, 114.58, 124.47, 124.5, 124.6, 128.31, 128.67, 129.18, 129.51, 130.37, 130.54, 130.59, 133.73, 136.87, 140.19, 141.68, 141.83, 143.76, 144.05, 145.02, 148.32, 172.79 (CONH), 199.66 (C=O) ppm.

##### 4.1.3.10 1-(4-Nitrobenzyl)-3-((4-oxo-4-phenylbutanamido)methyl)pyridin-1-ium bromide (7j)

Dark pink solid; yield: 87%, ^1^H NMR (400 MHz, DMSO) *δ* 9.19–9.15 (m, 2H, (H-2,6 pyridinium)), 8.81 (t, *J* = 5.9 Hz, 1H, (N–H amide)), 8.53 (d, *J* = 8.0 Hz, 1H), 8.20 (dd, *J* = 8.0, 6.1 Hz, 1H), 8.01–7.94 (m, 2H), 7.80 (d, *J* = 8.2 Hz, 2H), 7.73 (d, *J* = 8.1 Hz, 2H), 7.68–7.62 (m, 1H), 7.53 (t, *J* = 7.7 Hz, 2H), 6.01 (s, 2H, CH_2_–N^+^ (benzyl)), 4.50 (d, *J* = 5.8 Hz, 2H, (–CH_2_–CONH)), 3.32 (t, *J* = 6.4 Hz, 2H, (CH_2_–CO–Ph)), 2.61 (t, *J* = 6.4 Hz, 2H, (CH_2_–CH_2_–CO)). ^13^C NMR (101 MHz, DMSO) *δ* (101 MHz, DMSO): 29.50, 33.78, 39.34, 39.55, 39.76, 39.86, 39.97, 40.17, 40.38, 40.59, 63.00, 123.03, 125.74, 126.44, 126.48, 126.52, 126.56, 128.32, 128.63, 129.18, 129.93, 130.23, 133.73, 136.88, 139.19, 141.79, 143.65, 143.94, 144.90, 172.77 (CONH), 199.67 (C=O) ppm.

##### 4.1.3.11 1-(4-Methoxybenzyl)-3-((4-oxo-4-phenylbutanamido)methyl)pyridin-1-ium bromide (7k)

Purple solid; yield: 75%, ^1^H NMR (500 MHz, DMSO) *δ* 9.10 (d, *J* = 8.2 Hz, 2H, (H-2,6 pyridinium)), 8.73 (t, *J* = 5.9 Hz, 1H, (N–H amide)), 8.48 (d, *J* = 8.1 Hz, 1H), 8.14 (dd, *J* = 8.1, 5.9 Hz, 1H), 7.97 (d, *J* = 7.7 Hz, 2H), 7.64 (t, *J* = 7.3 Hz, 1H), 7.52 (t, *J* = 7.6 Hz, 2H), 7.32 (t, *J* = 7.9 Hz, 1H), 7.14 (t, *J* = 2.1 Hz, 1H), 7.07 (d, *J* = 7.6 Hz, 1H), 6.97 (dd, *J* = 8.3, 2.6 Hz, 1H), 5.80 (s, 2H, CH_2_–N^+^ (benzyl)), 4.48 (d, *J* = 5.8 Hz, 2H, (–CH_2_–CONH)), 3.73 (s, 3H, (–OCH_3_)), 3.31 (t, 2H, (CH_2_–CO–Ph)), 2.58 (t, *J* = 6.4 Hz, 2H, (CH_2_–CH_2_–CO)). *δ*^13^CNMR(126 MHz, DMSO): 13.48, 19.21, 23.07, 29.06, 33.32, 39.04, 39.20, 39.37, 39.54, 39.70, 39.87, 40.04, 55.22, 57.52, 57.55, 63.42, 114.52, 114.75, 120.65, 127.85, 128.02, 128.72, 130.42, 133.26, 135.53, 136.43, 141.23, 142.81, 143.17, 144.20, 159.66, 172.28 (CONH), 199.21 (C=O) ppm.

##### 4.1.3.12 1-(4-Methylbenzyl)-3-((4-oxo-4-phenylbutanamido)methyl)pyridin-1-ium chloride (7l)

Cream solid; yield: 80%, ^1^H NMR (500 MHz, DMSO) *δ* 9.14 (d, *J* = 6.9 Hz, 2H, (H-2,6 pyridinium)), 8.80 (t, *J* = 5.9 Hz, 1H, (N–H amide)), 8.49 (d, *J* = 8.0 Hz, 1H), 8.18–8.12 (m, 1H), 7.96 (d, *J* = 7.7 Hz, 2H), 7.62 (t, *J* = 7.4 Hz, 1H), 7.51 (t, *J* = 7.6 Hz, 2H), 7.45 (d, *J* = 7.8 Hz, 2H), 7.20 (d, *J* = 7.7 Hz, 2H), 5.84 (s, 2H, CH_2_–N^+^ (benzyl)), 4.48 (d, *J* = 5.8 Hz, 2H, (–CH_2_–CONH)), 3.31 (t, *J* = 6.4 Hz, 2H, (CH_2_–CO–Ph)), 2.60 (t, *J* = 6.4 Hz, 2H, (CH_2_–CH_2_–CO)), 2.25 (s, 3H) ppm, *δ*^13^CNMR (126 MHz, DMSO): 20.70, 29.10, 33.35, 39.02, 39.19, 39.35, 39.52, 39.69, 39.78, 39.85, 39.95, 40.02, 63.13, 91.62, 125.77, 127.49, 127.81, 127.92, 128.16, 128.67, 128.76, 128.86, 129.65, 131.27, 133.17, 136.46, 138.91, 139.68, 141.15, 142.99, 144.10, 144.50, 172.20, 174.73 (CONH), 199.13 (C=O) ppm.

##### 4.1.3.13 1-(2-Methylbenzyl)-3-((4-oxo-4-phenylbutanamido)methyl)pyridin-1-ium chloride (7m)

Yield: 74%. Dark cream powder. ^1^H NMR (400 MHz, DMSO) *δ* 9.00 (s, 1H, (H-6 pyridinium)), 8.97 (d, *J* = 6.1 Hz, 1H), 8.89 (t, *J* = 5.9 Hz, 1H, (N–H amide)), 8.55 (d, *J* = 8.0 Hz, 1H), 8.21–8.15 (m, 1H), 7.99–7.94 (m, 2H), 7.68–7.62 (m, 1H), 7.53 (t, *J* = 7.6 Hz, 2H), 7.35–7.30 (m, 2H), 7.26–7.22 (m, 1H), 7.11 (d, *J* = 7.6 Hz, 1H), 5.95 (s, 2H, CH_2_–N^+^ (benzyl)), 4.50 (d, *J* = 5.8 Hz, 2H, (–CH_2_–CONH)), 3.31 (t, *J* = 6.4 Hz, 2H, (CH_2_–CO–Ph)), 2.59 (t, *J* = 6.4 Hz, 2H, (CH_2_–CH_2_–CO)), 2.30 (s, 3H) ppm. ^13^C NMR (101 MHz, DMSO) *δ* 19.31, 29.53, 33.81, 39.85, 62.07, 127.16, 128.33, 128.48, 129.2, 129.33, 129.81, 131.37, 132.77, 133.73, 136.88, 137.34, 141.62, 143.61, 143.88, 144.85, 172.75 (CONH), 199.58 (C=O) ppm, HRMS (ESI) (*m*/*z*): found for C_24_H_25_N_2_O_2_^+^ [M + H]^+^ 373.1218.

#### 4.1.4 1-Methyl-3-((4-oxo-4-phenylbutanamido)methyl)pyridin-1-ium iodide (7n)

Yield: 87%, beige color, ^1^H NMR (500 MHz, DMSO) *δ* 8.88 (dd, *J* = 3.5, 1.8 Hz, 2H, (H-2,6 pyridinium)), 8.73 (t, *J* = 5.9 Hz, 1H, (N–H amide)), 8.43 (d, *J* = 8.1 Hz, 1H), 8.13–8.07 (m, 1H), 8.00–7.95 (m, 2H), 7.67–7.60 (m, 1H), 7.52 (t, *J* = 7.7 Hz, 2H), 4.46 (d, *J* = 5.9 Hz, 2H, (–CH_2_–CONH)), 4.36 (s, 3H, (CH_3_–N^+^)), 3.32 (t, *J* = 6.4 Hz, 2H, (CH_2_–CO–Ph)), 2.60 (t, *J* = 6.4 Hz, 2H, (CH_2_–CH_2_–CO)) ppm, ^13^C NMR (126 MHz, DMSO): 29.10, 30.11, 33.37, 39.06, 39.23, 39.39, 39.56, 39.65, 39.73, 39.82, 39.89, 39.99, 40.06, 40.16, 48.07, 127.27, 127.87, 128.74, 133.26, 136.42, 140.33, 143.26, 143.59, 143.91, 172.26 (CONH), 199.17 (C=O) ppm, HRMS (ESI) (*m*/*z*): found for C_17_H_19_N_2_O_2_^+^ [M + H]^+^ 283.0973.

### 4.2 Screening of AChE and BChE inhibitory activity

Cholinesterase inhibitory activities of all derivatives were assessed using the modified Ellman method using butyrylcholinesterase (BChE, E.C. 3.1.1.8, from horse serum), and acetylcholinesterase (AChE, E.C. 3.1.1.7, Type V-S, lyophilized powder)^17^

### 4.3 Enzyme kinetic studies

The mode of inhibition for the most active compound, 7b, against AChE was investigated using acetylthiocholine as a substrate at concentrations ranging from 0.1 to 1 mM. A Lineweaver–Burk plot was generated, plotting the reciprocal of the substrate concentration (1/[S]) against the reciprocal of the enzyme rate (1/*V*) across various inhibitor concentrations. This plot was used to identify the type of inhibition and determine the Michaelis–Menten constant (*K*_m_) value.^18,19^

### 4.4 Cell viability

SH-SY5Y cells were cultured in DMEM/F12 medium supplemented with 15% FBS, penicillin, and streptomycin, and maintained at 37 °C with 5% CO_2_ cell viability was evaluated using the MTT assay. Cells were seeded in 96-well plates, treated with various concentrations of test compounds for 72 hours, and incubated with MTT reagent (0.5 mg mL^−1^) for 2 hours. The resulting formazan crystals were dissolved in DMSO, and absorbance was measured at 540 nm. Untreated cells served as the 100% viability control.^20,21^

### 4.5 Neuroprotectivity assay on SH-SY5Y

SH-SY5Y cells were cultured and incubated for 24 hours. Subsequently, 100 µM H_2_O_2_ was added to each well of a 96-well plate, except for the healthy control group. After 4 hours of H_2_O_2_ exposure, various concentrations of the test compound were added. Following a 72 hour incubation period, cell viability was assessed using the MTT assay.^22^

### 4.6 Molecular docking

The molecular docking of compound 7b was performed using the Schrödinger Suites Maestro molecular modeling platform. The X-ray crystallographic structures of AChE and BChE used for this purpose were obtained from the RCSB Protein Data Bank with PDB IDs 4EY7 and 4BDS.^23^

### 4.7 MD simulations

The starting model for the molecular dynamics simulations was obtained by imposing induced fit docking to AChE. The MD simulations themselves were conducted using Desmond v5.3 of Schrodinger's Suite, Maestro, following previously reported procedures.^24^

### 4.8 Prediction of pharmacokinetic properties of 7c and donepezil

The physicochemical and biological absorption, distribution, metabolism, excretion, and toxicity (ADMET) properties of the selected compounds were studied. These predictions were generated using reliable tools, including PKCSM (https://biosig.lab.uq.edu.au/pkcsm/) and ADMETmesh https://admetlab3.scbdd.com/and https://admetmesh.scbdd.com/.

## Author contributions

M. E. synthesized compounds and contributed to the characterization of compounds. A. I. performed *in silico* and biological studies. S. J. and M. M. supervised the synthetic part of the study. All authors read and approved the final version of the article.

## Conflicts of interest

There are no conflicts to declare.

## Supplementary Material

RA-016-D5RA06941F-s001

## Data Availability

The datasets generated and/or analyzed during the current study are available in the Worldwide ProteinData Bank with the PDB IDs of the 4EY7 and 4BDS repository. Supplementary information: ^1^H NMR and ^13^C NMR spectra, is available. See DOI: https://doi.org/10.1039/d5ra06941f.

## References

[cit1] Tenchov R., Sasso J. M., Zhou Q. A. (2024). ACS Chem. Neurosci..

[cit2] Breijyeh Z., Karaman R. (2020). Molecules.

[cit3] Yazdani M., Edraki N., Badri R., Khoshneviszadeh M., Iraji A., Firuzi O. (2020). Mol. Diversity.

[cit4] Haghighijoo Z., Akrami S., Saeedi M., Zonouzi A., Iraji A., Larijani B., Fakherzadeh H., Sharifi F., Arzaghi S. M., Mahdavi M., Edraki N. (2020). Bioorg. Chem..

[cit5] Better M. A. (2023). Alzheimers Dement.

[cit6] Oliyaei N., Moosavi-Nasab M., Tanideh N., Iraji A. (2023). Brain Res. Bull..

[cit7] Pourtaher H., Hasaninejad A., Iraji A. (2022). Sci. Rep..

[cit8] Pourtaher H., Hasaninejad A., Zare S., Tanideh N., Iraji A. (2023). Sci. Rep..

[cit9] Zhang J., Zhang Y., Wang J., Xia Y., Zhang J., Chen L. (2024). Signal Transduction Targeted Ther..

[cit10] Xing H., Yue S., Qin R., Du X. (2025). Int. J. Mol. Sci..

[cit11] Kamath A. P., Nayak P. G., John J., Mutalik S., Balaraman A. K., Krishnadas N. (2024). Neuropharmacology.

[cit12] Lan J.-S., Zhang T., Liu Y., Yang J., Xie S.-S., Liu J., Miao Z.-Y., Ding Y. (2017). Eur. J. Med. Chem..

[cit13] Abedinifar F., Farnia S. M. F., Mahdavi M., Nadri H., Moradi A., Ghasemi J. B., Küçükkılınç T. T., Firoozpour L., Foroumadi A. (2018). Bioorg. Chem..

[cit14] Lan J.-S., Hou J.-W., Liu Y., Ding Y., Zhang Y., Li L., Zhang T. (2017). J. Enzyme Inhib. Med. Chem..

[cit15] Ghotbi G., Mahdavi M., Najafi Z., Moghadam F. H., Hamzeh-Mivehroud M., Davaran S., Dastmalchi S. (2020). Bioorg. Chem..

[cit16] Vitorović-Todorović M. D., Juranić I. O., Mandić L. M., Drakulić B. J. (2010). Bioorg. Med. Chem..

[cit17] Pourtaher H., Hasaninejad A., Iraji A. (2022). Sci. Rep..

[cit18] Iraji A., Nikfar P., Nazari Montazer M., Karimi M., Edraki N., Saeedi M., Mirfazli S. S. (2024). Sci. Rep..

[cit19] Sadeghian B., Sakhteman A., Faghih Z., Nadri H., Edraki N., Iraji A., Sadeghian I., Rezaei Z. (2020). J. Mol. Struct..

[cit20] Noori M., Dastyafteh N., Safapoor S., Khalili Ghomi M., Tanideh R., Zomorodian K., Hamedifar H., Dara M., Zare S., Irajie C., Javanshir S., Rastegar H., Panahi N., Larijani B., Mahdavi M., Hajimiri M. H., Iraji A. (2023). Int. J. Biol. Macromol..

[cit21] Edraki N., Iraji A., Firuzi O., Fattahi Y., Mahdavi M., Foroumadi A., Khoshneviszadeh M., Shafiee A., Miri R. (2016). J. Iran. Chem. Soc..

[cit22] Iraji A., Sharghei-Boroujeni D., Aliabadi A., Dara M., Hashempur M. H., Hariri R., Khassaki D. A. M., Saeedi M., Akbarzadeh T. (2025). J. Mol. Struct..

[cit23] Pourtaher H., Mohammadi Y., Hasaninejad A., Iraji A. (2024). RSC Med. Chem..

[cit24] Iraji A., Hariri R., Hashempur M. H., Ghasemi M., Pourtaher H., Saeedi M., Akbarzadeh T. (2025). BMC Chem..

