# Supplementary material for: Novel cinnamic acid-based N-benzyl pyridinium analogs: potent dual cholinesterase inhibitors with neuroprotective properties for Alzheimer's disease
Source: RSC Adv. 2026 Feb 16;16(10):9293–306. doi: 10.1039/d5ra06941f (PMC12908697; doi:10.1039/d5ra06941f)
Supplement: RA-016-D5RA06941F-s001 [file RA-016-D5RA06941F-s001.pdf]

Supplementary Information (SI) for RSC Advances.
This journal is © The Royal Society of Chemistry 2026

Supporting information

Novel cinnamic acid-based N-benzyl pyridinium analogs: potent dual cholinesterase inhibitors with neuroprotective properties for Alzheimer's disease

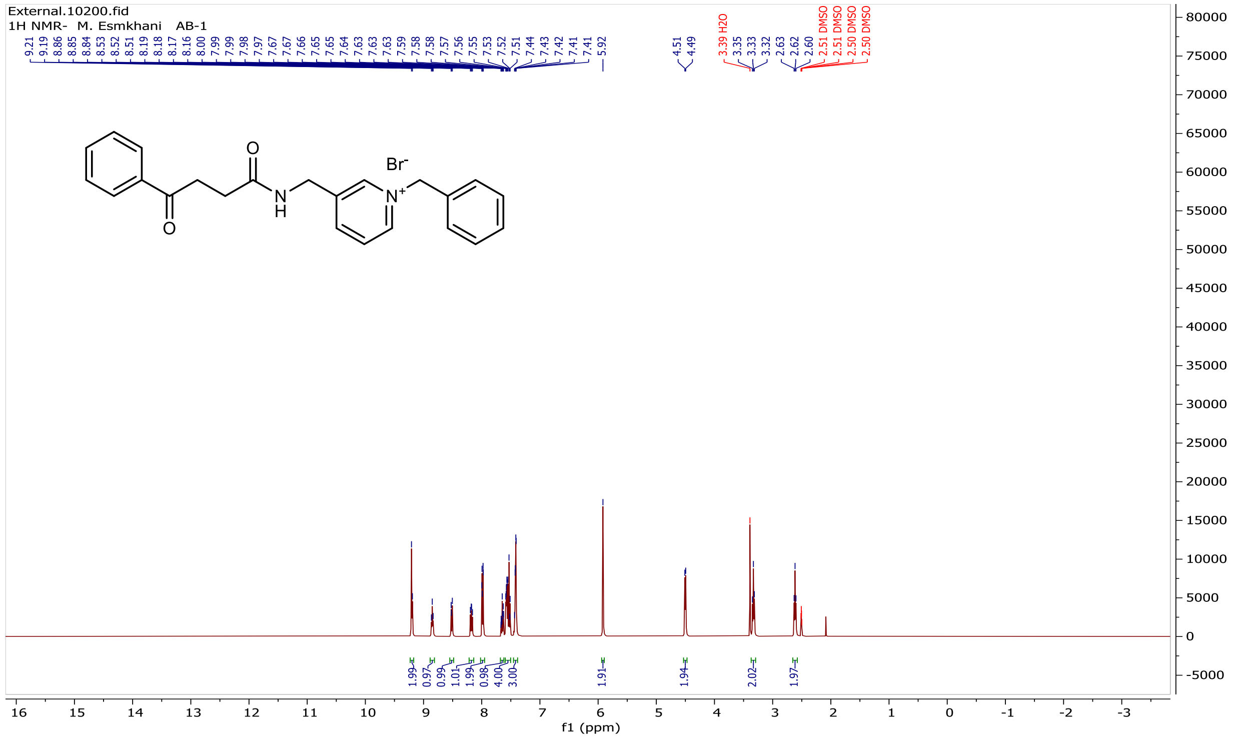

Figure S 1. ^{1}HNMR spectra of compound 7a.

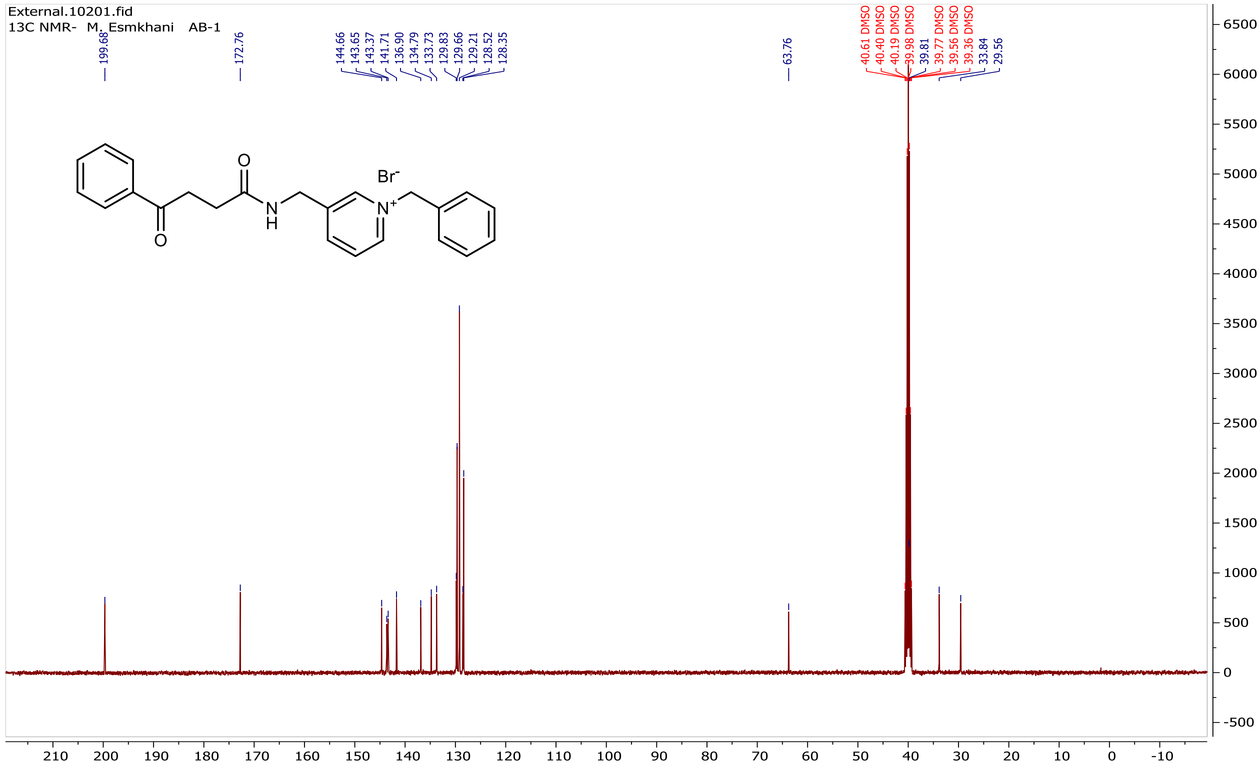

Figure S 2. ^{13}CNMR spectra of compound 7a.

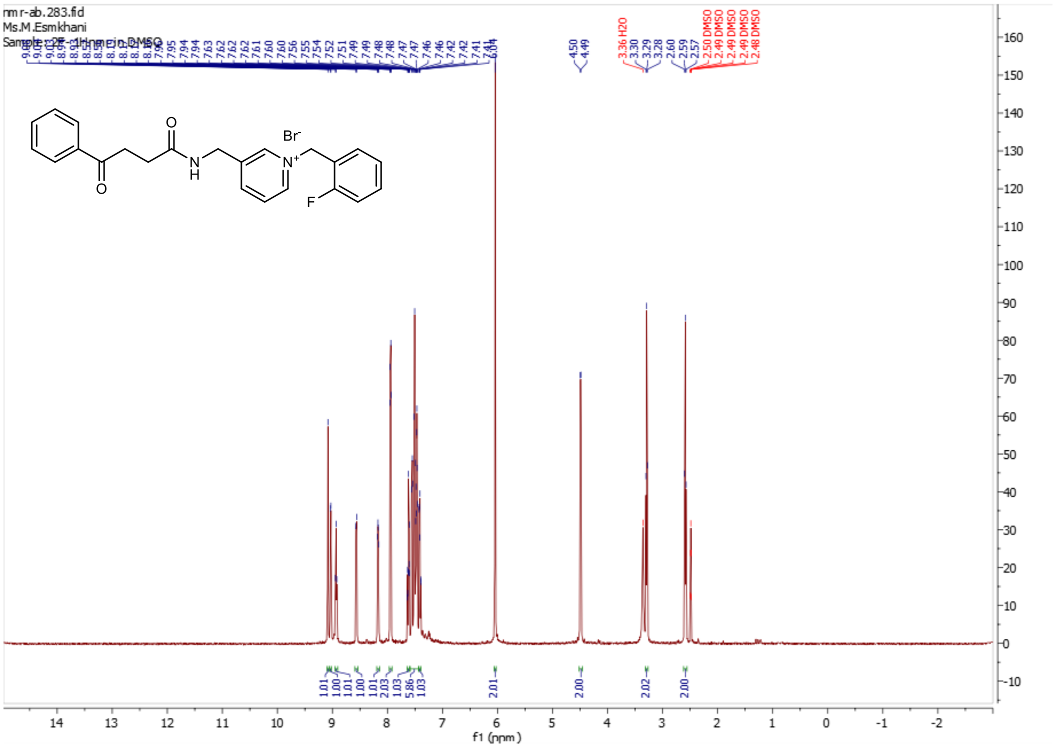

Figure S 3. ^{1}HNMR spectra of compound 7b.

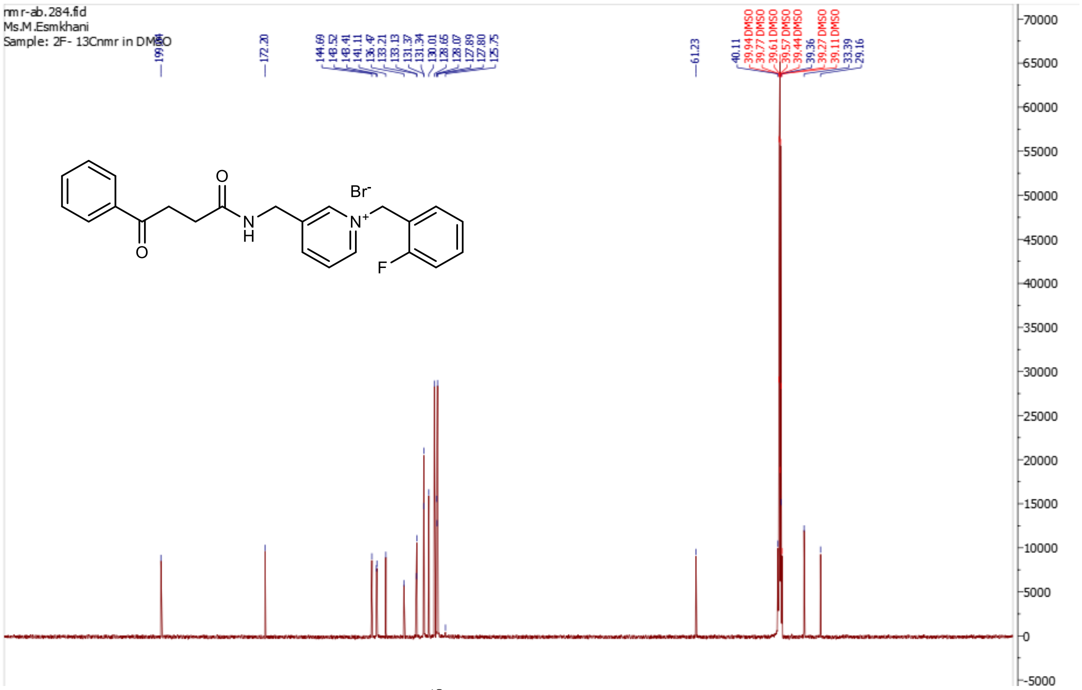

Figure S 4.^{13}CNMR spectra of compound 7b.

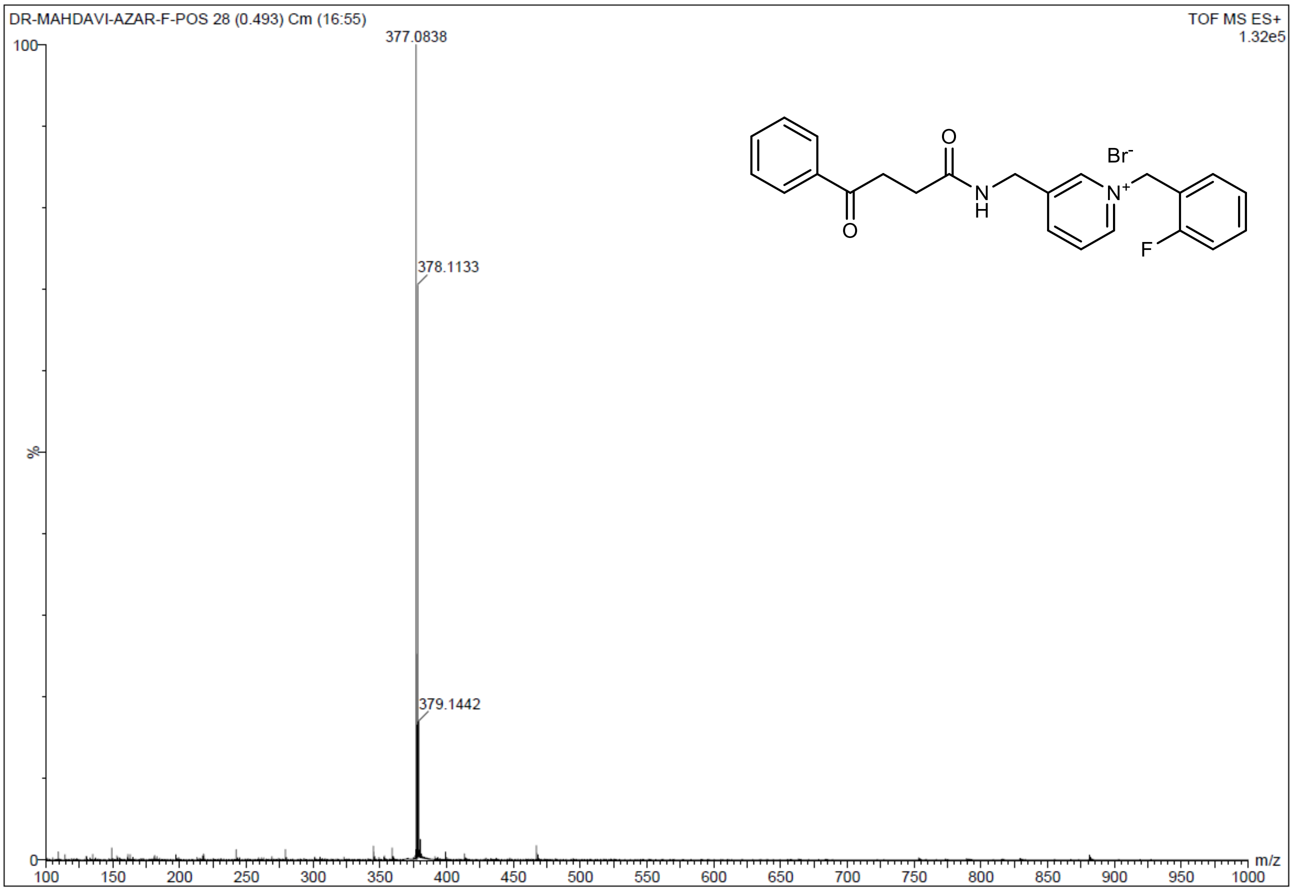

Figure S 5. HRMS spectra of compound 7b.

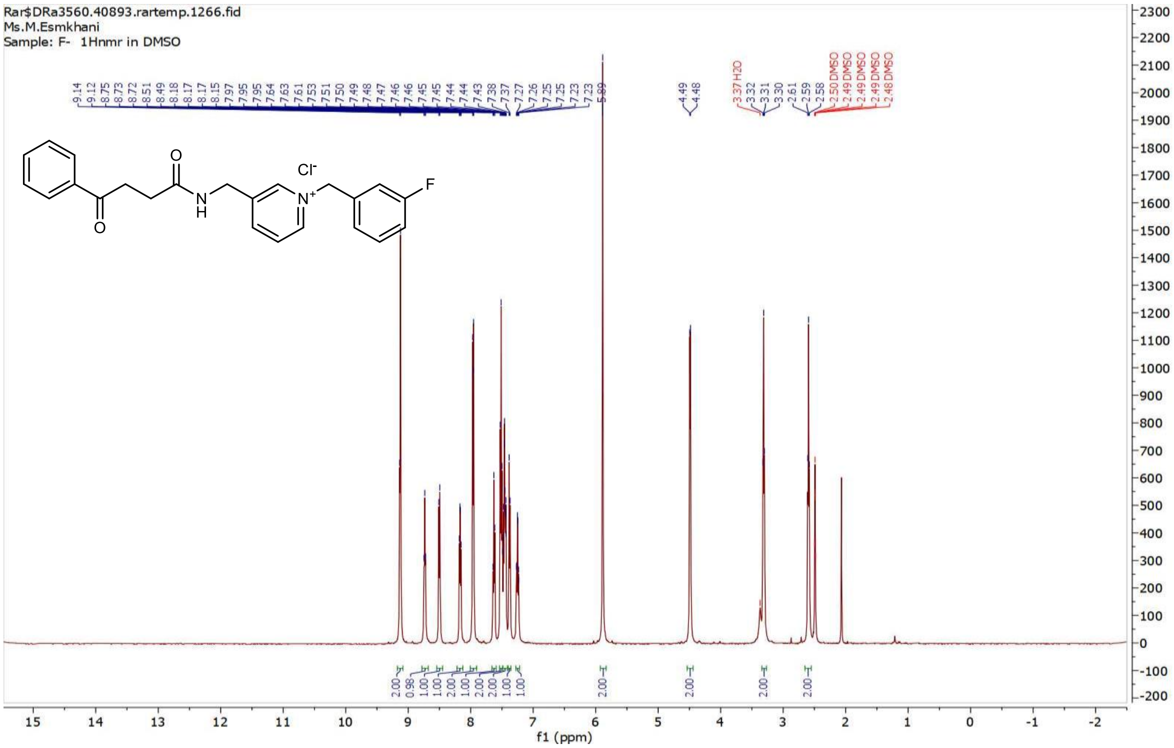

Figure S 6. ^{1}HNMR spectra of compound 7c.

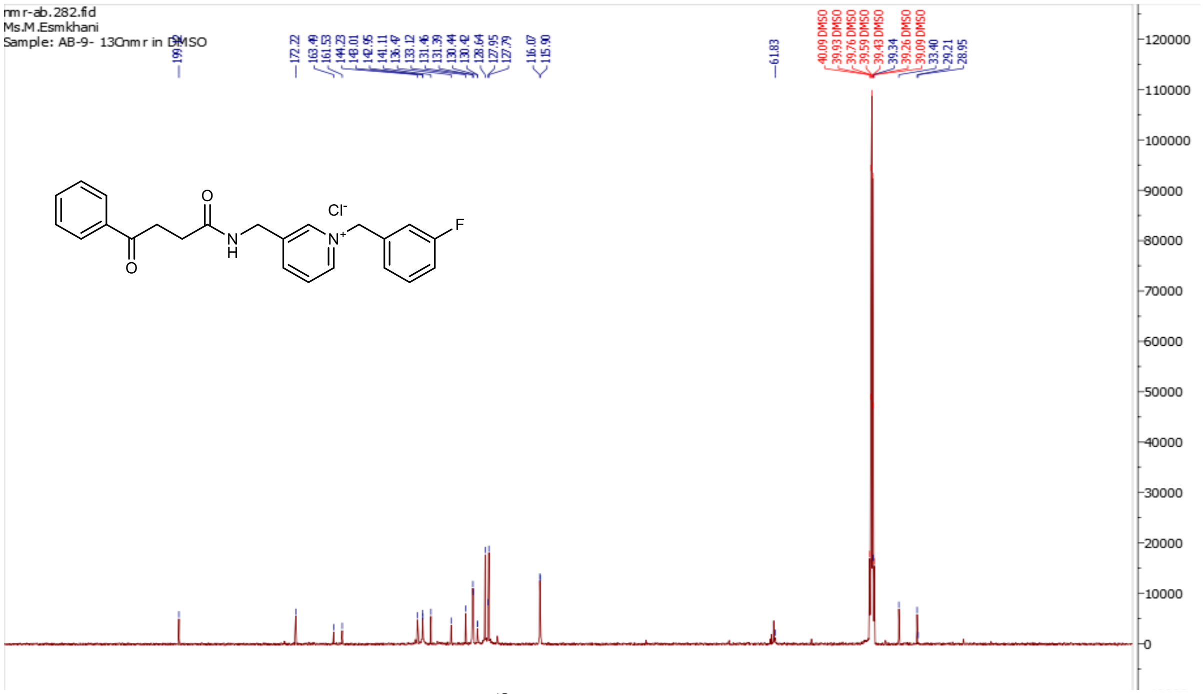

Figure S 7. ^{13}CNMR spectra of compound 7c.

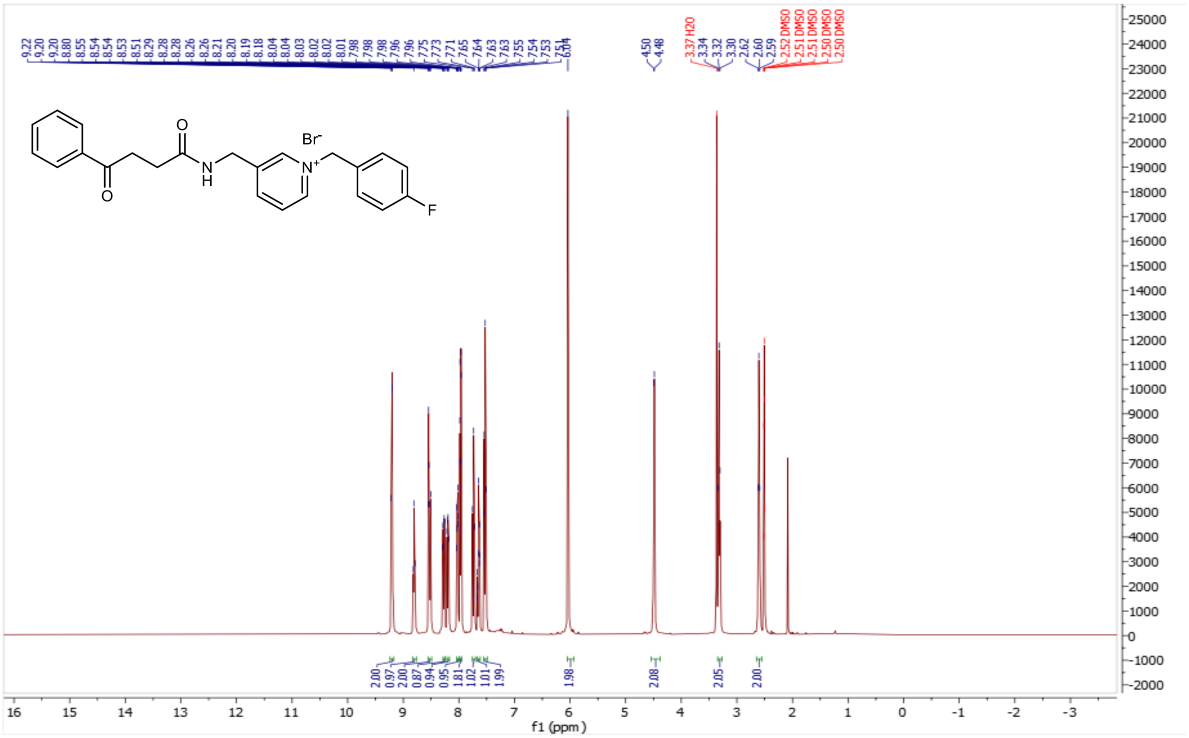

Figure S 8. ^{1}HNMR spectra of compound 7d.

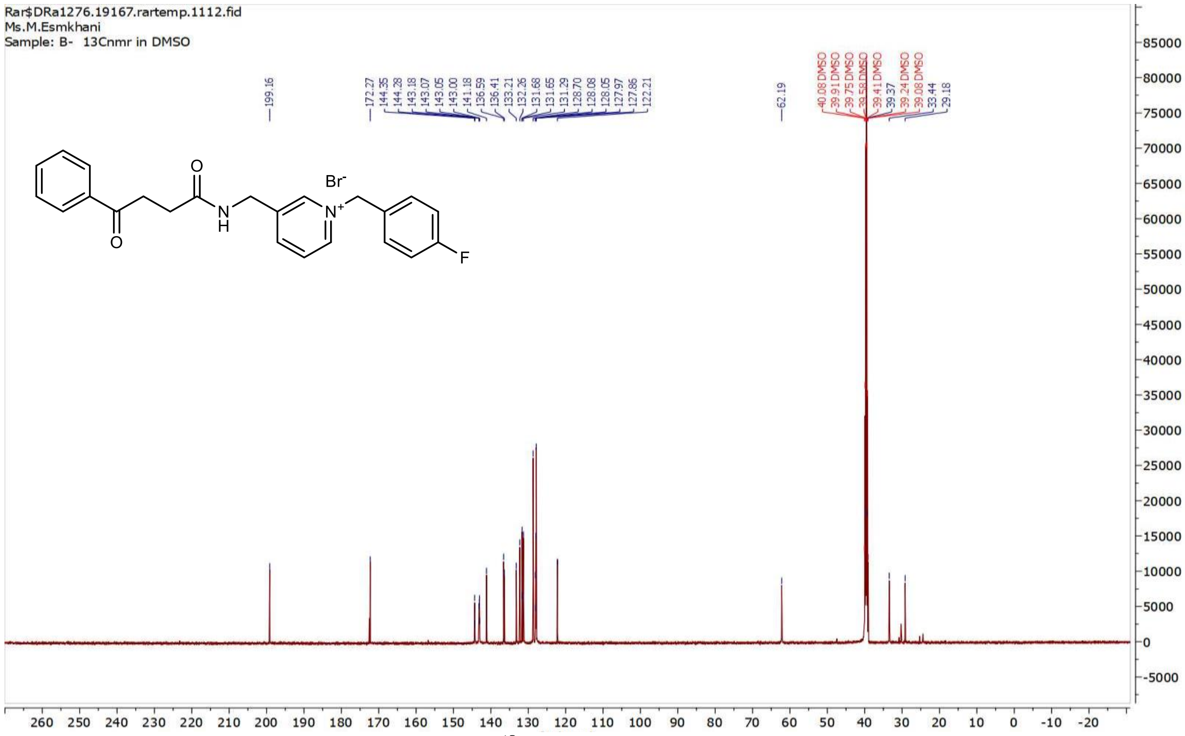

Figure S 9. ^{13}CNMR spectra of compound 7d

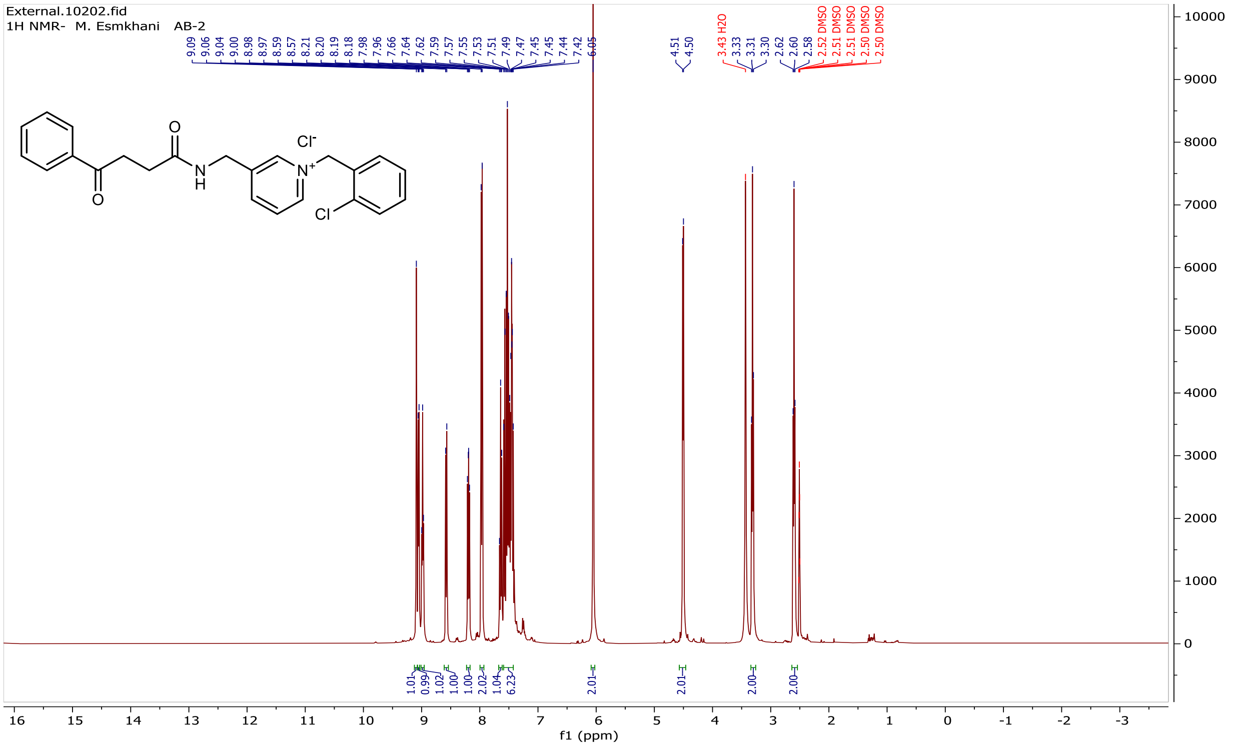

Figure S 10. ^{1}HNMR spectra of compound 7e.

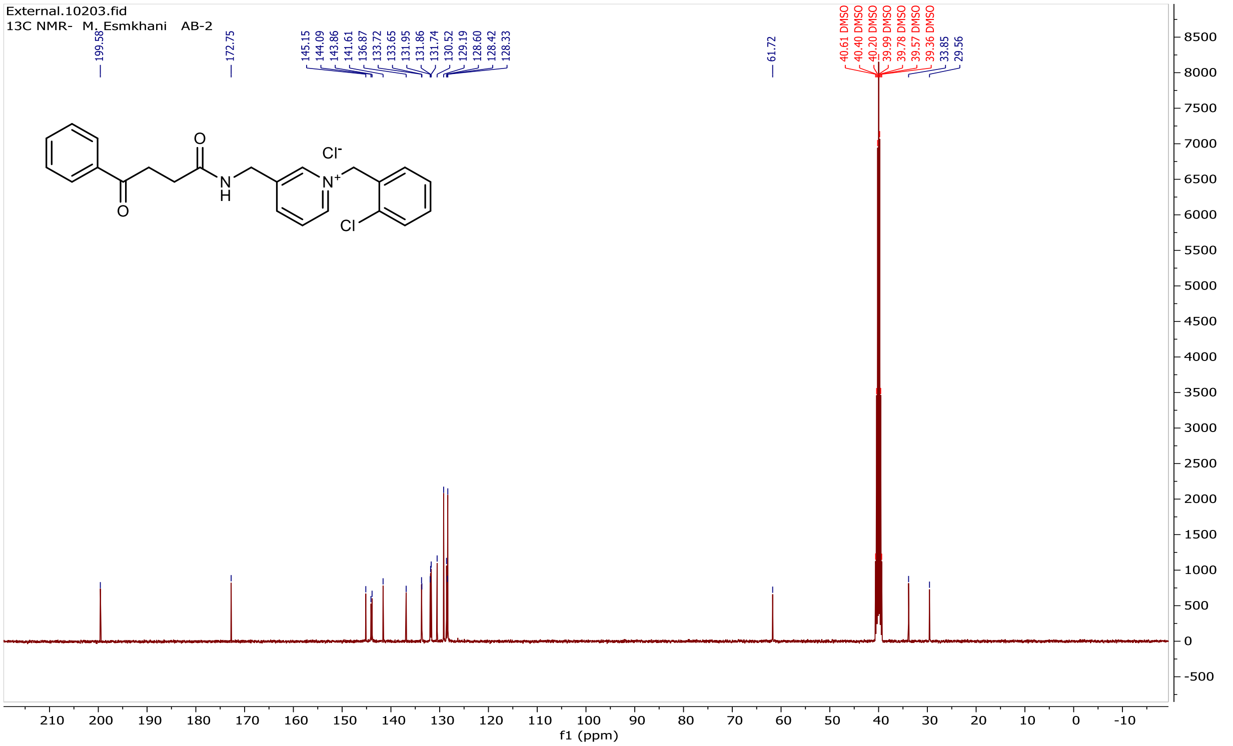

Figure S 11. ^{13}CNMR spectra of compound 7e.

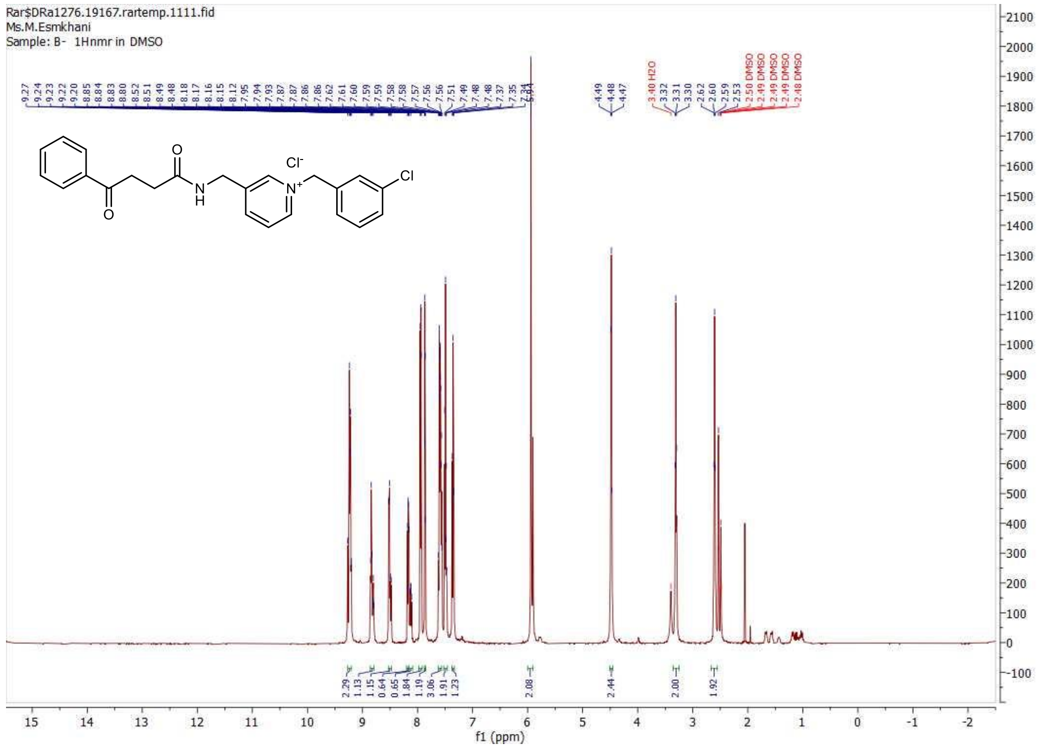

Figure S 12. ^{1}HNMR spectra of compound 7f.

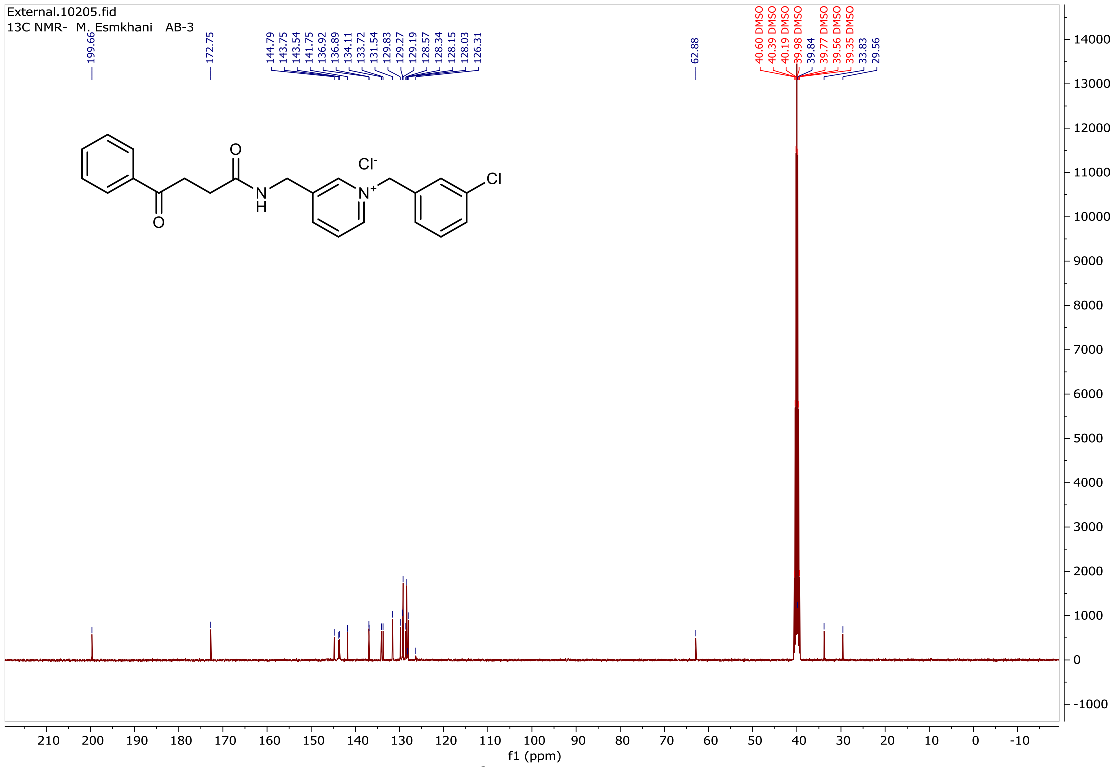

Figure S 13. ^{13}CNMR spectra of compound 7f.

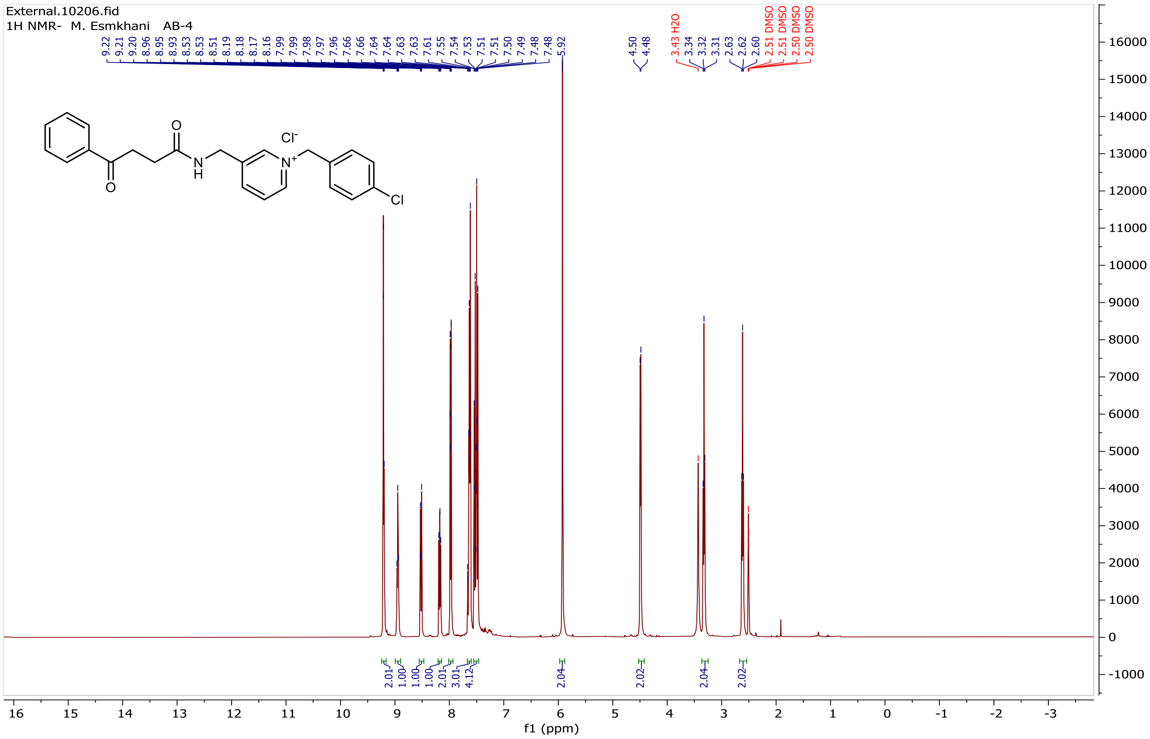

Figure S 14. ^{1}HNMR spectra of compound 7g.

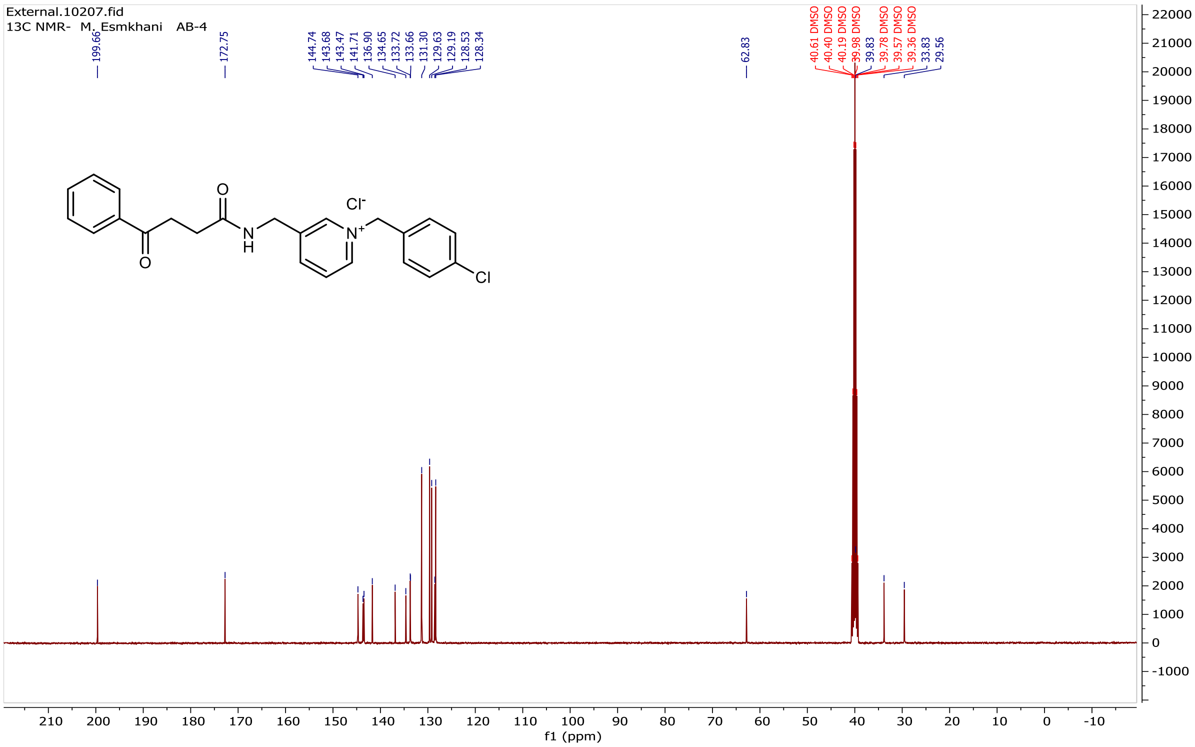

Figure S 15.^{13}CNMR spectra of compound 7g.

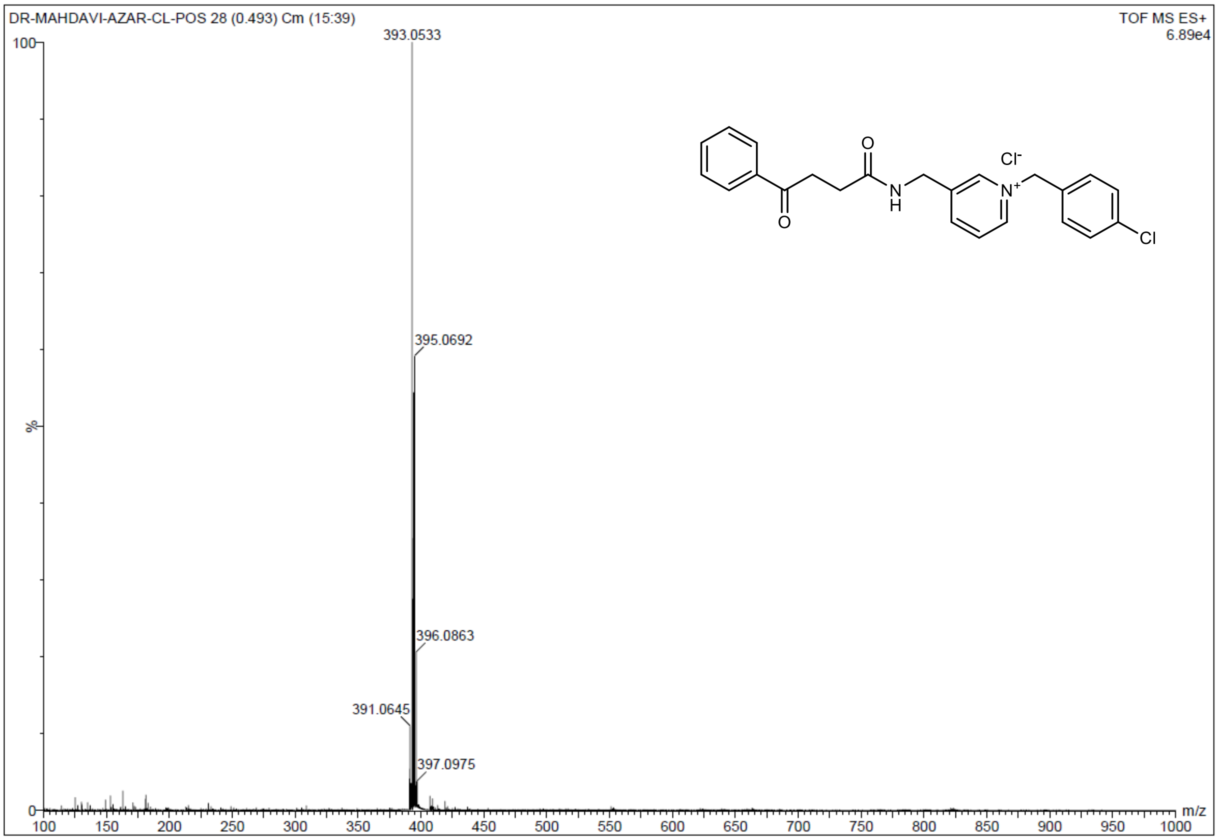

Figure S 16. HRMS spectra of compound 7g.

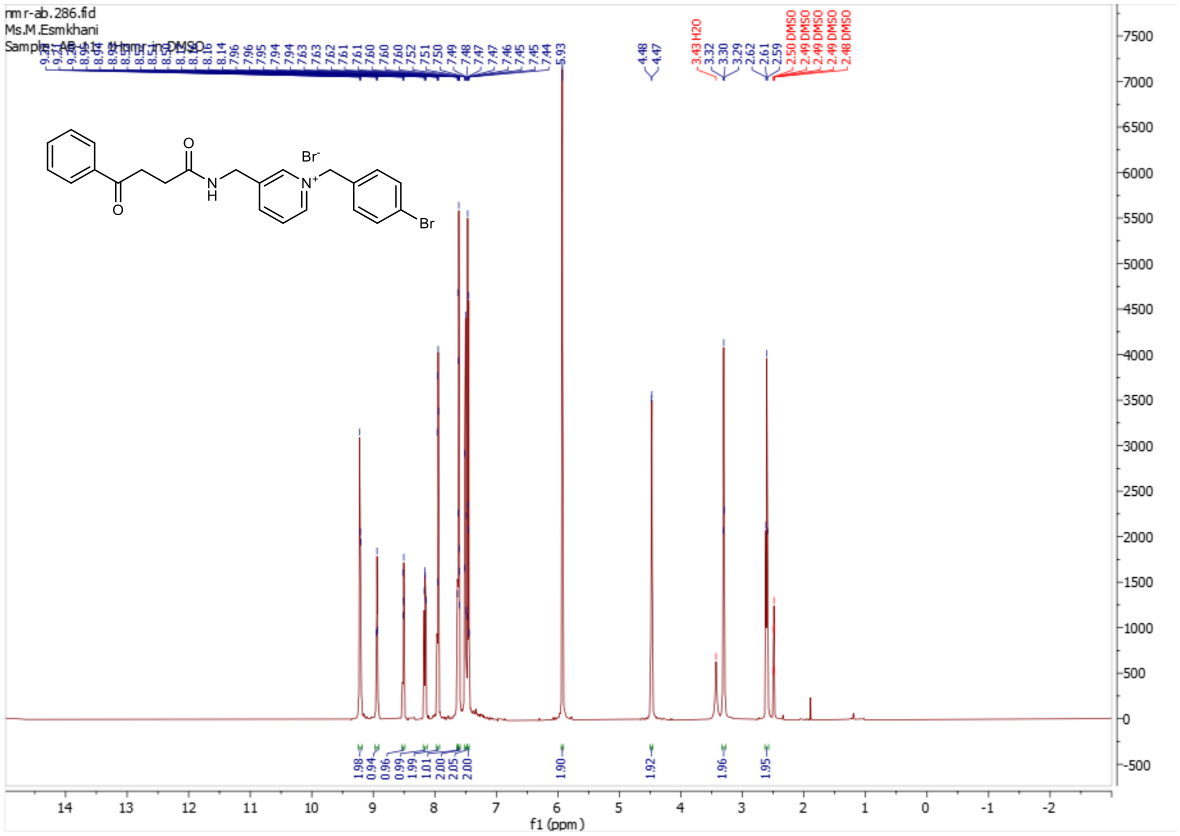

Figure S 17. ^{1}HNMR spectra of compound 7h.

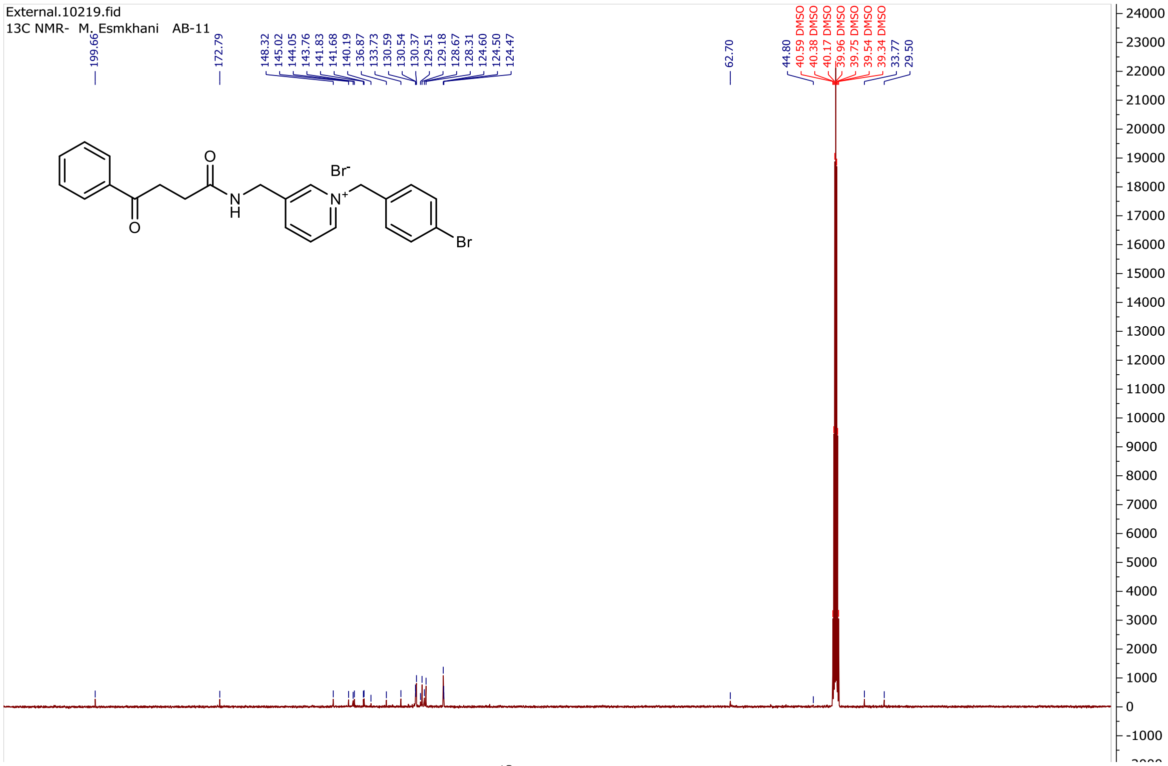

Figure S 18. ^{13}CNMR spectra of compound 7h

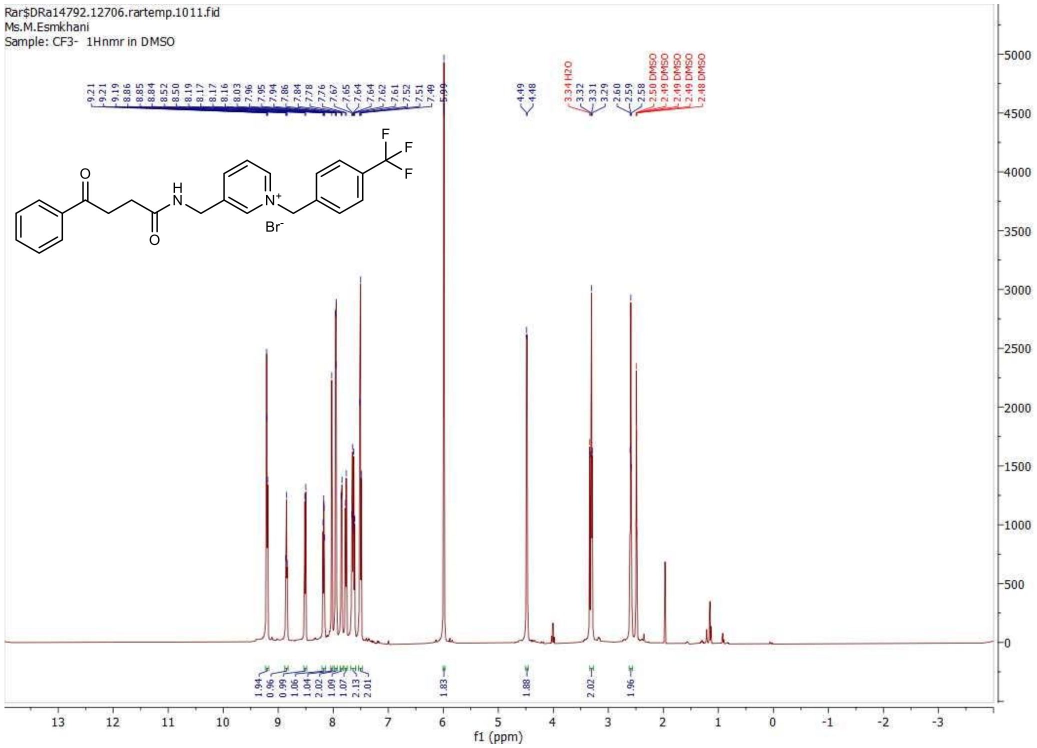

Figure S 19. ^{1}HNMR spectra of compound 7i.

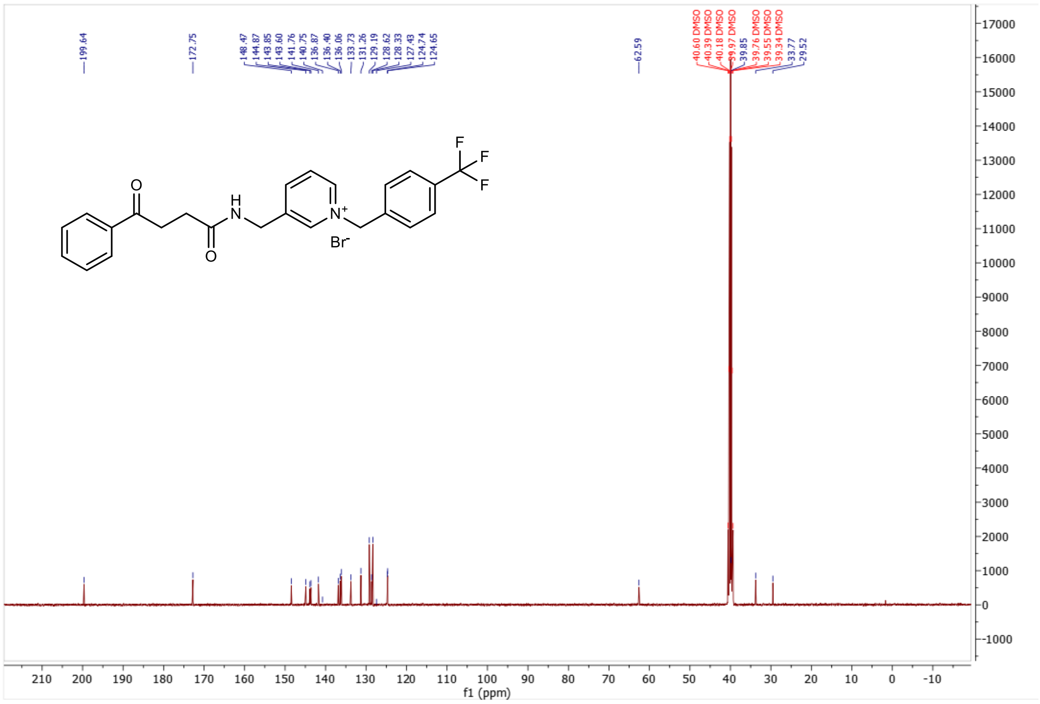

Figure S 20. ^{1}HNMR spectra of compound 7i.

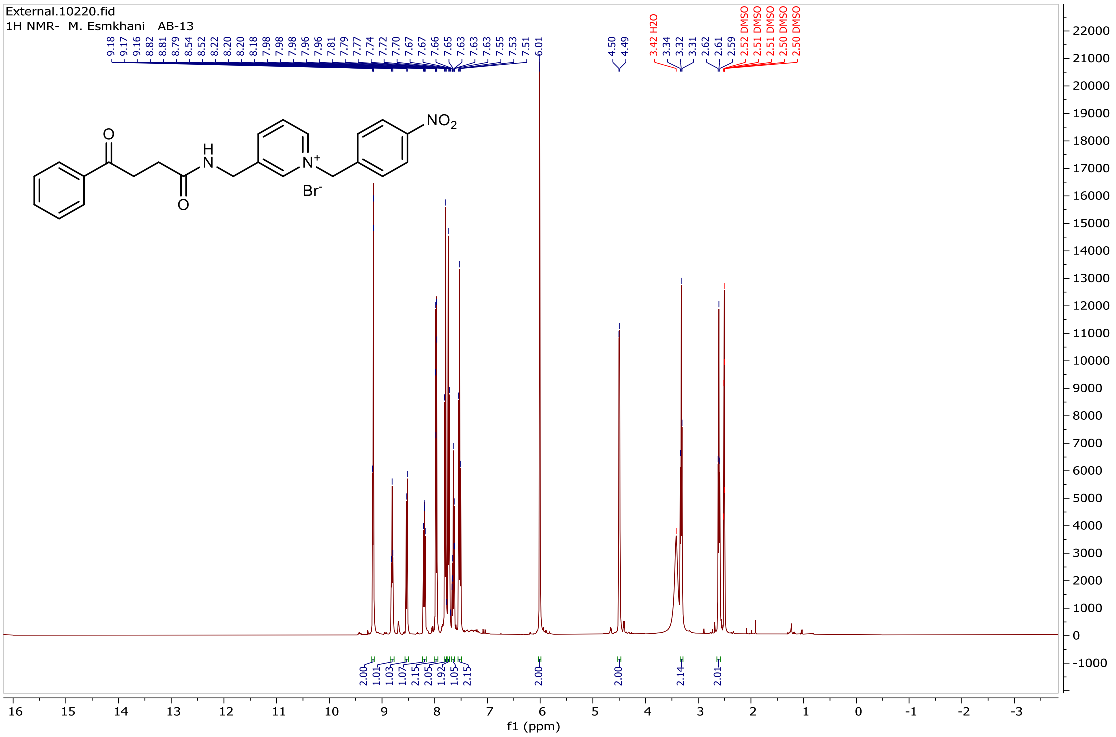

Figure S 21. ^{1}HNMR spectra of compound 7j.

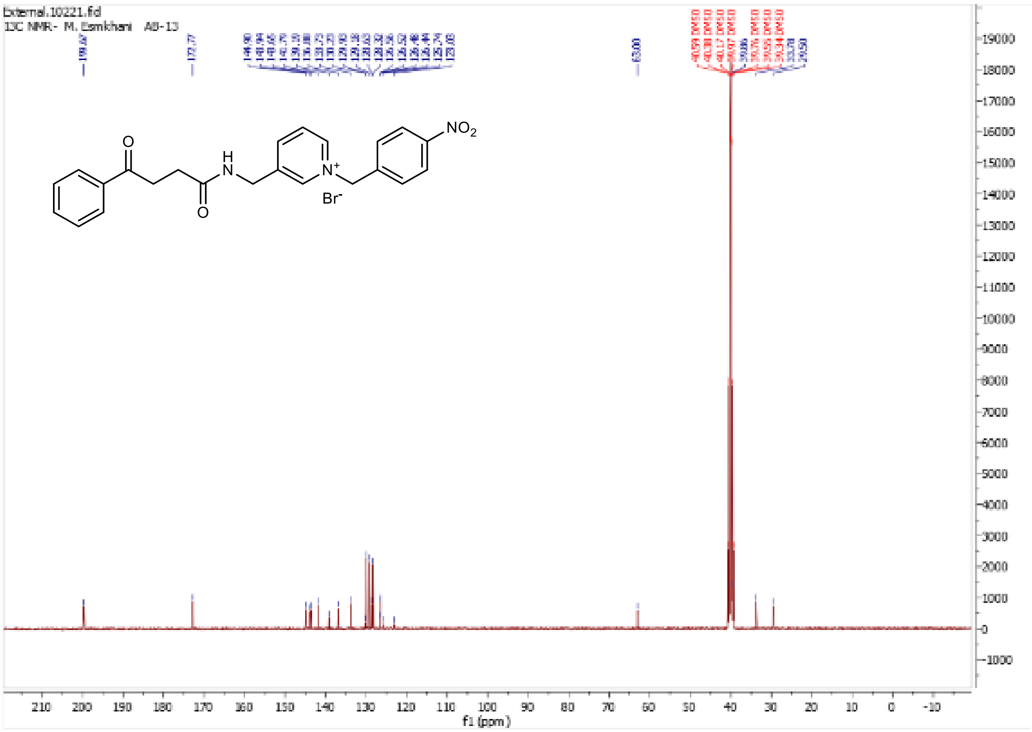

Figure S 22. ^{13}CNMR spectra of compound 7j.

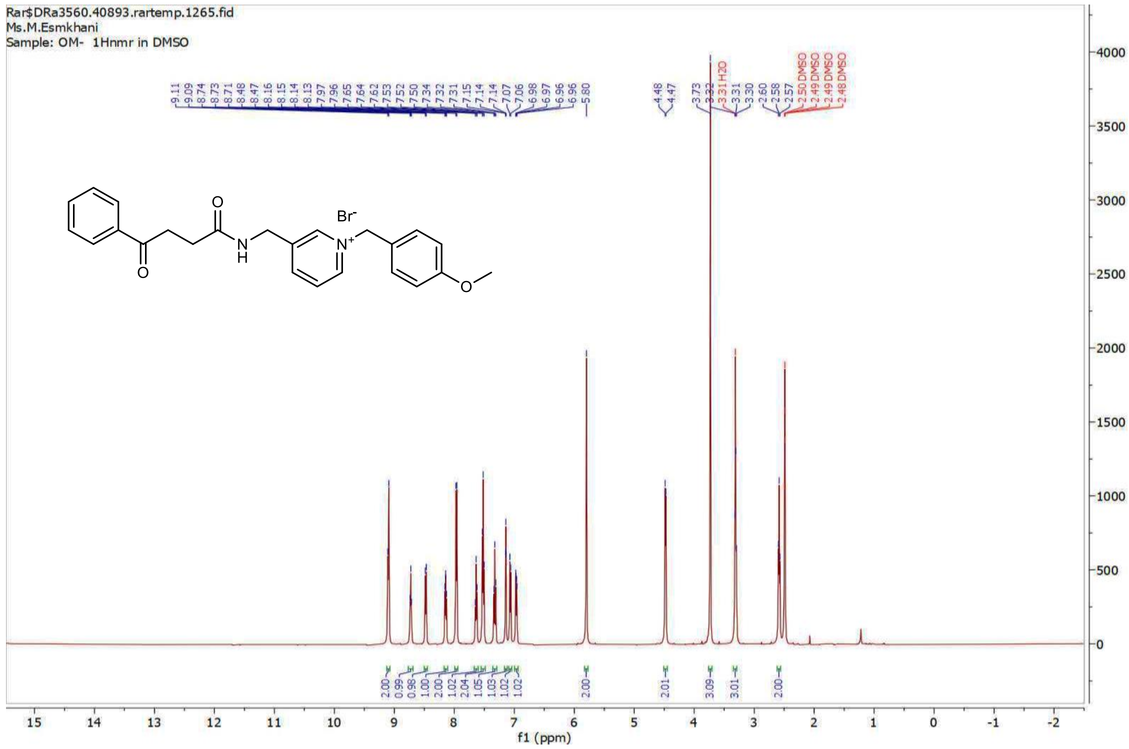

Figure S 23. ^{1}HNMR spectra of compound 7k.

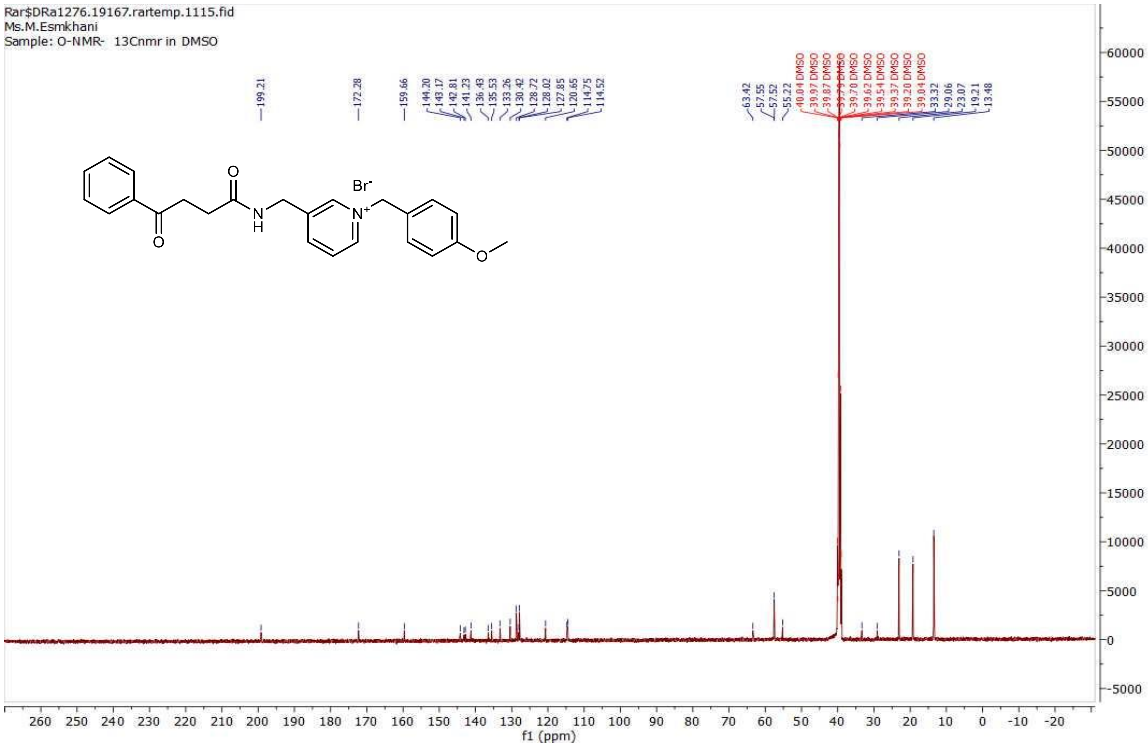

Figure S 24. ^{13}CNMR spectra of compound 7k.

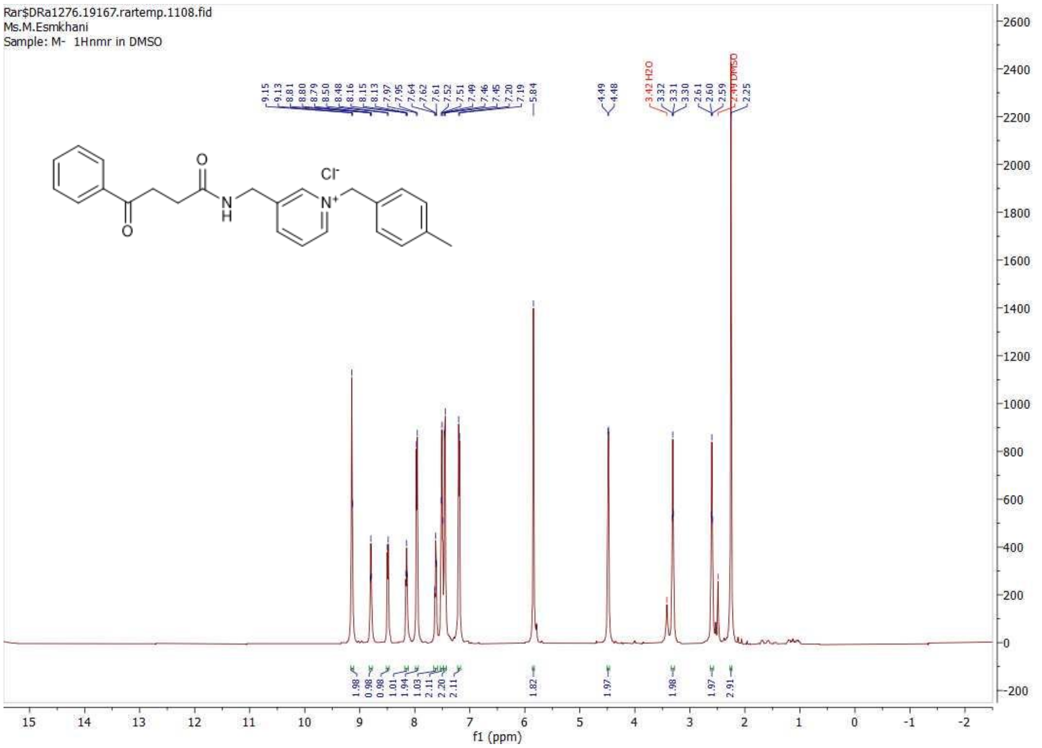

Figure S 25. ^{1}HNMR spectra of compound 7l.

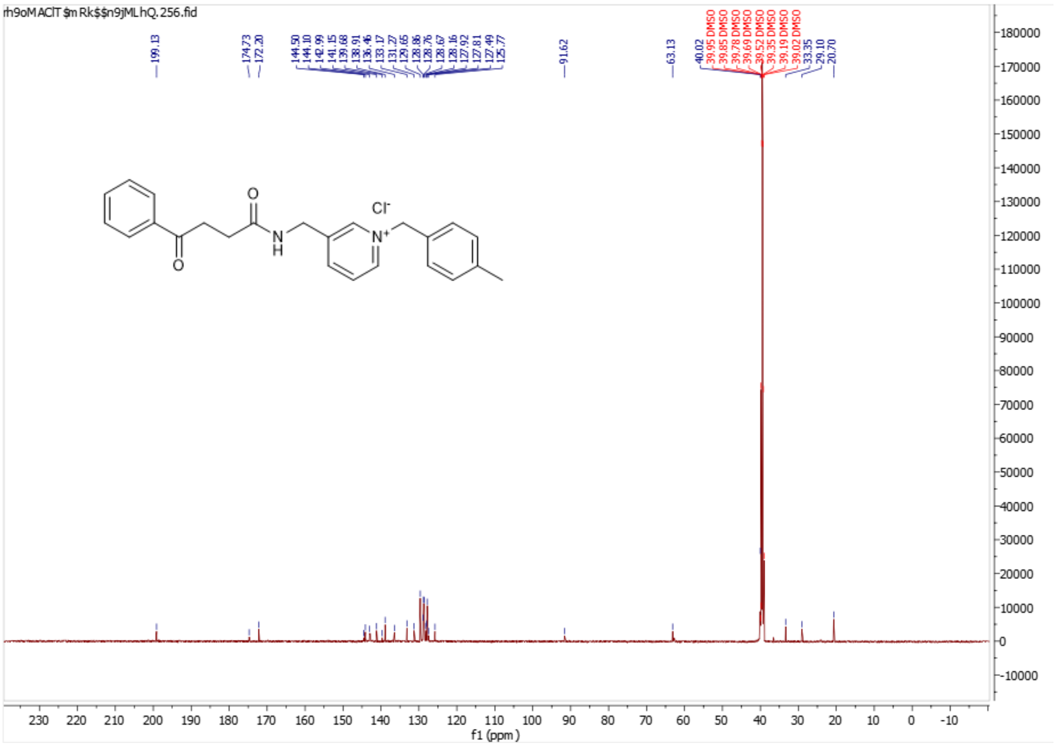

Figure S 26. ^{13}CNMR spectra of compound 7l.

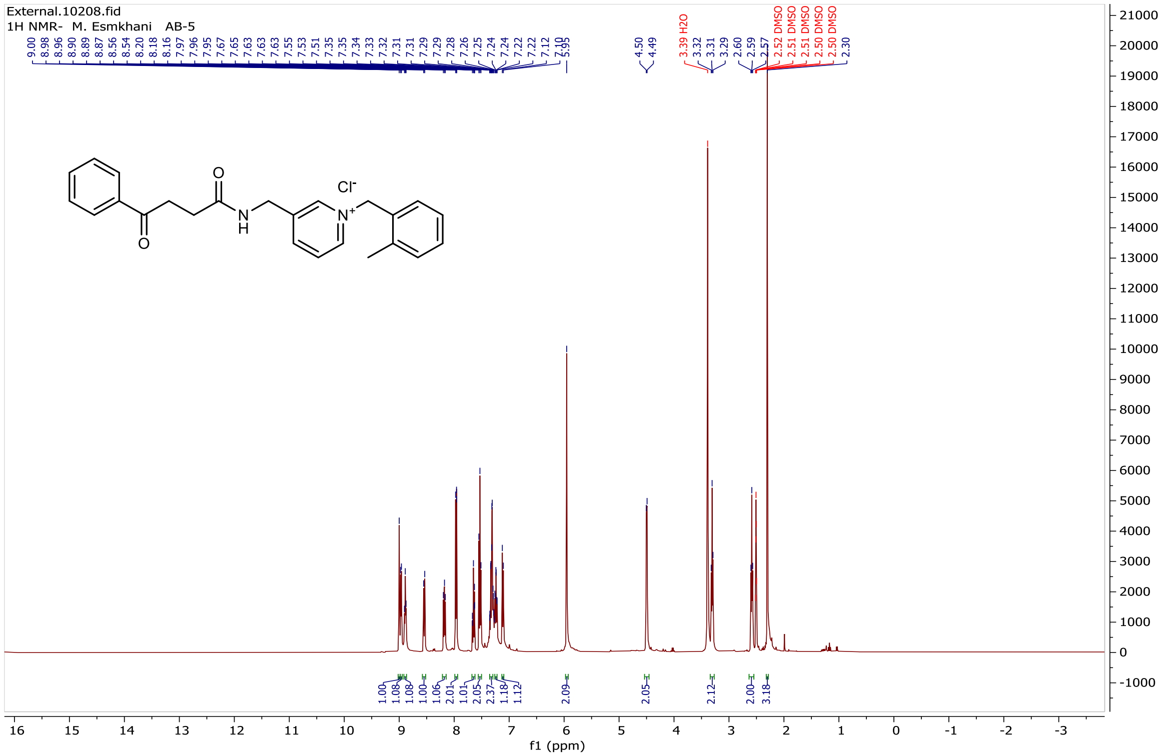

Figure S 27. ^{1}HNMR spectra of compound 7m.

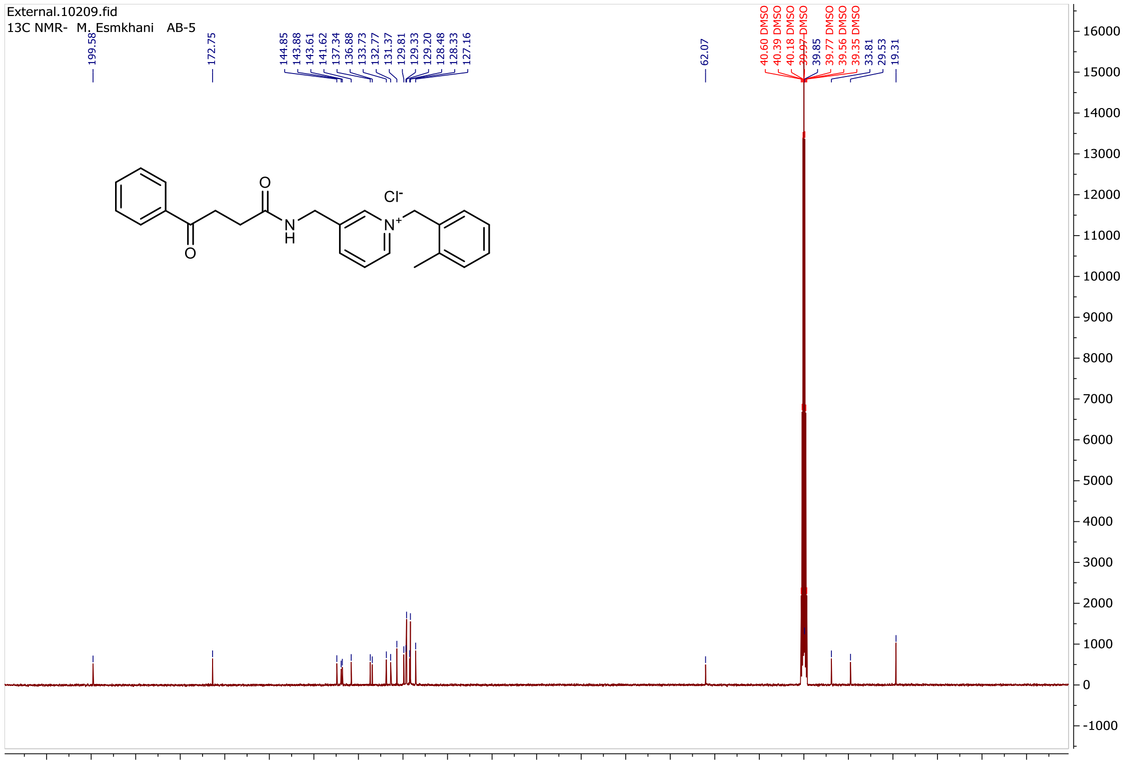

Figure S 28. ^{13}CNMR spectra of compound 7m.

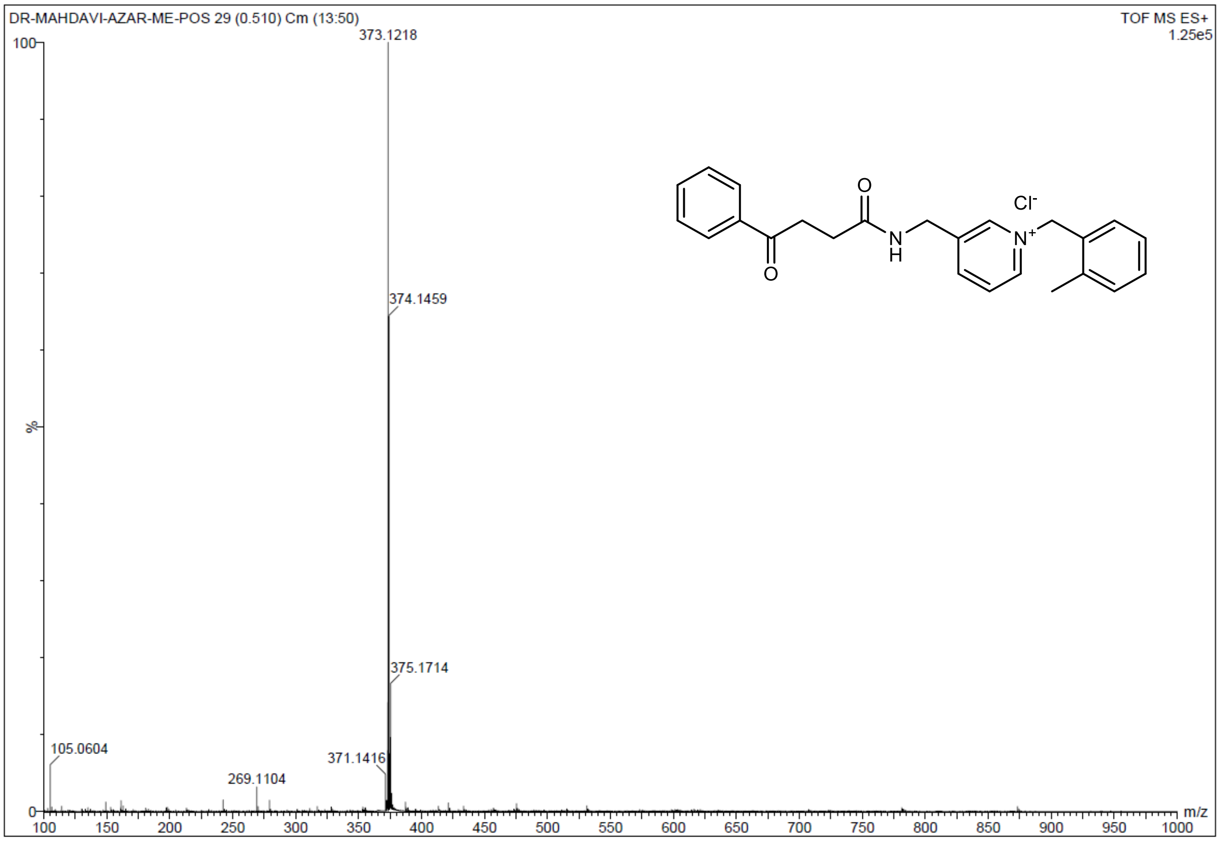

Figure S 29. HRMS spectra of compound 7m.

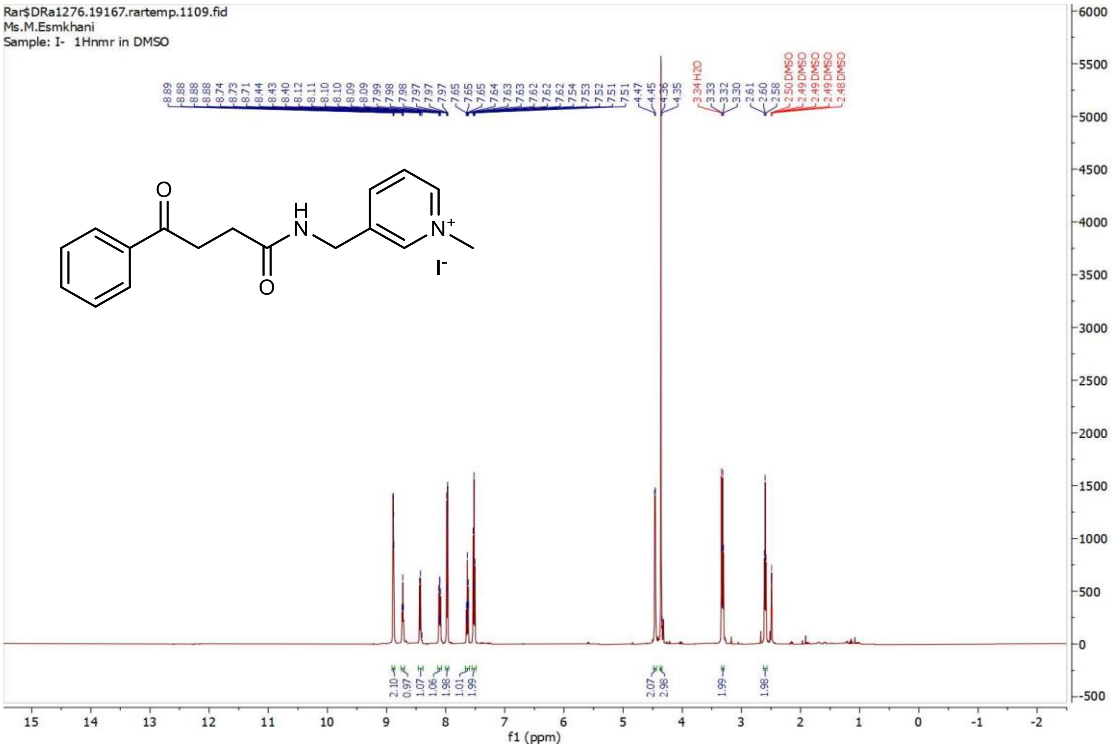

Figure S 30. ^{1}HNMR spectra of compound 7n.

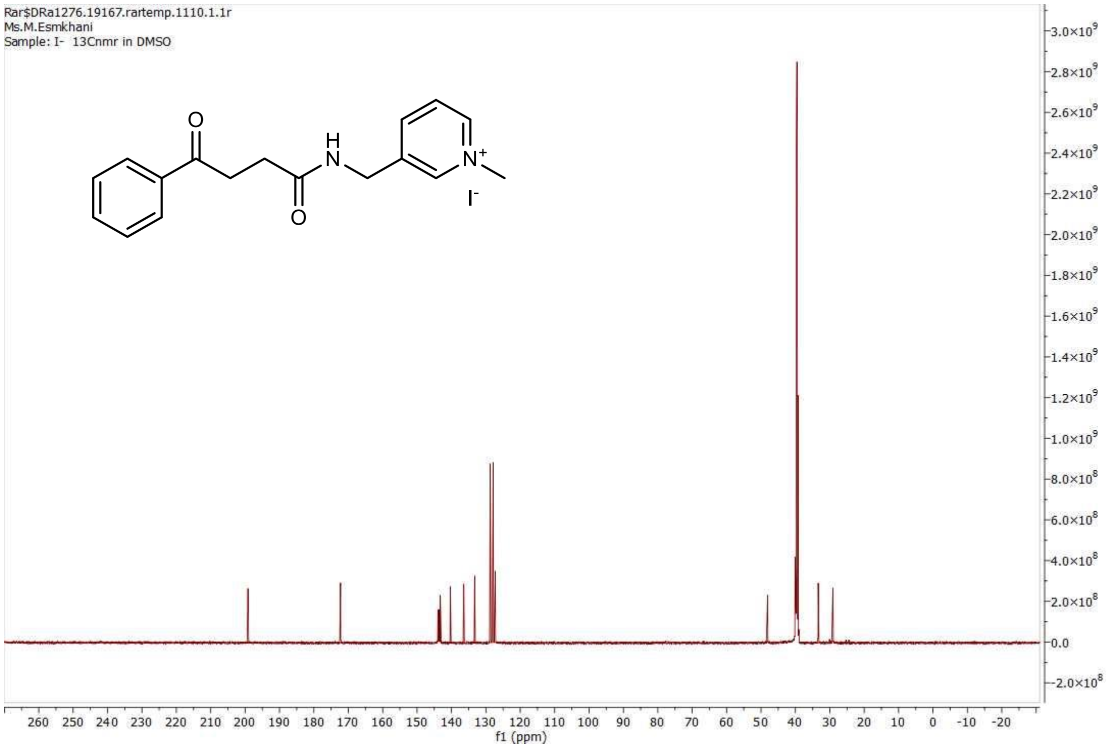

Figure S 31. ^{13}CNMR spectra of compound 7n

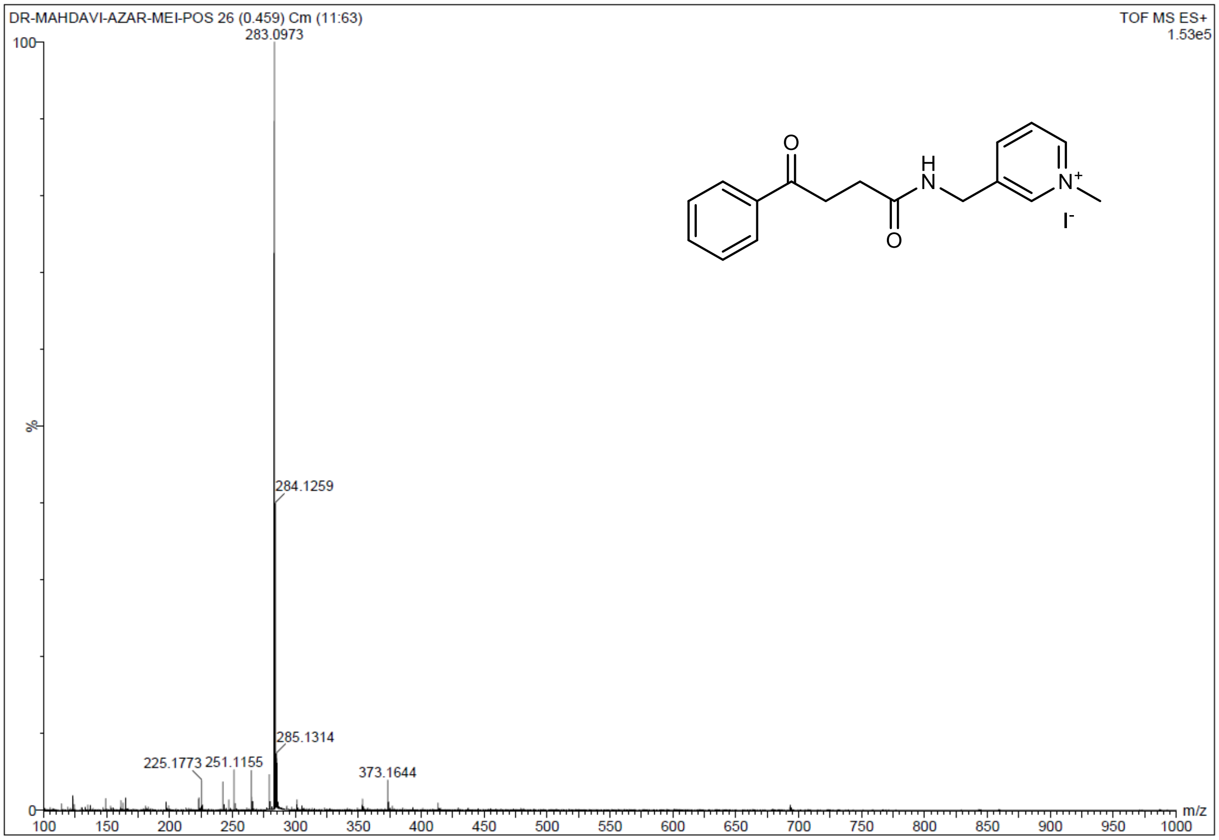

Figure S 32. HRMS spectra of compound 7n.

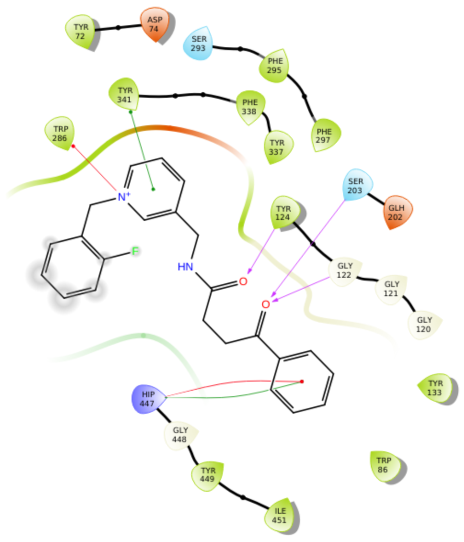

Figure S 33. Interaction of 7b in the active site of ACHE

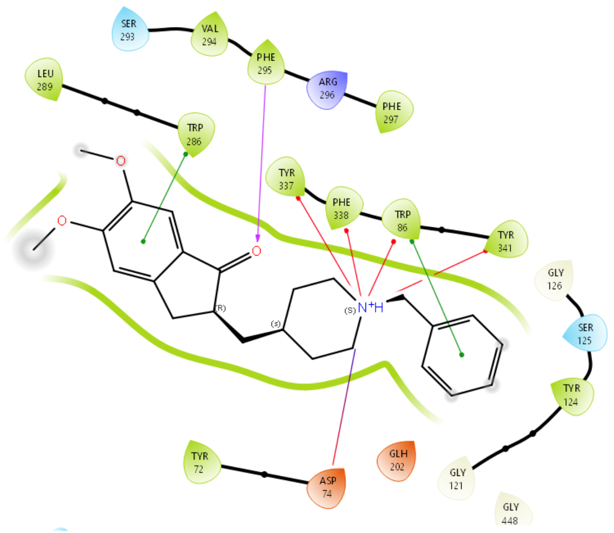

Figure S 34. 2D interaction of donepezil in the active site of ACHE

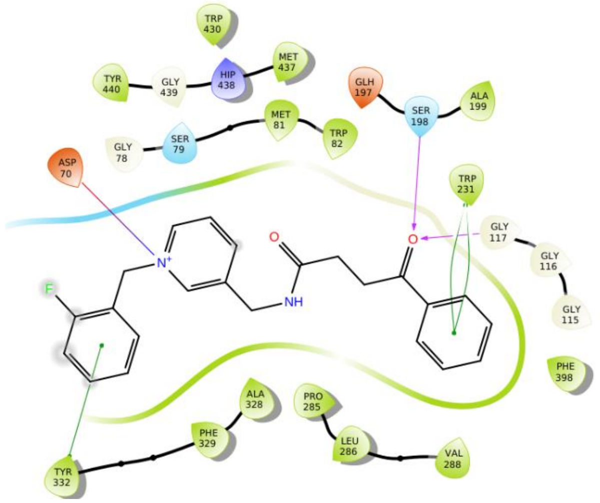

Figure S 35. 2D interaction of 7b in the active site of BCHE

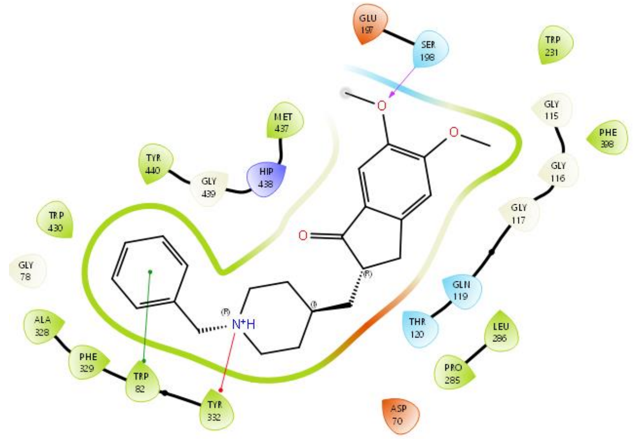

Figure S 36. 2D interaction of donepezil in the active site of BCHE

Table S1. *The IC$_{50}$ curve of **7a-n** against AChE*

<table>
<tr><td>7a</td><td>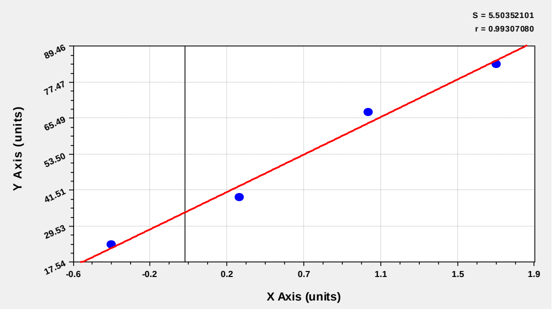
</td><td>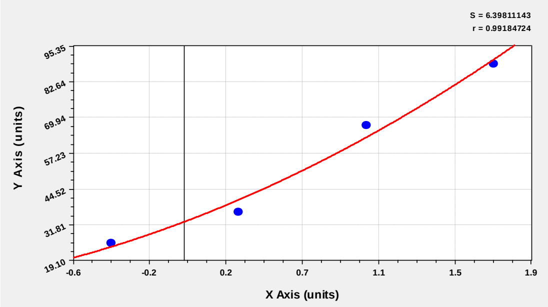
</td></tr>
<tr><td>7b</td><td>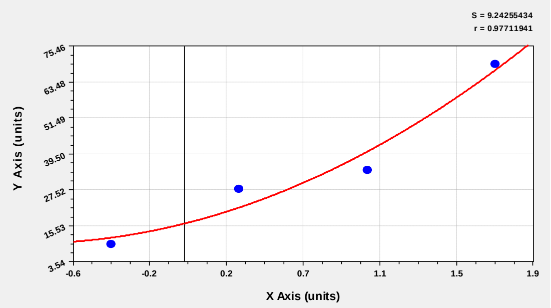
</td><td>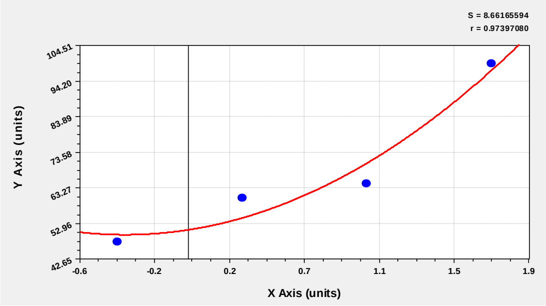
</td></tr>
<tr><td>7c</td><td>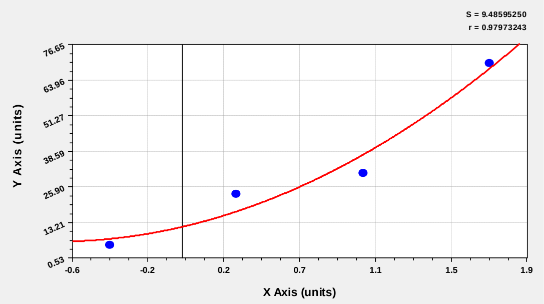
</td><td>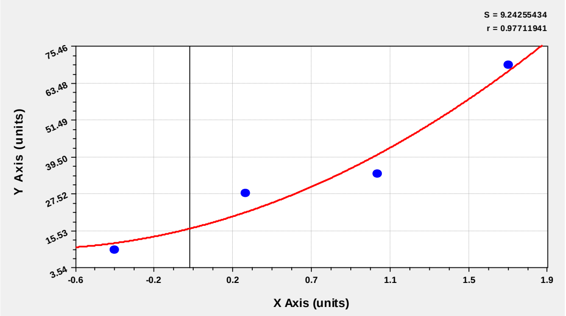
</td></tr>
<tr><td>7d</td><td>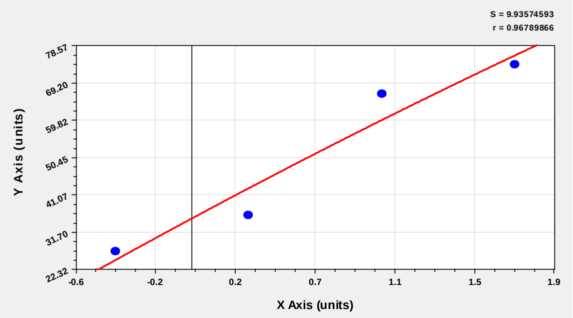
</td><td>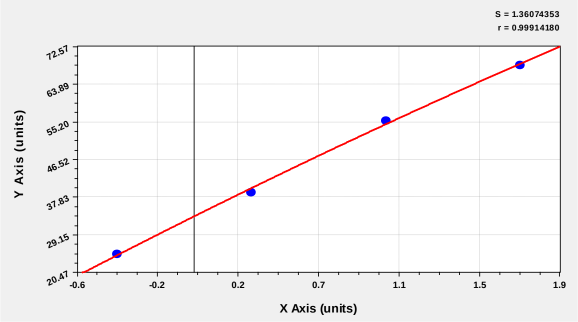
</td></tr>
</table>

7e

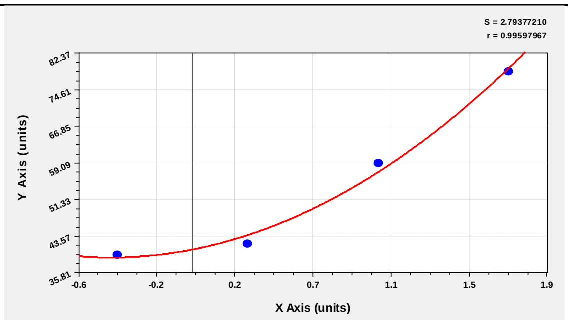
S = 2.79377210
r = 0.99597967
82.37
74.61
66.85
59.09
51.33
43.57
35.81
Y Axis (units)
-0.6
-0.2
0.2
0.7
1.1
1.5
1.9
X Axis (units)

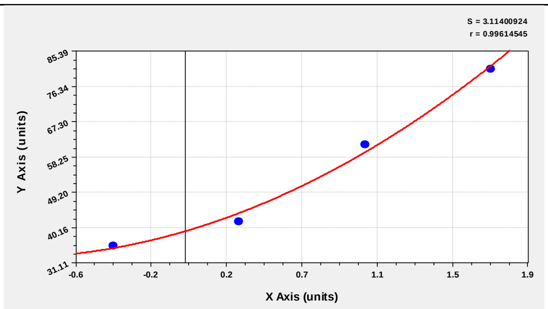
S = 3.11400924
r = 0.99614545
85.39
76.34
67.30
58.25
49.20
40.16
31.11
Y Axis (units)
-0.6
-0.2
0.2
0.7
1.1
1.5
1.9
X Axis (units)

7f

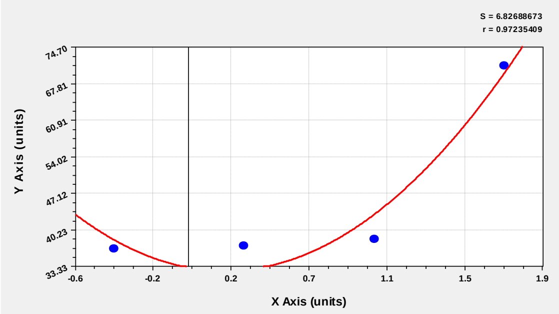
S = 6.82688673
r = 0.97235409
74.70
67.81
60.91
54.02
47.12
40.23
33.33
Y Axis (units)
-0.6
-0.2
0.2
0.7
1.1
1.5
1.9
X Axis (units)

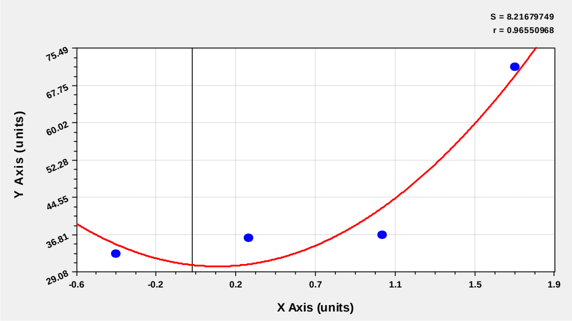
S = 8.21679749
r = 0.96550968
75.49
67.75
60.02
52.28
44.55
36.81
29.08
Y Axis (units)
-0.6
-0.2
0.2
0.7
1.1
1.5
1.9
X Axis (units)

7g

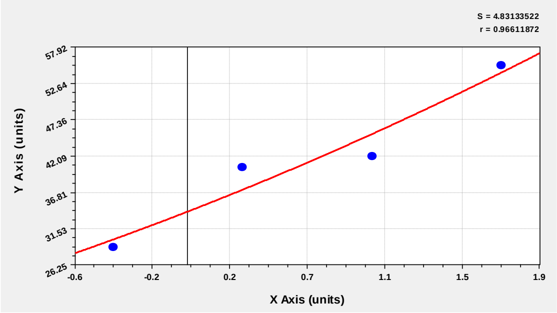
S = 4.83133522
r = 0.96611872
57.92
52.64
47.36
42.09
36.81
31.53
26.25
Y Axis (units)
-0.6
-0.2
0.2
0.7
1.1
1.5
1.9
X Axis (units)

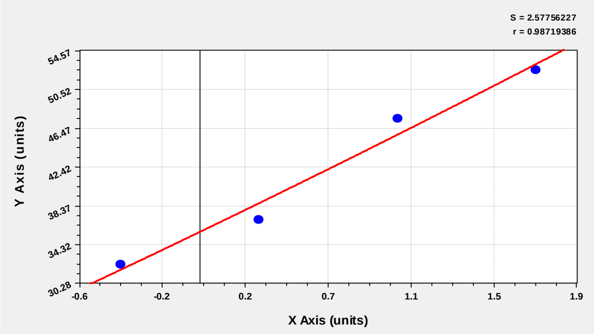
S = 2.57756227
r = 0.98719386
54.57
50.52
46.47
42.42
38.37
34.32
30.28
Y Axis (units)
-0.6
-0.2
0.2
0.7
1.1
1.5
1.9
X Axis (units)

7h

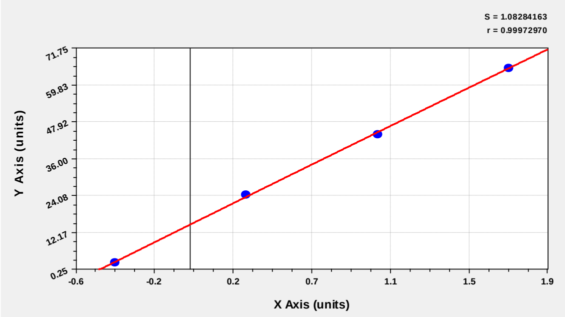
S = 1.08284163
r = 0.99972970
71.75
59.83
47.92
36.00
24.08
12.17
0.25
Y Axis (units)
-0.6
-0.2
0.2
0.7
1.1
1.5
1.9
X Axis (units)

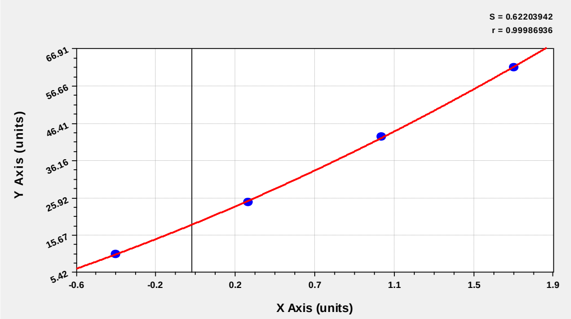
S = 0.62203942
r = 0.99986936
66.91
56.66
46.41
36.16
25.92
15.67
5.42
Y Axis (units)
-0.6
-0.2
0.2
0.7
1.1
1.5
1.9
X Axis (units)

7i

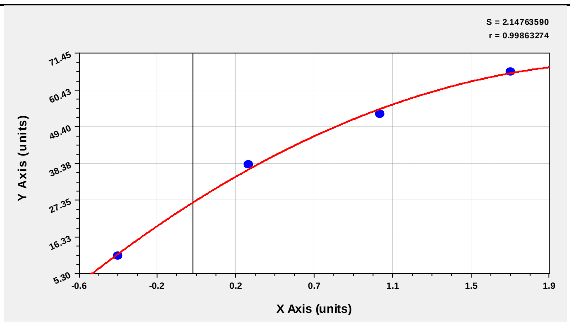
S = 2.14763590
r = 0.99863274
71.45
60.43
49.40
38.38
27.35
16.33
5.30
Y Axis (units)
-0.6
-0.2
0.2
0.7
1.1
1.5
1.9
X Axis (units)

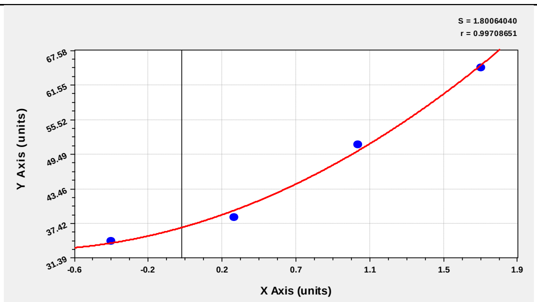
S = 1.80064040
r = 0.99708651
67.58
61.55
55.52
49.49
43.46
37.42
31.39
Y Axis (units)
-0.6
-0.2
0.2
0.7
1.1
1.5
1.9
X Axis (units)

7j	-	-
7k	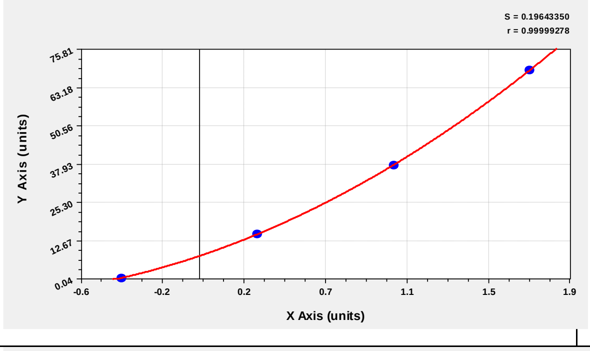S = 0.19643350<br>r = 0.99999278<br>Y Axis (units)<br>X Axis (units)<br>75.81<br>63.18<br>50.56<br>37.93<br>25.30<br>12.67<br>0.04<br>-0.6<br>-0.2<br>0.2<br>0.7<br>1.1<br>1.5<br>1.9	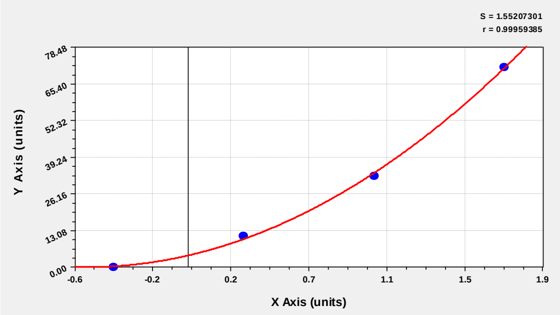S = 1.55207301<br>r = 0.99959385<br>Y Axis (units)<br>X Axis (units)<br>78.48<br>65.40<br>52.32<br>39.24<br>26.16<br>13.08<br>0.00<br>-0.6<br>-0.2<br>0.2<br>0.7<br>1.1<br>1.5<br>1.9
7l	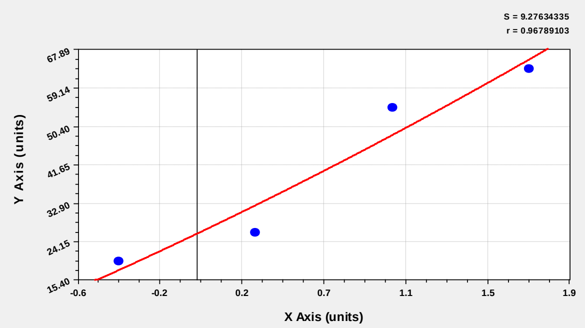S = 9.27634335<br>r = 0.96789103<br>Y Axis (units)<br>X Axis (units)<br>67.89<br>59.14<br>50.40<br>41.65<br>32.90<br>24.15<br>15.40<br>-0.6<br>-0.2<br>0.2<br>0.7<br>1.1<br>1.5<br>1.9	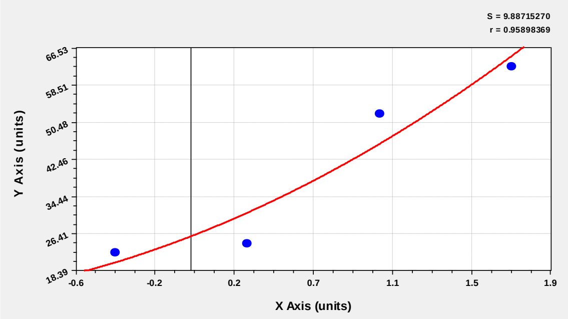S = 9.88715270<br>r = 0.95898369<br>Y Axis (units)<br>X Axis (units)<br>66.53<br>58.51<br>50.48<br>42.46<br>34.44<br>26.41<br>18.39<br>-0.6<br>-0.2<br>0.2<br>0.7<br>1.1<br>1.5<br>1.9
7 m	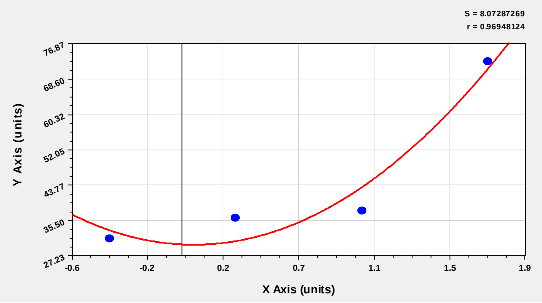S = 8.07287269<br>r = 0.96948124<br>Y Axis (units)<br>X Axis (units)<br>76.87<br>68.60<br>60.32<br>52.05<br>43.77<br>35.50<br>27.23<br>-0.6<br>-0.2<br>0.2<br>0.7<br>1.1<br>1.5<br>1.9	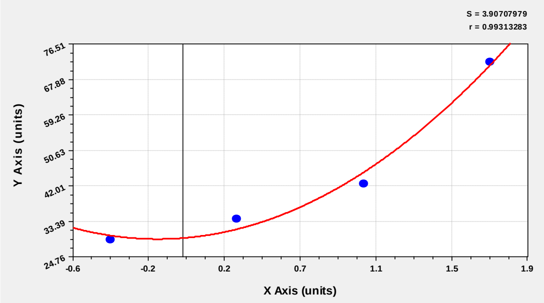S = 3.90707979<br>r = 0.99313283<br>Y Axis (units)<br>X Axis (units)<br>76.51<br>67.88<br>59.26<br>50.63<br>42.01<br>33.39<br>24.76<br>-0.6<br>-0.2<br>0.2<br>0.7<br>1.1<br>1.5<br>1.9
7n	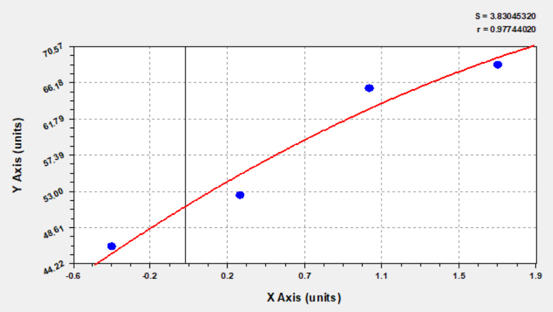S = 3.83045320<br>r = 0.97744020<br>Y Axis (units)<br>X Axis (units)<br>70.57<br>66.18<br>61.79<br>57.39<br>53.00<br>48.61<br>44.22<br>-0.6<br>-0.2<br>0.2<br>0.7<br>1.1<br>1.5<br>1.9	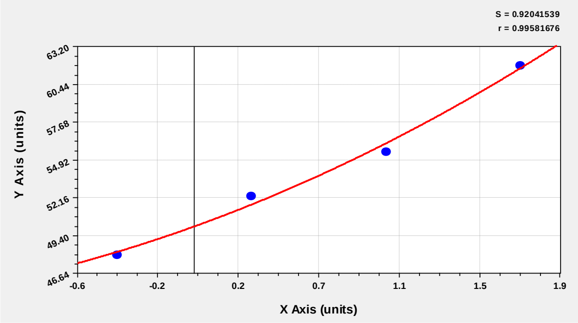S = 0.92041539<br>r = 0.99581676<br>Y Axis (units)<br>X Axis (units)<br>63.20<br>60.44<br>57.68<br>54.92<br>52.16<br>49.40<br>46.64<br>-0.6<br>-0.2<br>0.2<br>0.7<br>1.1<br>1.5<br>1.9

Table S2. *The IC_{50} curve of* ***7a-n*** *against BChE*

<table>
<tr><td>

7a

</td><td>

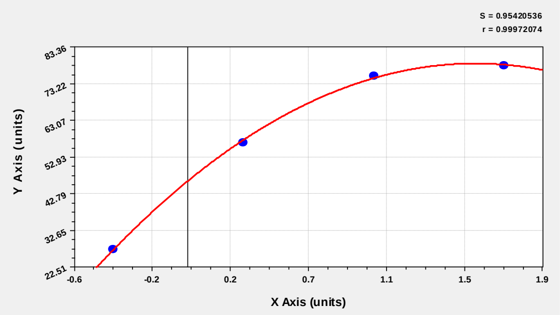

</td><td>

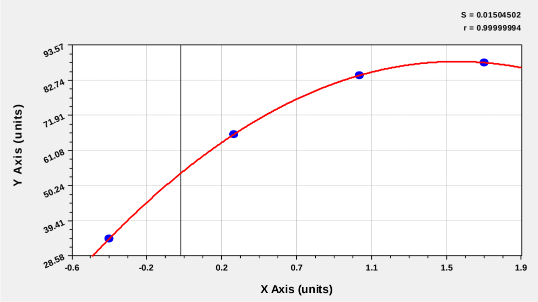

</td></tr>
<tr><td>

7b

</td><td>

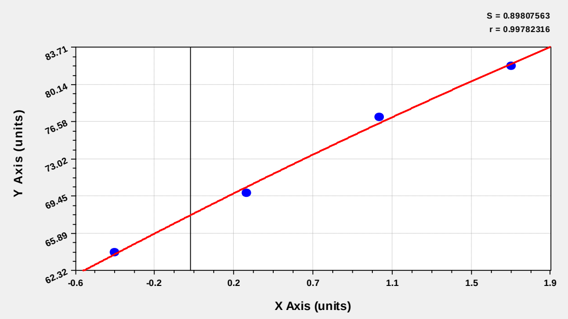

</td><td>

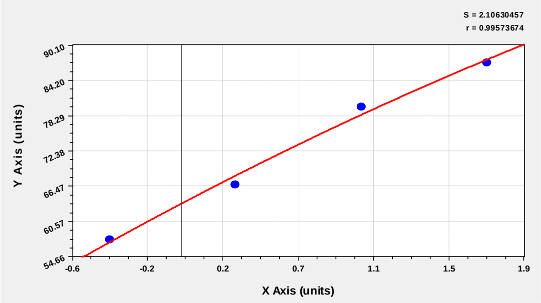

</td></tr>
<tr><td>

7c

</td><td>

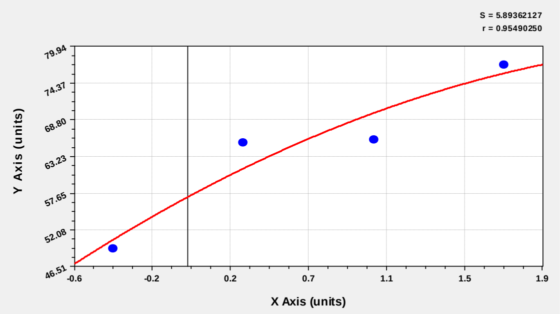

</td><td>

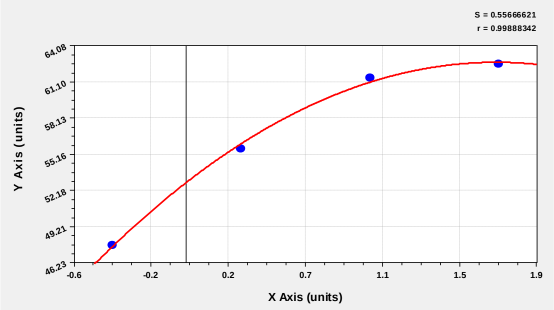

</td></tr>
<tr><td>

7d

</td><td>

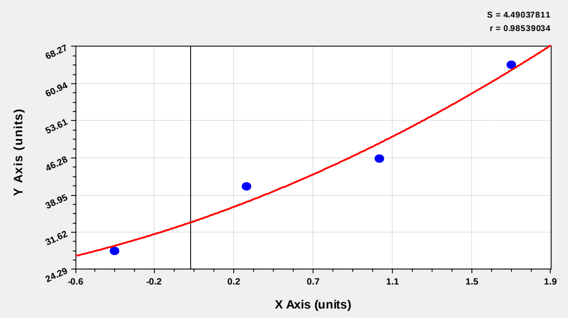

</td><td>

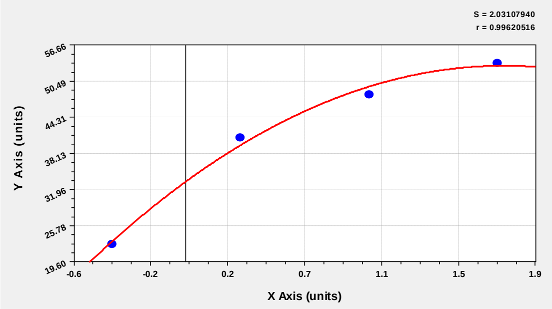

</td></tr>
</table>

7e

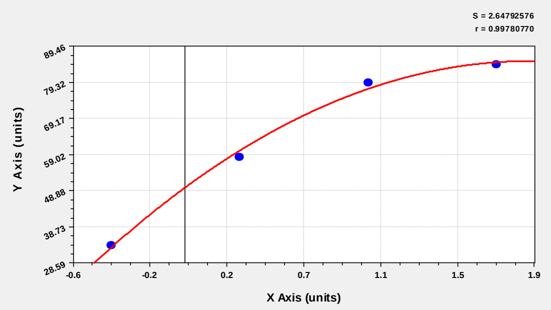

S = 2.64792576
r = 0.99780770
Y Axis (units)
89.46
79.32
69.17
59.02
48.88
38.73
28.59
X Axis (units)
-0.6
-0.2
0.2
0.7
1.1
1.5
1.9

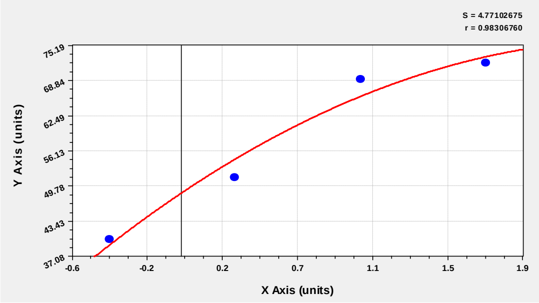

S = 4.77102675
r = 0.98306760
Y Axis (units)
75.19
68.84
62.49
56.13
49.78
43.43
37.08
X Axis (units)
-0.6
-0.2
0.2
0.7
1.1
1.5
1.9

7f

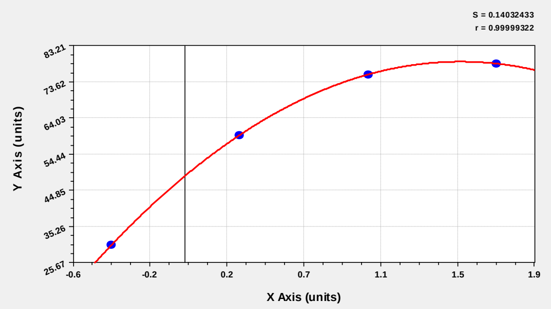

S = 0.14032433
r = 0.99999322
Y Axis (units)
83.21
73.62
64.03
54.44
44.85
35.26
25.67
X Axis (units)
-0.6
-0.2
0.2
0.7
1.1
1.5
1.9

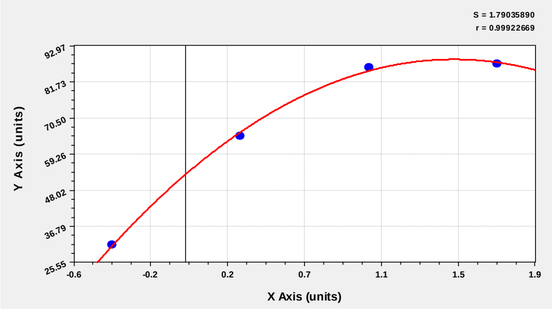

S = 1.79035890
r = 0.99922669
Y Axis (units)
92.97
81.73
70.50
59.26
48.02
36.79
25.55
X Axis (units)
-0.6
-0.2
0.2
0.7
1.1
1.5
1.9

7g

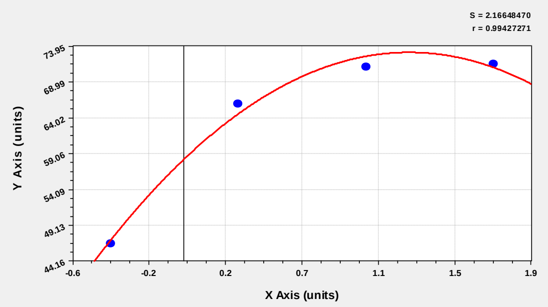

S = 2.16648470
r = 0.99427271
Y Axis (units)
73.95
68.99
64.02
59.06
54.09
49.13
44.16
X Axis (units)
-0.6
-0.2
0.2
0.7
1.1
1.5
1.9

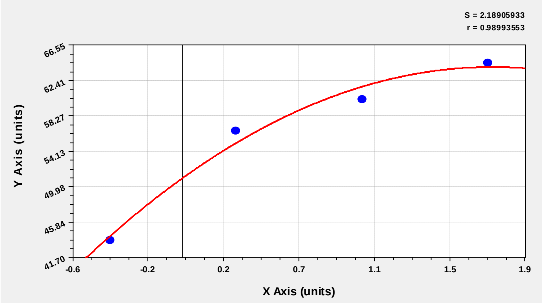

S = 2.18905933
r = 0.98993553
Y Axis (units)
66.55
62.41
58.27
54.13
49.98
45.84
41.70
X Axis (units)
-0.6
-0.2
0.2
0.7
1.1
1.5
1.9

7h

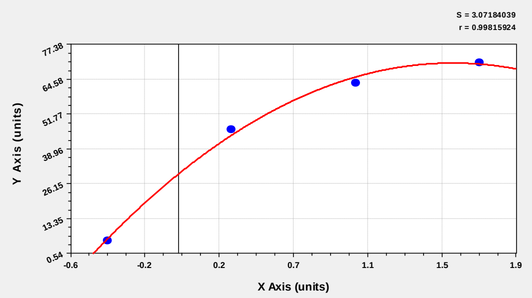

S = 3.07184039
r = 0.99815924
Y Axis (units)
77.38
64.58
51.77
38.96
26.15
13.35
0.54
X Axis (units)
-0.6
-0.2
0.2
0.7
1.1
1.5
1.9

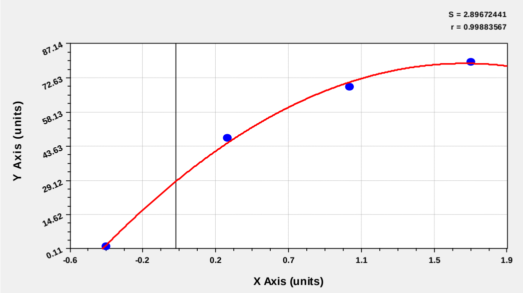

S = 2.89672441
r = 0.99883567
Y Axis (units)
87.14
72.63
58.13
43.63
29.12
14.62
0.11
X Axis (units)
-0.6
-0.2
0.2
0.7
1.1
1.5
1.9

7i

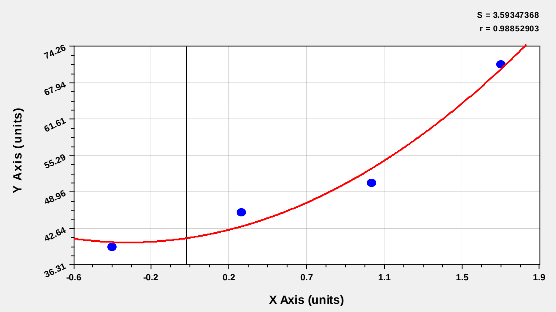

S = 3.59347368
r = 0.98852903
Y Axis (units)
74.26
67.94
61.61
55.29
48.96
42.64
36.31
X Axis (units)
-0.6
-0.2
0.2
0.7
1.1
1.5
1.9

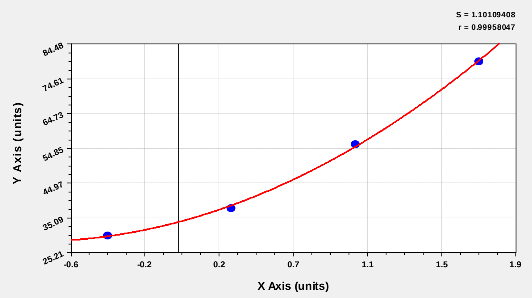

S = 1.10109408
r = 0.99958047
Y Axis (units)
84.48
74.61
64.73
54.85
44.97
35.09
25.21
X Axis (units)
-0.6
-0.2
0.2
0.7
1.1
1.5
1.9

7j

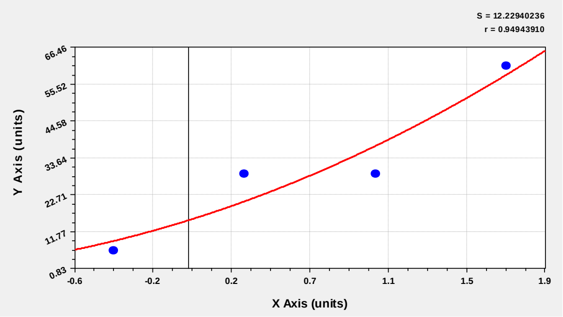

S = 12.22940236
r = 0.94943910
Y Axis (units)
66.46
55.52
44.58
33.64
22.71
11.77
0.83
-0.6
-0.2
0.2
0.7
1.1
1.5
1.9
X Axis (units)

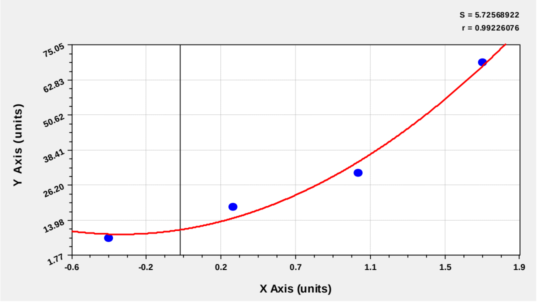

S = 5.72568922
r = 0.99226076
Y Axis (units)
75.05
62.83
50.62
38.41
26.20
13.98
1.77
-0.6
-0.2
0.2
0.7
1.1
1.5
1.9
X Axis (units)

7k

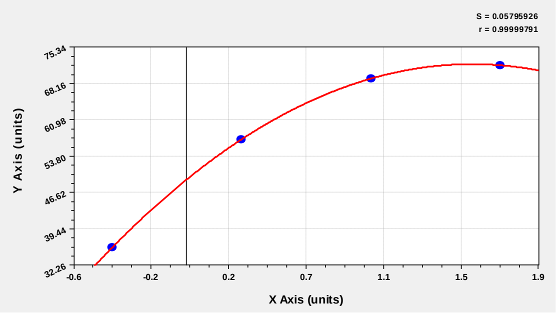

S = 0.05795926
r = 0.99999791
Y Axis (units)
75.34
68.16
60.98
53.80
46.62
39.44
32.26
-0.6
-0.2
0.2
0.7
1.1
1.5
1.9
X Axis (units)

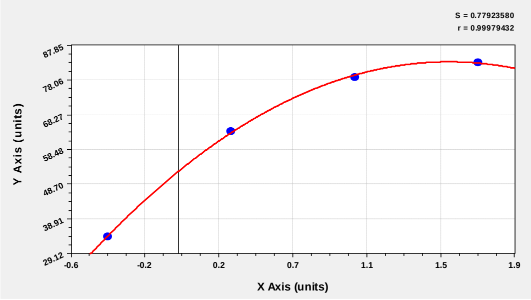

S = 0.77923580
r = 0.99979432
Y Axis (units)
87.85
78.06
68.27
58.48
48.70
38.91
29.12
-0.6
-0.2
0.2
0.7
1.1
1.5
1.9
X Axis (units)

7l

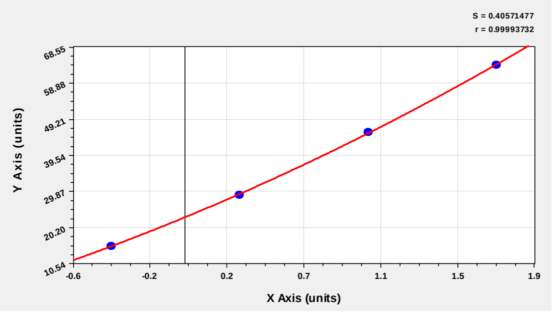

S = 0.40571477
r = 0.99993732
Y Axis (units)
68.55
58.88
49.21
39.54
29.87
20.20
10.54
-0.6
-0.2
0.2
0.7
1.1
1.5
1.9
X Axis (units)

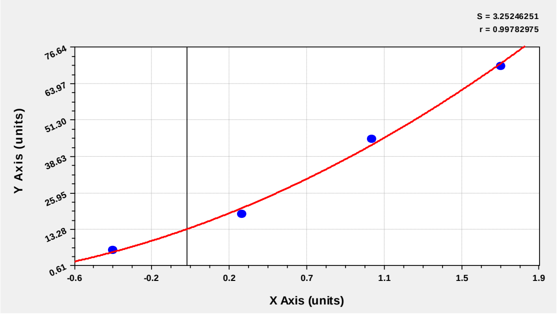

S = 3.25246251
r = 0.99782975
Y Axis (units)
76.64
63.97
51.30
38.63
25.95
13.28
0.61
-0.6
-0.2
0.2
0.7
1.1
1.5
1.9
X Axis (units)

7 m

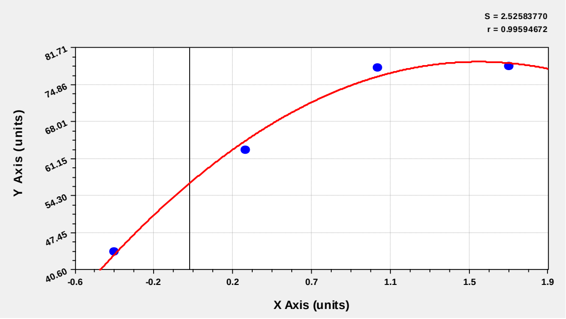

S = 2.52583770
r = 0.99594672
Y Axis (units)
81.71
74.86
68.01
61.15
54.30
47.45
40.60
-0.6
-0.2
0.2
0.7
1.1
1.5
1.9
X Axis (units)

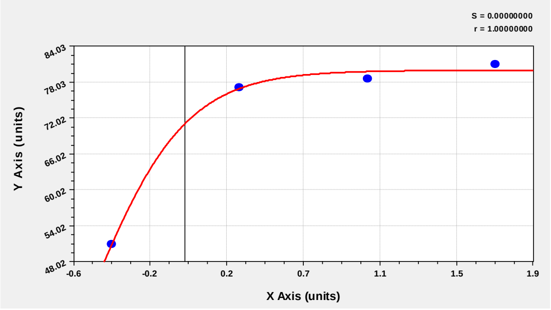

S = 0.00000000
r = 1.00000000
Y Axis (units)
84.03
78.03
72.02
66.02
60.02
54.02
48.02
-0.6
-0.2
0.2
0.7
1.1
1.5
1.9
X Axis (units)

7n

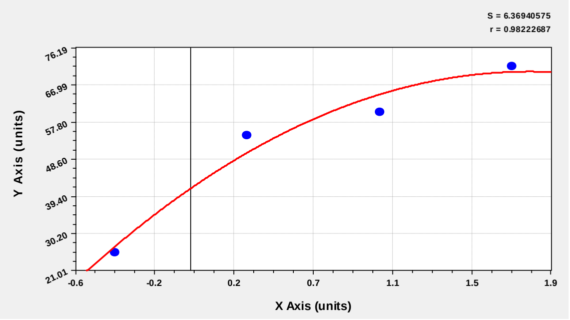

S = 6.36940575
r = 0.98222687
Y Axis (units)
76.19
66.99
57.80
48.60
39.40
30.20
21.01
-0.6
-0.2
0.2
0.7
1.1
1.5
1.9
X Axis (units)

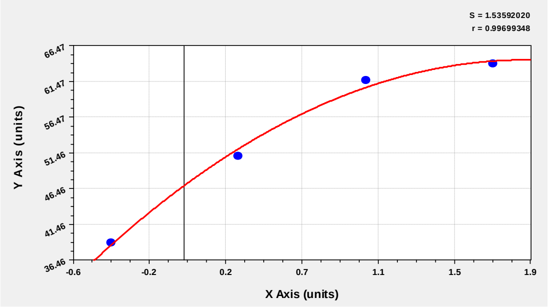

S = 1.53592020
r = 0.99699348
Y Axis (units)
66.47
61.47
56.47
51.46
46.46
41.46
36.46
-0.6
-0.2
0.2
0.7
1.1
1.5
1.9
X Axis (units)
